# Supplementary material for: Immunomodulatory effects and improved outcomes with cisplatin- versus carboplatin-based chemotherapy plus atezolizumab in urothelial cancer
Source: Cell Rep Med. 2024 Jan 26;5(2):101393. doi: 10.1016/j.xcrm.2024.101393 (PMC10897541; doi:10.1016/j.xcrm.2024.101393)
Supplement: Document S1. Tables S1 and S2 and Figures S1–S10 [file mmc1.pdf]

Cell Reports Medicine, Volume *5*

Supplemental information

Immunomodulatory effects and improved outcomes with cisplatin- versus carboplatin-based chemotherapy plus atezolizumab in urothelial cancer

Matthew D. Galsky, Xiangnan Guan, Deepali Rishipathak, Aaron S. Rapaport, Hesham M. Shehata, Romain Banchereau, Kobe Yuen, Eugene Varfolomeev, Ruozhen Hu, Chia-Jung Han, Haocheng Li, Yuxin Liang, Domagoj Vucic, Li Wang, Jun Zhu, Haocheng Yu, Rebecca H. Herbst, Emma Hajaj, Evgeny Kiner, Aristotelis Bamias, Maria De Santis, Ian D. Davis, José Ángel Arranz, Eiji Kikuchi, Sandrine Bernhard, Patrick Williams, Chooi Lee, Ira Mellman, Shomyseh Sanjabi, Robert Johnston, Peter C. Black, Enrique Grande, and Sanjeev Mariathasan

Table S1. Baseline patient characteristics across different treatment arms in the CITE-Seq cohort. Related to Figure 1.

	GemCis (n=19)	GemCarbo (n=19)	Atezolizumab (n=33)	GemCis + Atezolizumab (n=15)	GemCarbo + Atezolizumab (n=27)
Age,years	62(50-74)	66(45-87)	66(51-81)	65(53.5-76.5)	67(58-76)
Age group,years					
<65	10(53%)	8(42%)	16(48%)	7(47%)	10(37%)
>=65	9(47%)	11(58%)	17(52%)	8(53%)	17(63%)
Gender					
Female	1(5%)	11(58%)	4(12%)	5(33%)	5(19%)
Male	18(95%)	8(42%)	29(88%)	10(67%)	22(81%)
Race					
White	17(89%)	15(79%)	22(67%)	13(87%)	23(85%)
Black or African American	0(<1%)	0(<1%)	0(<1%)	0(<1%)	0(<1%)
Asian	2(11%)	4(21%)	11(33%)	2(13%)	3(11%)
American Indian or Alaska Native	0(<1%)	0(<1%)	0(<1%)	0(<1%)	1(4%)
Native Hawaiian or other Pacific Islander	0(<1%)	0(<1%)	0(<1%)	0(<1%)	0(<1%)
Multiple races	0(<1%)	0(<1%)	0(<1%)	0(<1%)	0(<1%)
Unknown	0(<1%)	0(<1%)	0(<1%)	0(<1%)	0(<1%)
Tobacco use history					
Never	9(47%)	8(42%)	7(21%)	6(40%)	8(30%)
Current	4(21%)	1(5%)	7(21%)	4(27%)	6(22%)
Former	6(32%)	10(53%)	19(58%)	5(33%)	13(48%)
Primary tumor site					
Bladder	16(84%)	13(68%)	26(79%)	11(73%)	19(70%)
Urethra	0(<1%)	0(<1%)	1(3%)	1(7%)	0(<1%)
Renal pelvis	1(5%)	3(16%)	5(15%)	1(7%)	6(22%)
Ureter	2(11%)	1(5%)	1(3%)	2(13%)	2(7%)
Other	0(<1%)	2(11%)	0(<1%)	0(<1%)	0(<1%)
Disease status					
Locally advanced	0(<1%)	1(5%)	2(6%)	1(7%)	3(11%)
Metastatic	19(100%)	18(95%)	31(94%)	14(93%)	24(89%)
Site of metastatic disease					
Lymph node only	5(26%)	0(<1%)	7(21%)	4(27%)	3(11%)
Visceral	12(63%)	13(68%)	22(67%)	8(53%)	18(67%)
Liver	7(37%)	7(37%)	9(27%)	1(7%)	11(41%)
ECOG performance status					
0	11(58%)	4(21%)	15(45%)	5(33%)	8(30%)
1	7(37%)	13(68%)	16(48%)	10(67%)	13(48%)
2	1(5%)	2(11%)	2(6%)	0(<1%)	6(22%)
Programmed death-ligand 1 status on immune cells					
IC2/3	5(26%)	3(16%)	11(33%)	4(27%)	5(19%)
IC0/1	14(74%)	16(84%)	22(67%)	11(73%)	22(81%)

Data are n (%) unless indicated otherwise.

Table S2. Comparison of baseline characteristics in the CITE-Seq (n = 113) versus non–CITE-Seq cohort (N = 1100). Related to Figure 1.

	Non-scSeq patients (n=1100)	scSeq patients (n=113)
Age,years	68(56-80)	66(52-80)
Age group,years		
<65	396(36%)	51(45%)
>=65	704(64%)	62(55%)
Gender		
Female	270(25%)	26(23%)
Male	830(75%)	87(77%)
Race		
White	821(75%)	90(80%)
Black or African American	7(1%)	0(<1%)
Asian	247(22%)	22(19%)
American Indian or Alaska Native	8(1%)	1(1%)
Native Hawaiian or other Pacific Islander	1(<1%)	0(<1%)
Multiple races	1(<1%)	0(<1%)
Unknown	15(1%)	0(<1%)
Tobacco use history		
Never	403(37%)	38(34%)
Current	174(16%)	22(19%)
Former	523(48%)	53(47%)
Primary tumor site		
Bladder	782(71%)	85(75%)
Urethra	19(2%)	2(2%)
Renal pelvis	161(15%)	16(14%)
Ureter	129(12%)	8(7%)
Other	9(1%)	2(2%)
Disease status		
Locally advanced	121(11%)	7(6%)
Metastatic	979(89%)	106(94%)
Site of metastatic disease		
Lymph node only	199(18%)	19(17%)
Visceral	627(57%)	73(65%)
Liver	237(22%)	35(31%)
ECOG performance status		
0	469(43%)	43(38%)
1	511(46%)	59(52%)
2	120(11%)	11(10%)
Programmed death-ligand 1 status on immune cells		
IC2/3	256(23%)	28(25%)
IC0/1	844(77%)	85(75%)

Data are n (%) unless indicated otherwise.

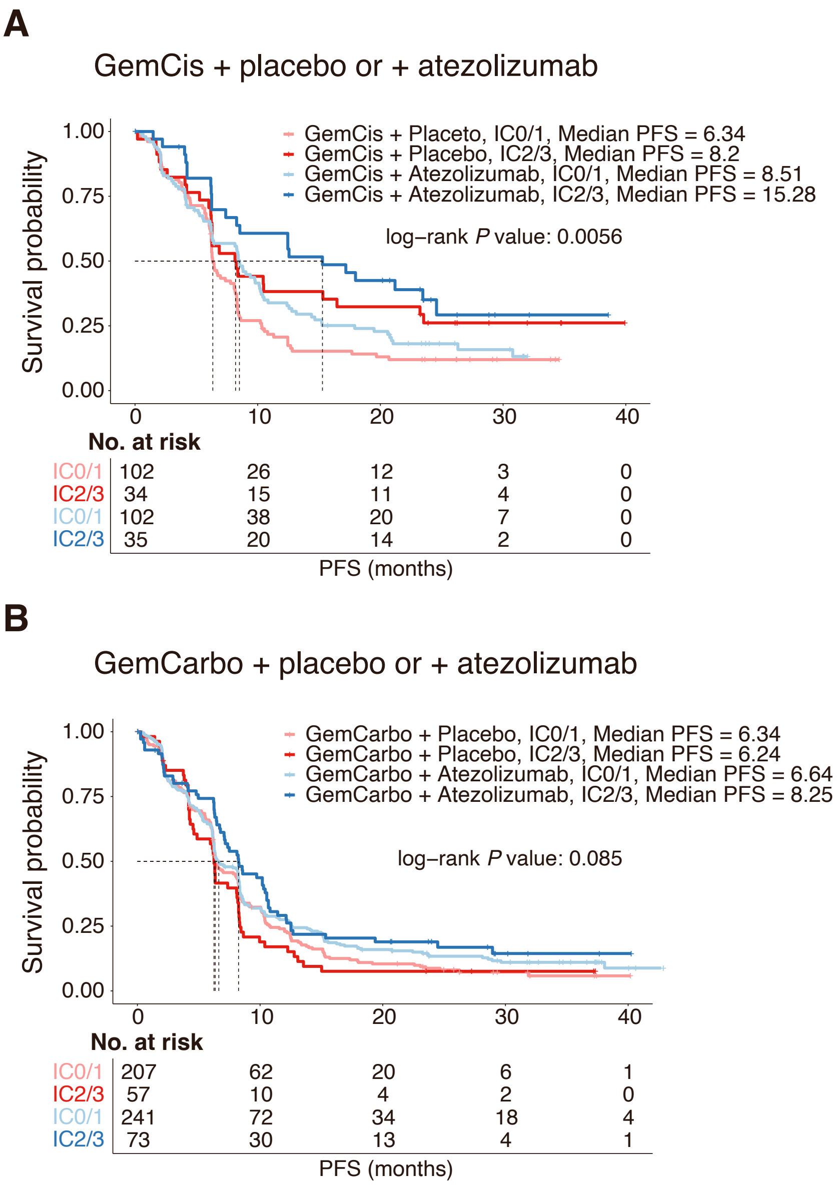

Figure S1. Progression-free survival outcomes with GemCis, but not with GemCarbo, are dependent on pre-treatment tumor PD-L1 expression. Related to Figure 1.

(A-B) Kaplan-Meier curves showing progression-free survival in patients receiving GemCis plus atezolizumab or placebo (A) or GemCarbo plus atezolizumab or placebo (B), stratified by tumor PD-L1 expression as measured by SP142 staining. *P* values were estimated using the log-rank test.

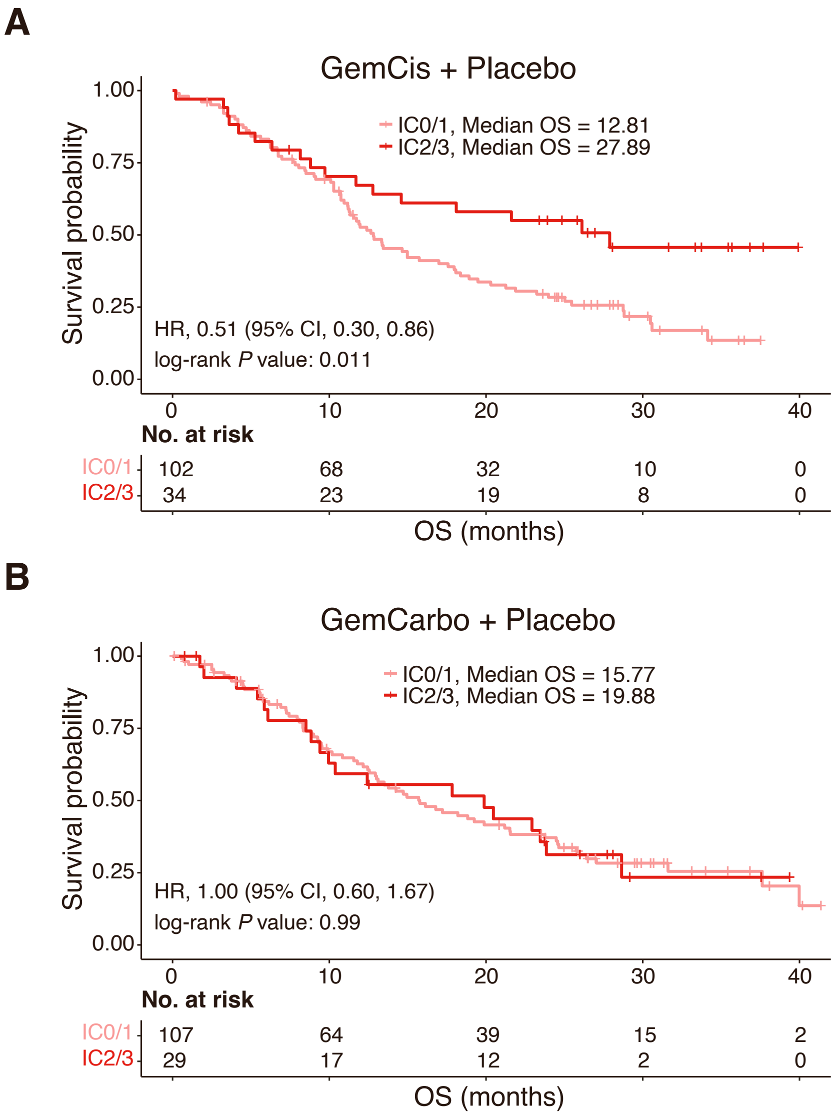

Figure S2. Survival outcomes with GemCis, but not with GemCarbo, are dependent on pre-treatment tumor PD-L1 expression in matched patients. Related to Figure 1.

(A-B) Kaplan-Meier curves showing overall survival in matched patients in arm C stratified by PD-L1 status (IC2/3 versus IC0/1) and use of GemCis (A) or GemCarbo (B). Cisplatin-eligible patients receiving GemCis or GemCarbo were matched 1:1 by propensity scores calculated using age, ECOG, Bajorin risk factors, and the site of metastasis variables. Patients whose propensity scores cannot be matched were discarded. *P* values were calculated using the log-rank test. Hazard ratio and 95% confidence interval were estimated using a univariate Cox model.

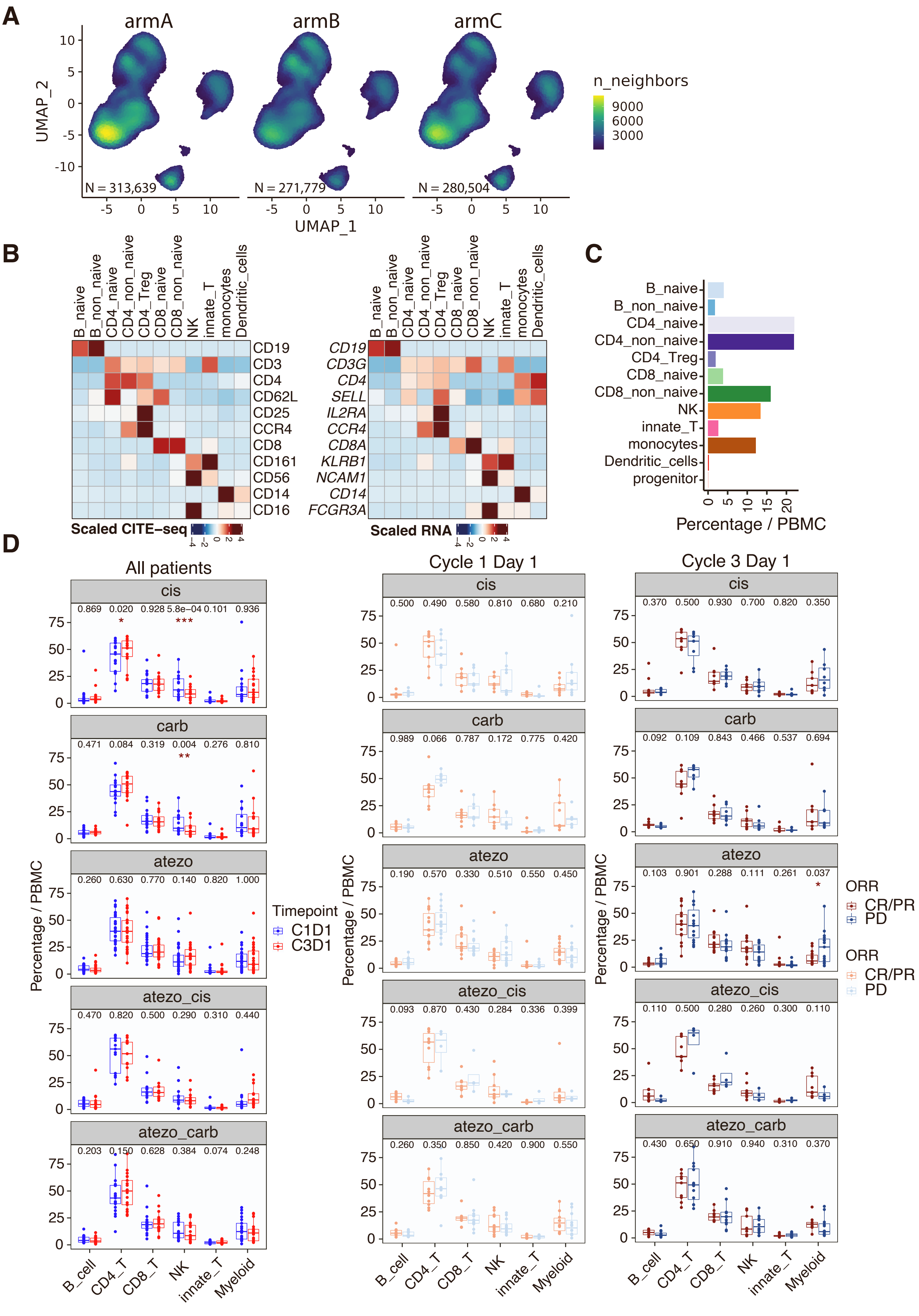
A
armA
armB
armC
UMAP_2
UMAP_1
N = 313,639
N = 271,779
N = 280,504
n_neighbors
9000
6000
3000
B
B_naive
B_non_naive
CD4_naive
CD4_non_naive
CD4_Treg
CD8_naive
CD8_non_naive
NK
innate_T
monocytes
Dendritic_cells
CD19
CD3
CD4
CD62L
CD25
CCR4
CD8
CD161
CD56
CD14
CD16
Scaled CITE−seq
CD19
CD3G
CD4
SELL
IL2RA
CCR4
CD8A
KLRB1
NCAM1
CD14
FCGR3A
Scaled RNA
C
B_naive
B_non_naive
CD4_naive
CD4_non_naive
CD4_Treg
CD8_naive
CD8_non_naive
NK
innate_T
monocytes
Dendritic_cells
progenitor
Percentage / PBMC
D
All patients
Cycle 1 Day 1
Cycle 3 Day 1
cis
0.869 0.020 0.928 5.8e−04 0.101 0.936
0.500 0.490 0.580 0.810 0.680 0.210
0.370 0.500 0.930 0.700 0.820 0.350
carb
0.471 0.084 0.319 0.004 0.276 0.810
0.989 0.066 0.787 0.172 0.775 0.420
0.092 0.109 0.843 0.466 0.537 0.694
atezo
0.260 0.630 0.770 0.140 0.820 1.000
0.190 0.570 0.330 0.510 0.550 0.450
0.103 0.901 0.288 0.111 0.261 0.037
atezo_cis
0.470 0.820 0.500 0.290 0.310 0.440
0.093 0.870 0.430 0.284 0.336 0.399
0.110 0.500 0.280 0.260 0.300 0.110
atezo_carb
0.203 0.150 0.628 0.384 0.074 0.248
0.260 0.350 0.850 0.420 0.900 0.550
0.430 0.650 0.910 0.940 0.310 0.370
Percentage / PBMC
Timepoint
C1D1
C3D1
ORR
CR/PR
PD
ORR
CR/PR
PD
B_cell
CD4_T
CD8_T
NK
innate_T
Myeloid

Figure S3. The proportions of major cell types in PBMCs remain unchanged after treatment. Related to Figure 3.
(A) UMAP embedding as shown in Figure 3B as a point-density plot split by cells from am A (N = 313,639), arm B (N = 271,779) and arm C (N = 280,504).
(B) Heatmaps showing scaled expression of the surface proteins as measured by CITE-seq (left) and the corresponding genes (right) as measured by single cell RNA-seq. The data indicated consistent expression between CITE-seq and single cell RNA-seq.
(C) Bar plot showing the abundance of different cell types in PBMCs.
(D) Box plot comparing the proportions of each cell type at baseline (C1D1) versus on-treatment (C3D1) in different treatment groups (left). Nominal P values derived from a two-tailed paired Student t-test are shown, and red asterisks represent significance (* $P < 0.05$; ** $P < 0.01$; *** $P < 0.001$). Box plots comparing the proportions of each cell type between responders (i.e., patients with complete response or partial response [CR/PR]) and nonresponders (i.e., patients with progressive disease [PD]) at baseline (C1D1, middle) and on-treatment (C3D1, right) in different treatment groups. Nominal P values derived from the two-tailed unpaired Student t-test are shown.

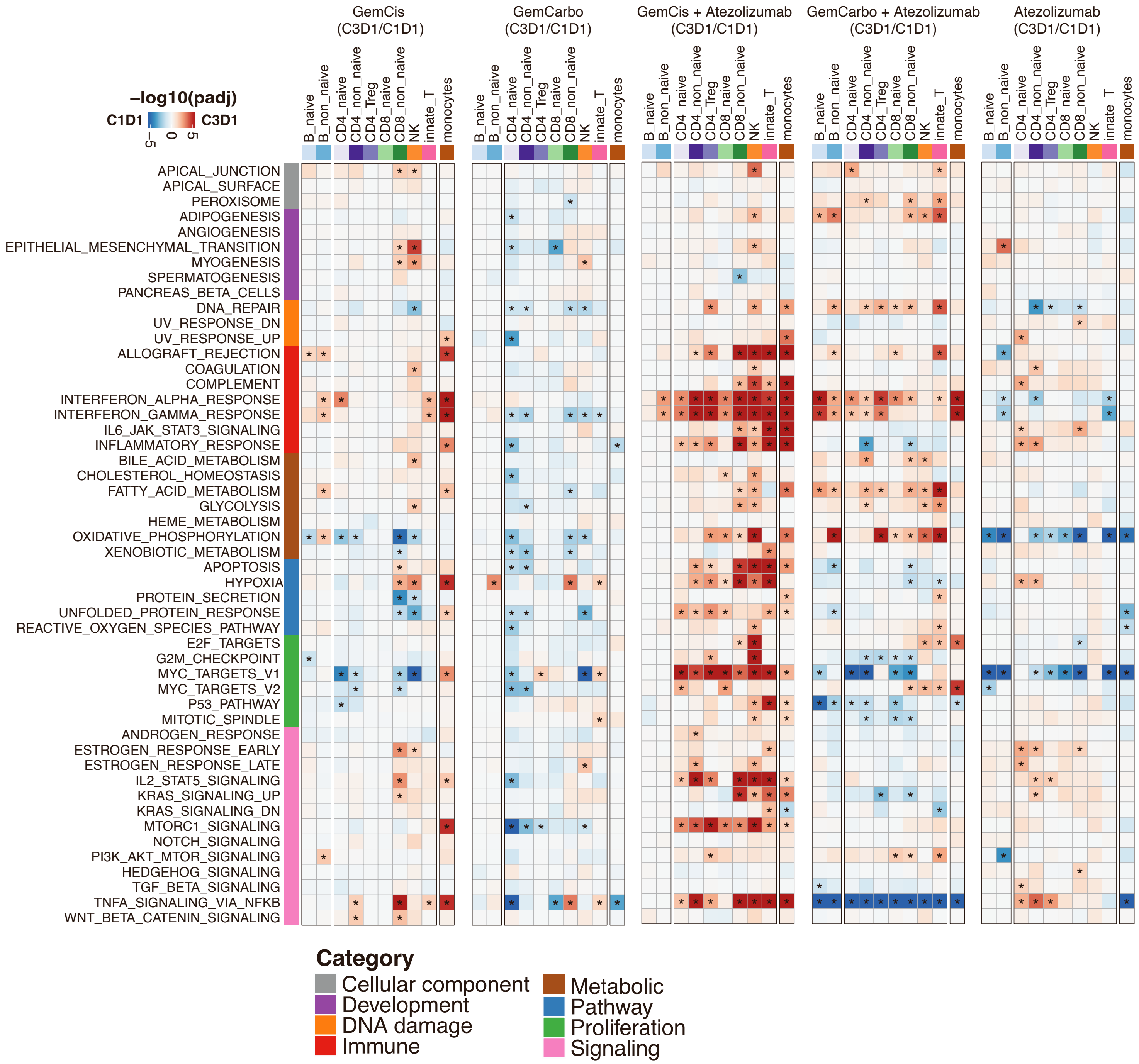

Figure S4. GemCis with and without atezolizumab versus GemCarbo with and without atezolizumab induces proinflammatory transcriptional programs across multiple immune cell subsets in PBMCs. Related to Figure 3.
Heatmaps comparing the pathway enrichment of all 50 Hallmark gene sets on-treatment (C3D1) versus baseline (C1D1) across multiple immune cell types in different treatment groups. Red indicates enrichment in on-treatment samples, and blue indicates enrichment at baseline. The hue represents the false-discovery rate (FDR) significance, derived from the fgsea package. Black asterisks represent FDR <0.05.

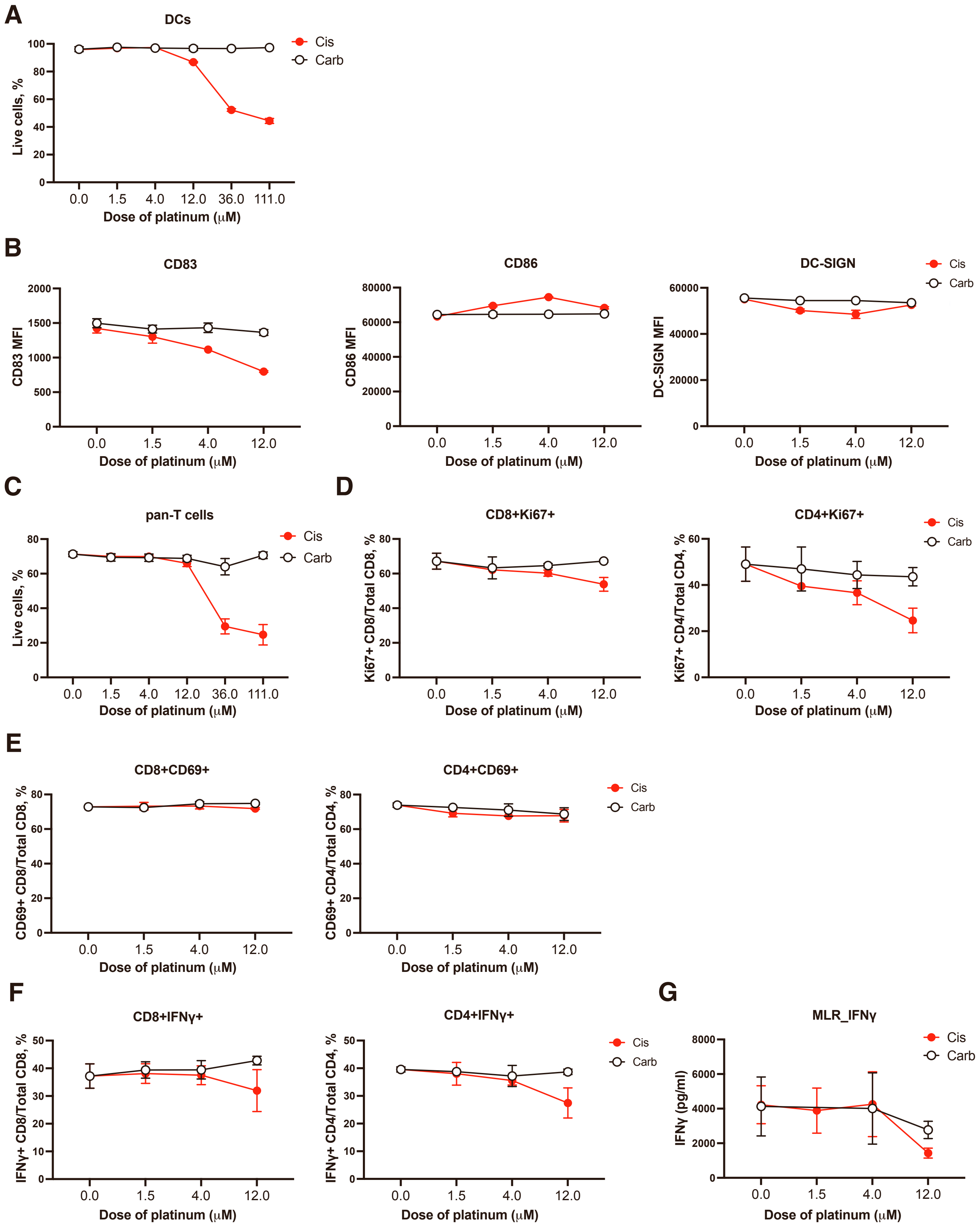

A
DCs
Live cells, %
100
80
60
40
20
0
0.0
1.5
4.0
12.0
36.0
111.0
Dose of platinum (μM)
Cis
Carb
B
CD83
CD83 MFI
2000
1500
1000
500
0
CD86
CD86 MFI
80000
60000
40000
20000
0
DC-SIGN
DC-SIGN MFI
60000
40000
20000
0
Dose of platinum (μM)
Cis
Carb
C
pan-T cells
Live cells, %
80
60
40
20
0
Dose of platinum (μM)
Cis
Carb
D
CD8+Ki67+
Ki67+ CD8/Total CD8, %
CD4+Ki67+
Ki67+ CD4/Total CD4, %
Dose of platinum (μM)
Cis
Carb
E
CD8+CD69+
CD69+ CD8/Total CD8, %
CD4+CD69+
CD69+ CD4/Total CD4, %
Dose of platinum (μM)
Cis
Carb
F
CD8+IFNγ+
IFNγ+ CD8/Total CD8, %
50
40
30
20
10
0
CD4+IFNγ+
IFNγ+ CD4/Total CD4, %
Dose of platinum (μM)
Cis
Carb
G
MLR_IFNγ
IFNγ (pg/ml)
8000
6000
4000
2000
0
Dose of platinum (μM)
Cis
Carb

Figure S5. Cisplatin and carboplatin at sub-cytotoxic levels do not have direct effects on the activation and function of DCs and T cells. Related to Figure 5.

(A) Average percentages of live DCs treated with increasing concentrations of cisplatin or carboplatin (n = 3).

(B) Representative plots of CD83, CD86, and DC-SIGN surface protein expression in DCs treated with increasing concentrations of cisplatin or carboplatin (n = 3).

(C) Average percentages of live pan-T cells with increasing concentrations of cisplatin or carboplatin (n = 3).

(D) Representative plots of the percentages of proliferating CD8 (left) and CD4 (right) T cells, as measured by Ki67, after treatment with increasing concentrations of cisplatin or carboplatin (n = 3).

(E) Representative plots of the percentages of activated CD8 (left) and CD4 (right) T cells, as measured by CD69, after treatment with increasing concentrations of cisplatin or carboplatin (n = 3).

(F) Representative plots of the percentages of IFNγ+ CD8 (left) and CD4 (right) T cells after treatment with increasing concentrations of cisplatin or carboplatin (n = 3).

(G) Representative plots of IFNγ secretion as measured in the supernatant from the co-culture of DC cells with pan-T cells treated with increasing concentrations of cisplatin or carboplatin (n = 4). In (A-G), error bars represent standard deviation.

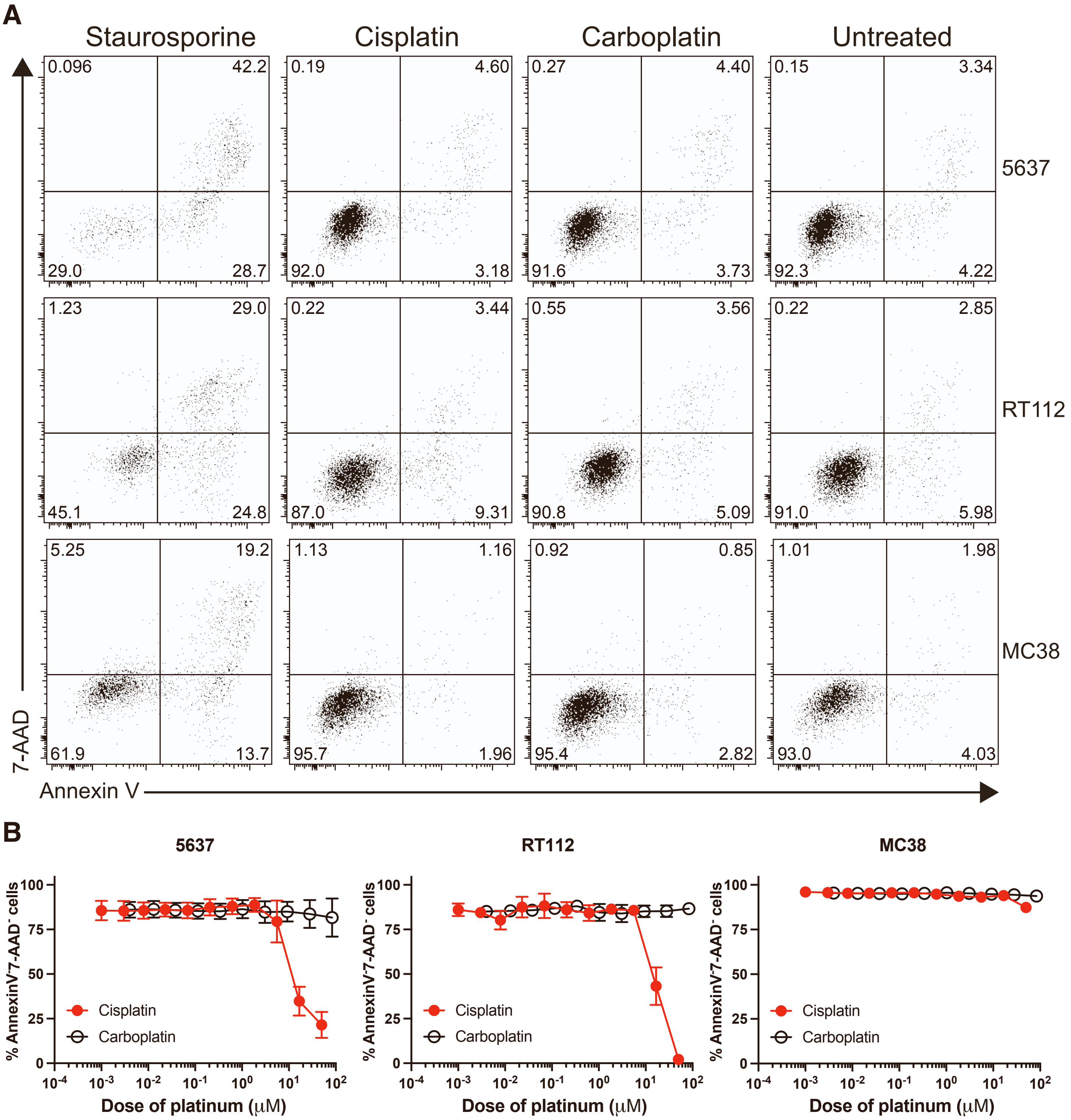

Figure S6. Cisplatin and carboplatin do not induce apoptosis within the range of clinically relevant concentrations (i.e., approximating peak serum concentrations achieved in humans at doses used to treat UC) in human urothelial bladder cancer (5637 and RT112) and murine colon adenocarcinoma (MC38) cell lines. Related to Figure 5.

(A) Representative flow cytogram showing tumor cell death as measured by 7-AAD and annexin V in 5637 (top), RT112 (middle), and MC38 (bottom) cell lines treated for 24 hours with staurosporine (125 nM, positive control for apoptosis induction), cisplatin (5 μM), or carboplatin (30 μM). Data depict one representative experiment of two independent experiments.

(B) Cells were treated with dose titrations of cisplatin or carboplatin. The proportions of non-apoptotic (annexin V–negative and 7-AAD–negative) cells were quantified at 24 hours posttreatment (n = 2). Data depict the aggregate of three independent experiments (mean ± SEM).

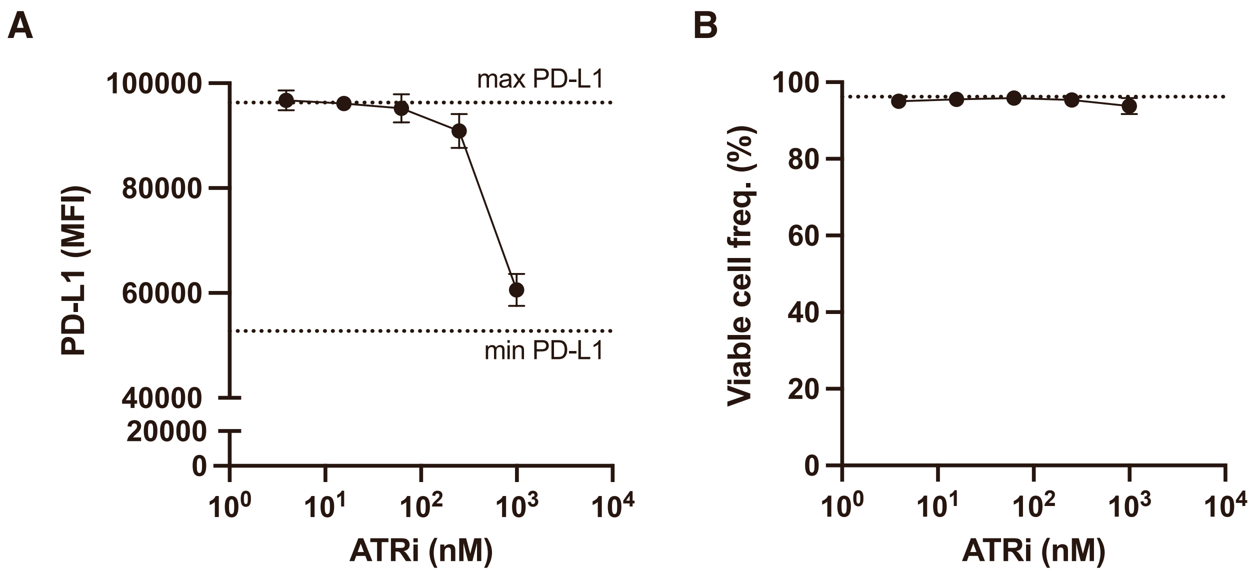

Figure S7. ATR inhibition by VE-821 is dose dependent and does not induce cell death. Related to Figure 5.
(A) Protein expression of PD-L1 as determined by median fluorescence intensity in the 5637 cells that were pre-treated with a dose titration of VE-821 followed by the addition of 5μM of cisplatin. Maximum and minimum dotted lines refer to PDL1 MFI in cisplatin-alone stimulated cells and untreated cells, respectively.
(B) Viability of the same cells as in (A), measured by 7-AAD dye incorporation. Dotted line refers to viability in untreated cells. Data depict the aggregate of two independent experiments (mean ± SEM).

A

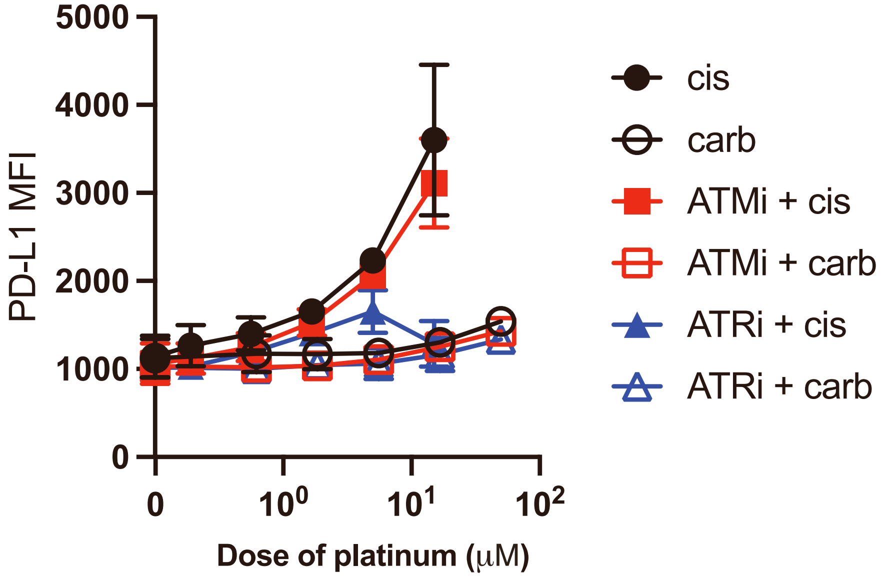

B

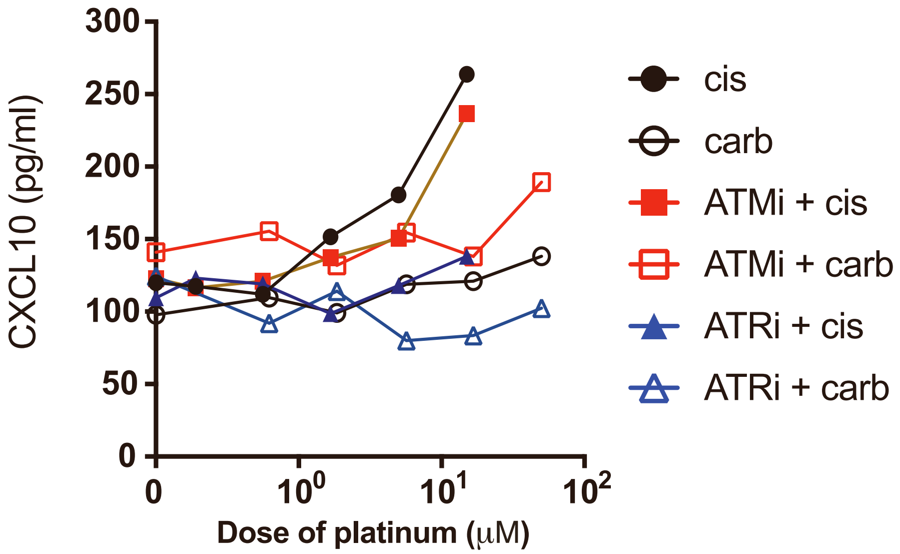

Figure S8. The direct induction of PD-L1 and CXCL10 in MC38 cells is mediated via the DNA damage transducer ATR. Related to Figure 5.

(A) Protein expression of PD-L1 as determined by median fluorescence intensity in the MC38 cell line treated with increasing concentrations of cisplatin or carboplatin for 24 hours, in the presence or absence of an ATM inhibitor (1 μM) and ATR inhibitor (1 μM) ($n = 3$). Data depict the aggregate of two independent experiments (mean ± SEM).

(B) CXCL10 (IP-10) secretion as measured by Mouse IP-10 ELISA in the supernatant collected from MC38 cells treated with increasing concentrations of cisplatin or carboplatin in the presence or absence of an ATM inhibitor (1 μM) and ATR inhibitor (1 μM). Supernatant was collected 24 hours after treatment. Data depict one experiment with duplicate conditions.

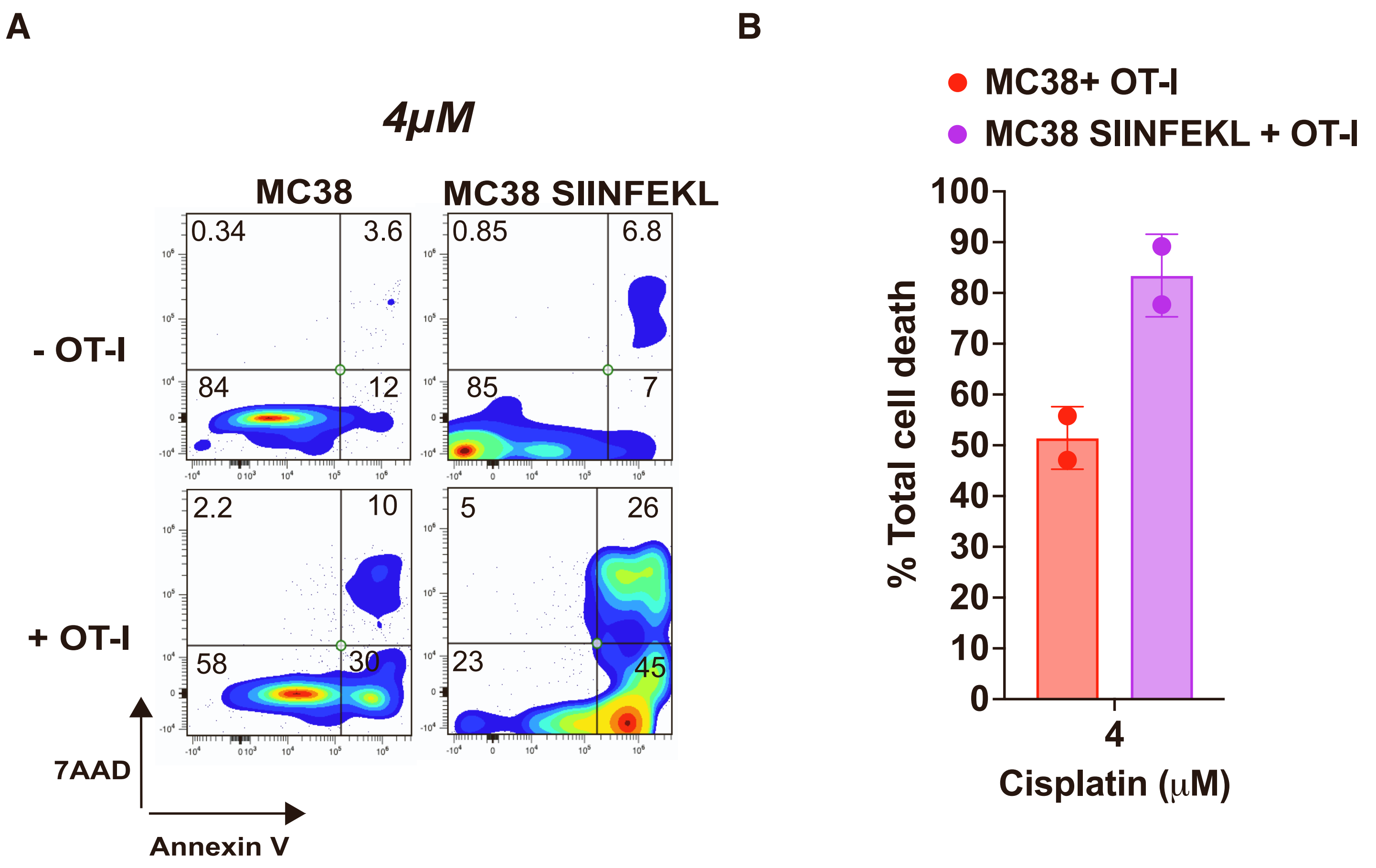

Figure S9. OT-I T-cell–mediated killing of MC38 tumor cells that express or do not express SIINFEKL OVA peptide. Related to Figure 6.

(A) Representative flow cytometry plots showing OT-I mediated cell killing of MC38 and MC38-SIINFEKL as measured by 7-AAD and annexin V staining within the tumor cells.

(B) The summarized percentage of OT-I T-cell–mediated tumor cell death. MC38 tumor cells were pretreated with or without 4uM of cisplatin for 24 hours, followed by co-culture with OT-I T cells for 5 hours before the assay. Data are mean ± SD.

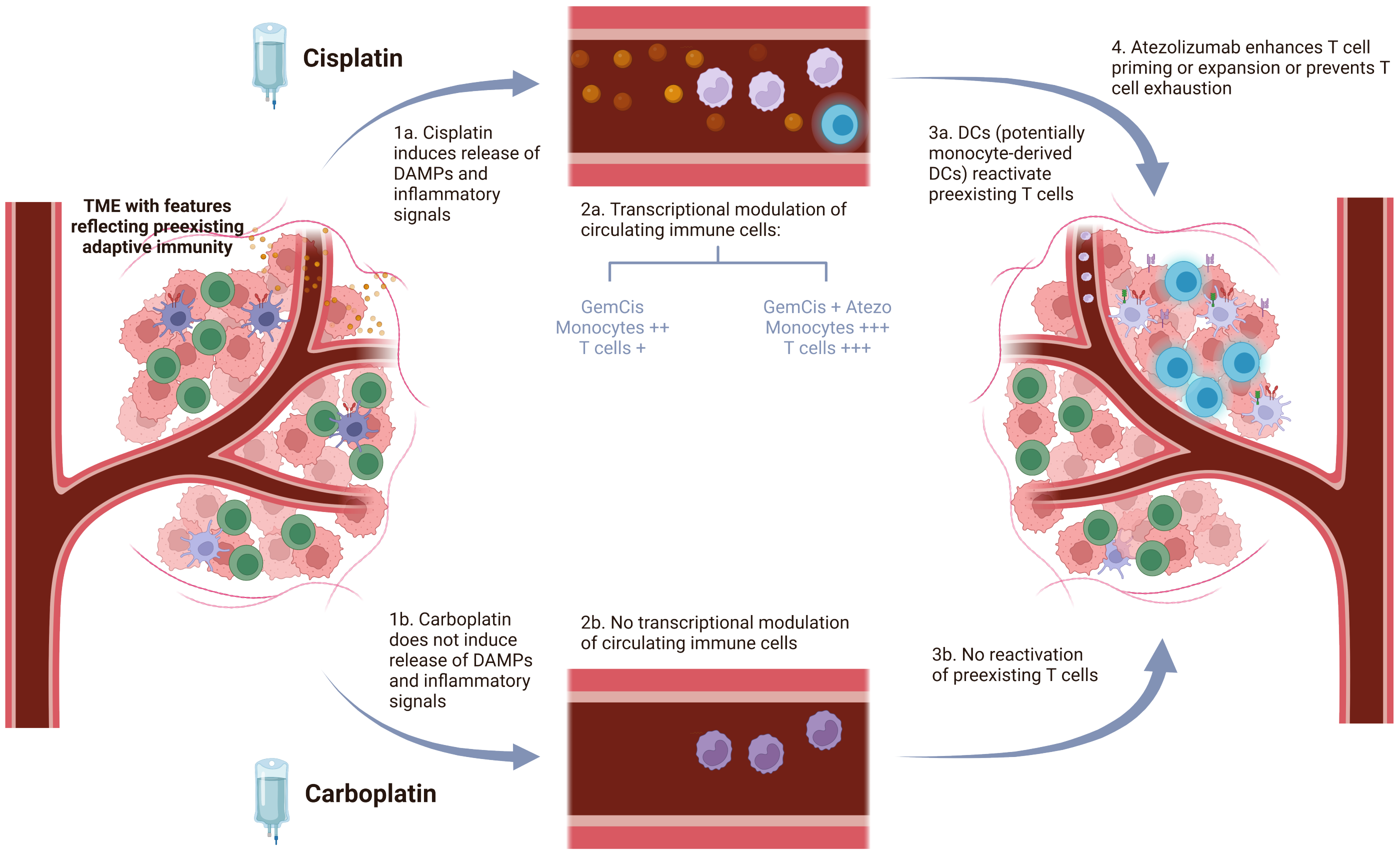

Figure S10. Proposed model for effects of cisplatin with and without atezolizumab on the cancer-immunity cycle. Related to Figures 1-6.

The model proposed involves direct modulation of cisplatin vs carboplatin on cancer cells (1), and secondary modulation on monocytes / DCs and T cells in the peripheral blood (2), ultimately leading to an enhanced immune niche favoring cancer cell killing (3), which is further enhanced by atezolizumab preventing T cell exhaustion (4). DAMP, danger-associated molecular pattern; DC, dendritic cell; GemCarbo, gemcitabine plus carboplatin; GemCis, gemcitabine plus cisplatin; TME, tumor microenvironment.
