# Supplementary material for: Immunomodulatory effects and improved outcomes with cisplatin- versus carboplatin-based chemotherapy plus atezolizumab in urothelial cancer
Source: Cell Rep Med. 2024 Jan 26;5(2):101393. doi: 10.1016/j.xcrm.2024.101393 (PMC10897541; doi:10.1016/j.xcrm.2024.101393)
Supplement: Document S2. Article plus supplemental information [file mmc2.pdf]

Cell Reports Medicine

Article

Immunomodulatory effects and improved outcomes with cisplatin- versus carboplatin-based chemotherapy plus atezolizumab in urothelial cancer

Graphical abstract

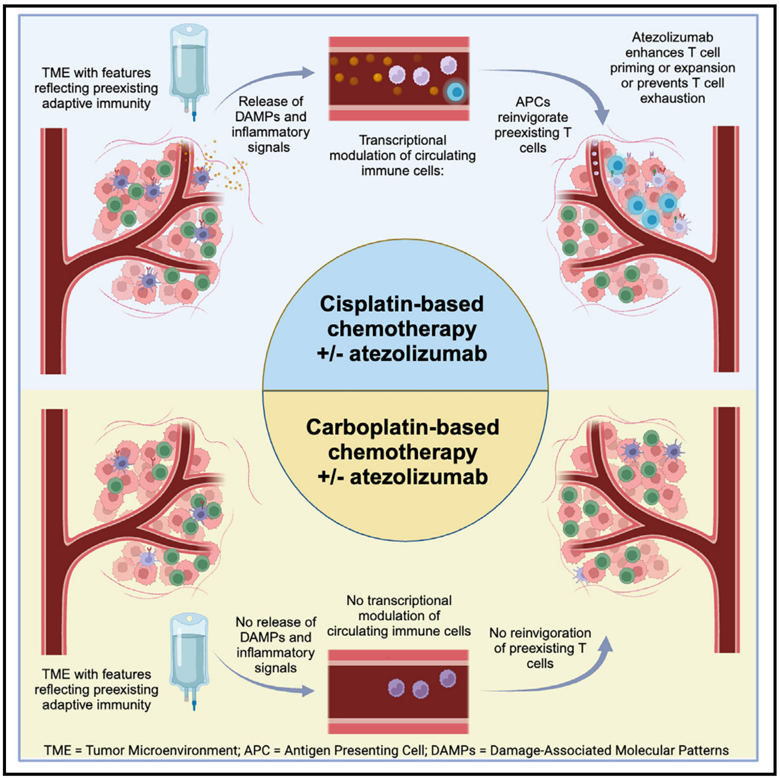

Highlights

- Patients with tumors showing preexisting adaptive immunity benefit more from cisplatin
- Cisplatin versus carboplatin modulates immune-related transcriptional programs
- Tumor cells primed by cisplatin versus carboplatin are sensitive to T cell killing

Authors

Matthew D. Galsky, Xiangnan Guan, Deepali Rishipathak, ..., Peter C. Black, Enrique Grande, Sanjeev Mariathasan

Correspondence

matthew.galsky@mssm.edu (M.D.G.), mariathasan.sanjeev@gene.com (S.M.)

In brief

Galsky et al. demonstrate that durable cancer control with cisplatin versus carboplatin is most prominent in patients with pretreatment tumors demonstrating features of restrained adaptive immunity. *In vitro*, they demonstrate that cisplatin versus carboplatin exerts direct immunomodulatory effects on cancer cells, promoting dendritic cell activation and antigen-specific T cell killing.

Galsky et al., 2024, Cell Reports Medicine *5*, 101393
February 20, 2024 © 2024 The Authors.
https://doi.org/10.1016/j.xcrm.2024.101393

CellPress

Cell Reports Medicine

CellPress
OPEN ACCESS

Article

Immunomodulatory effects and improved outcomes with cisplatin- versus carboplatin-based chemotherapy plus atezolizumab in urothelial cancer

Matthew D. Galsky,[1,*] Xiangnan Guan,[2,16] Deepali Rishipathak,[2,16] Aaron S. Rapaport,[2,16] Hesham M. Shehata,[2,16] Romain Banchereau,[2] Kobe Yuen,[2] Eugene Varfolomeev,[2] Ruozhen Hu,[2] Chia-Jung Han,[2] Haocheng Li,[3] Yuxin Liang,[2] Domagoj Vucic,[2] Li Wang,[4,5] Jun Zhu,[4,5] Haocheng Yu,[5] Rebecca H. Herbst,[6] Emma Hajaj,[6] Evgeny Kiner,[6] Aristotelis Bamias,[7] Maria De Santis,[8,9] Ian D. Davis,[10] José Ángel Arranz,[11] Eiji Kikuchi,[12] Sandrine Bernhard,[13] Patrick Williams,[2] Chooi Lee,[13] Ira Mellman,[2] Shomyseh Sanjabi,[2] Robert Johnston,[2] Peter C. Black,[14] Enrique Grande,[15] and Sanjeev Mariathasan[2,17,*]
[1]Division of Hematology and Medical Oncology, Tisch Cancer Institute, Icahn School of Medicine at Mount Sinai, New York, NY, USA
[2]Genentech Inc., South San Francisco, CA 94080, USA
[3]Hoffmann-La Roche Ltd, Mississauga, ON, Canada
[4]Department of Genetics and Genomic Sciences, Icahn School of Medicine at Mount Sinai, New York, NY, USA
[5]GeneDx, Stamford, CT, USA
[6]Immunai, New York, NY, USA
[7]National and Kapodistrian University of Athens, Athens, Greece
[8]Department of Urology, Charité – Universitätsmedizin, Berlin, Germany
[9]Department of Urology, Medical University of Vienna, Vienna, Austria
[10]Eastern Health Clinical School, Monash University, Melbourne, VIC, Australia
[11]Gregorio Marañón General University Hospital, Madrid, Spain
[12]St. Marianna University School of Medicine, Kawasaki, Japan
[13]Roche Products Ltd, Welwyn Garden City, UK
[14]Vancouver Prostate Centre, University of British Columbia, Vancouver, BC, Canada
[15]MD Anderson Cancer Center Madrid, Madrid, Spain
[16]These authors contributed equally
[17]Lead contact
*Correspondence: matthew.galsky@mssm.edu (M.D.G.), mariathasan.sanjeev@gene.com (S.M.)
https://doi.org/10.1016/j.xcrm.2024.101393

SUMMARY

In metastatic urothelial cancer (mUC), cisplatin versus carboplatin leads to durable disease control in a subset of patients. The IMvigor130 trial reveals more favorable effects with atezolizumab combined with gemcitabine and cisplatin (GemCis) versus gemcitabine and carboplatin (GemCarbo). This study investigates the immunomodulatory effects of cisplatin as a potential explanation for these observations. Our findings indicate that improved outcomes with GemCis versus GemCarbo are primarily observed in patients with pretreatment tumors exhibiting features of restrained adaptive immunity. In addition, GemCis versus GemCarbo ± atezolizumab induces transcriptional changes in circulating immune cells, including upregulation of antigen presentation and T cell activation programs. *In vitro* experiments demonstrate that cisplatin, compared with carboplatin, exerts direct immunomodulatory effects on cancer cells, promoting dendritic cell activation and antigen-specific T cell killing. These results underscore the key role of immune modulation in cisplatin's efficacy in mUC and highlight the importance of specific chemotherapy backbones in immunotherapy combination regimens.

INTRODUCTION

Platinum-based chemotherapy has been the mainstay of treatment for metastatic urothelial cancer (mUC) for decades. In a meta-analysis of randomized studies, cisplatin-based chemotherapy was found to be associated with higher overall and complete response rates than carboplatin-based chemotherapy in patients with mUC.[1] Moreover, unlike carboplatin, cisplatin has been linked to durable disease control in a subset of patients with mUC.[2,3] However, patients with mUC often have comorbidities, including renal dysfunction, that limit their ability to receive cisplatin, which is nephrotoxic.[4] Consequently, cisplatin may be considered unsuitable for approximately 30%–50% of patients with mUC, who instead typically receive carboplatin.[5] The mechanisms underlying the long-term survival achieved in some patients with mUC with cisplatin-based, but not carboplatin-based, chemotherapy remain poorly understood.[6]

This is an open access article under the CC BY license (http://creativecommons.org/licenses/by/4.0/).

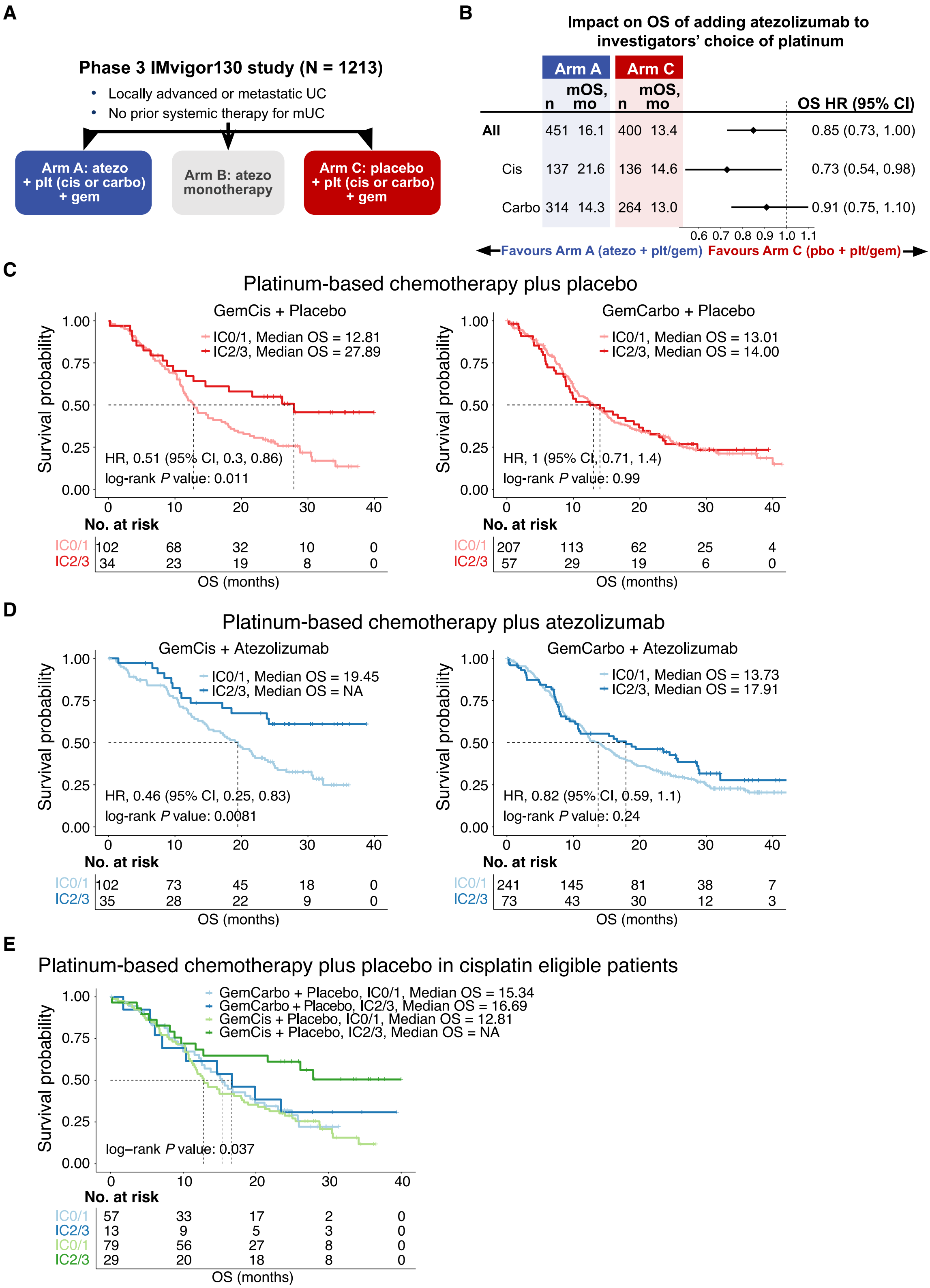

(legend on next page)

Programmed death-ligand 1/programmed death-1 (PD-L1/PD-1) immune checkpoint blockade has recently changed the treatment landscape for mUC.[7] Given favorable immunomodulatory effects of cytotoxic chemotherapy in model systems, the success seen with chemotherapy combined with immune checkpoint blockade in other advanced solid tumors, and the non-overlapping toxicities and drug resistance mechanisms of chemotherapy and immune checkpoint blockade,[8,9] the randomized phase 3 IMvigor130 trial (NCT02807636) was designed to test the efficacy of atezolizumab with or without platinum-based chemotherapy in mUC (Figure 1A).[10] For pragmatic reasons, platinum-based chemotherapy in IMvigor130 could have included either gemcitabine plus cisplatin (GemCis) or gemcitabine plus carboplatin (GemCarbo), at the investigators' discretion.[10] IMvigor130 demonstrated a significant improvement in the co-primary endpoint of investigator-assessed progression-free survival (PFS) with platinum-based chemotherapy plus atezolizumab versus platinum-based chemotherapy plus placebo, but the overall survival (OS) co-primary endpoint did not reach the prespecified efficacy boundary for statistical significance.[10] Intriguingly, subgroup analysis demonstrated a larger effect size on PFS when atezolizumab was added to GemCis (hazard ratio [HR] = 0.73; 95% CI, 0.55, 0.97) versus GemCarbo (HR = 0.84; 95% CI, 0.70, 1.02).[10]

IMvigor130 is not the only contemporary study to suggest that cisplatin- and carboplatin-based chemotherapy may have-different effects when employed with immune checkpoint blockade in mUC. KEYNOTE-361, which randomized patients with mUC to pembrolizumab, platinum-based chemotherapy, or the combination, also demonstrated a greater improvement in PFS on subset analysis when pembrolizumab was combined with GemCis (HR = 0.67; 95% CI, 0.51, 0.89) versus GemCarbo (HR = 0.86; 95% CI, 0.68, 1.09) over the respective platinum-based chemotherapy alone.[11] Multivariate Cox regression analysis for OS in the JAVELIN Bladder 100 study, which randomized patients with at least stable mUC after first-line platinum-based chemotherapy to maintenance avelumab versus best supportive care alone, revealed that initial cisplatin- versus carboplatin-based chemotherapy was independently associated with superior OS after controlling for known prognostic factors.[12]

The body of clinical data, coupled with prior research demonstrating the potential impact of cisplatin on antitumor immunity in model systems,[13,14] raised the hypothesis that immunomodulatory effects related to cisplatin may underlie the overall benefits observed with cisplatin versus carboplatin in mUC and the more favorable effect observed when combining GemCis versus GemCarbo with PD-1/PD-L1 blockade. To investigate these concepts and shed light on the potential importance of the choice of specific chemotherapy "backbones" in combination regimens with immune checkpoint blockade, we examined the immunomodulatory effects of cisplatin versus carboplatin employing biospecimens derived from participants enrolled on the IMvigor130 study as well as cellular systems.

RESULTS

Cisplatin ± atezolizumab was associated with improved survival outcomes

Among 451 participants in the atezolizumab plus platinum-based chemotherapy arm, 137 received GemCis and 314 received GemCarbo. Among 400 participants in the placebo plus platinum-based chemotherapy arm, 136 received GemCis and 264 received GemCarbo. Exploratory analyses (data cutoff: June 14, 2020) of the addition of atezolizumab versus placebo to chemotherapy indicated a larger effect on OS when atezolizumab was combined with GemCis (HR = 0.73; 95% CI, 0.54, 0.98) versus GemCarbo (HR = 0.91; 95% CI, 0.75, 1.10) (Figure 1B).

Prior studies have shown that immunogenic chemotherapeutic agents, such as anthracyclines, are more effective when the pretreatment tumor is infiltrated by T cells.[15] Therefore, we first probed the relationship between features of the pretreatment tumor microenvironment (TME) and outcomes with GemCis ± atezolizumab or GemCarbo ± atezolizumab in patients with mUC from IMvigor130. Participants whose baseline tumors harbored higher versus lower levels of PD-L1 expression on tumor-infiltrating immune cells (ICs) experienced longer OS when treated with GemCis (median OS = 27.89 versus 12.81 months) but not with GemCarbo (median OS = 14.00 versus 13.01 months; Figure 1C). The addition of atezolizumab appeared to further improve OS in participants with PD-L1 IC2/3 tumors treated with GemCis (median OS = not reached versus 27.89 months), whereas a less prominent effect was observed with GemCarbo (median OS = 17.91 versus 14.00 months) (Figures 1C and 1D). Similar results were observed using PFS as the outcome measure (Figure S1).

Although the relationship between PD-L1 expression and GemCis versus GemCarbo on OS was observed in two independent study arms (arms A and C), the use of GemCis versus

Figure 1. Survival outcomes with GemCis but not with GemCarbo are dependent on pretreatment tumor PD-L1 expression in an exploratory analysis of the IMvigor130 study

(A) Study design of phase 3 IMvigor130. Patients in arms A and C received atezolizumab and placebo, respectively, in combination with the investigators' choice of platinum drugs (cisplatin versus carboplatin) and gemcitabine. Arm A, n = 451; arm B, n = 362; arm C, n = 400.

(B) Forest plot showing overall survival in patients in arms A and C by use of GemCis versus GemCarbo. Hazard ratios, 95% confidence intervals, and p values were calculated using a univariate Cox model. The diamonds represent the hazard ratios, and the horizontal bars their 95% confidence intervals.

(C) Kaplan-Meier curves showing overall survival in patients in arm C stratified by PD-L1 status (IC2/3 versus IC0/1) and use of GemCis (left) or GemCarbo (right).

(D) Kaplan-Meier curves showing overall survival in patients in arm A stratified by PD-L1 status (IC2/3 versus IC0/1) and use of GemCis plus atezolizumab (left) or GemCarbo plus atezolizumab (right). In (C) and (D), p values were estimated using the log rank test. Hazard ratios and 95% confidence intervals were calculated using a univariate Cox model.

(E) Kaplan-Meier curves showing overall survival in patients in arm C stratified by actual receipt of GemCis or GemCarbo and tumor PD-L1 status (IC2/3 versus IC0/1). Patients classified as "cisplatin-eligible" according to standard criteria were included. p values were estimated using the log rank test. See also Figures S1 and S2 and Tables S1 and S2.

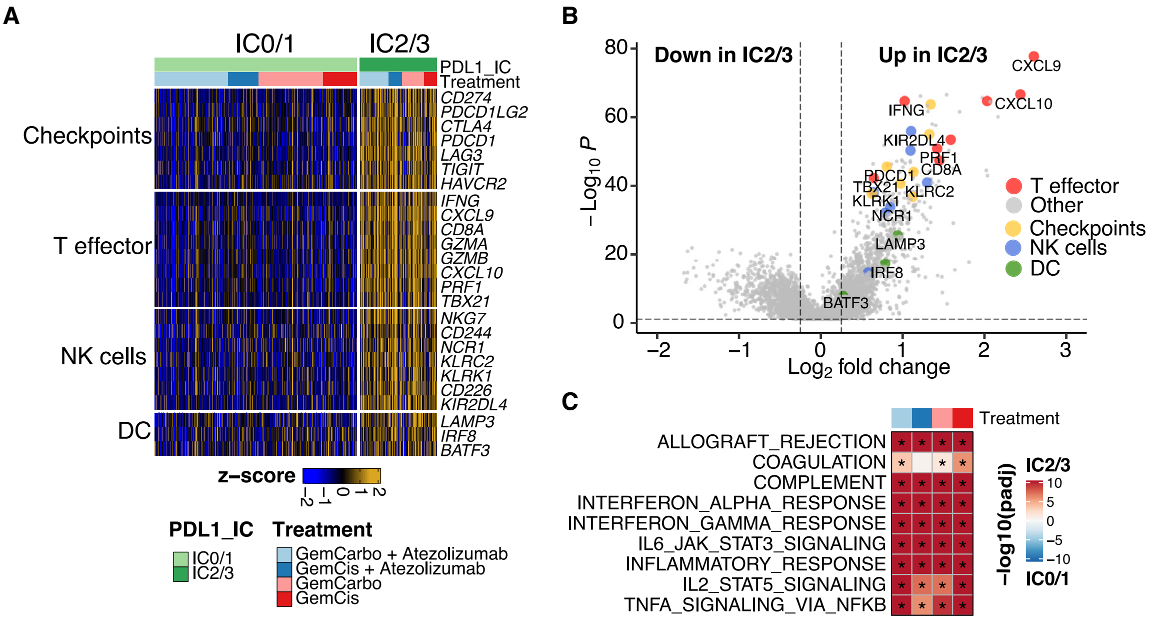

Figure 2. PD-L1 IC2/3 versus IC0/1 tumors are enriched in immune-related gene signatures

(A) Heatmap detailing immune-related gene signatures based on bulk RNA sequencing of pretreatment archival tumor specimens from the IMvigor130 study according to PD-L1 status (IC2/3 and IC0/1) and treatment arm.

(B) Volcano plot detailing differentially expressed genes based on bulk RNA sequencing of pretreatment archival tumor specimens from the IMvigor130 study according to PD-L1 status (IC2/3 versus IC0/1). Differential expression analysis was conducted with limma-based statistical methods and the Benjamini-Hochberg correction.

(C) Gene set enrichment analysis based on bulk RNA sequencing of pretreatment archival tumor specimens from the IMvigor130 study according to PD-L1 status (IC2/3 versus IC0/1) and treatment arm or specific platinum drug received. The hue represents the false discovery rate (FDR) significance derived from the fgsea package. Black asterisks represent FDR < 0.05.

GemCarbo was not assigned randomly in IMvigor130, raising the possibility that the differences in clinical outcomes were related to patient characteristics rather than the choice of platinum. To address this consideration, we took advantage of the fact that a subset of participants deemed "cisplatin-eligible" (i.e., not meeting standard criteria for cisplatin ineligibility[16]) received treatment with GemCarbo. Among these cisplatin-eligible participants in arm C (platinum-based chemotherapy plus placebo), favorable OS was observed in participants with PD-L1 IC2/3 versus IC0/1 tumors treated with GemCis but not with GemCarbo (Figure 1E). To further reinforce these findings, propensity scores for treatment with GemCis versus GemCarbo were calculated for participants in arm C according to age, Eastern Cooperative Oncology Group performance status, Bajorin risk factors,[6] and sites of metastatic disease. Among a 1:1 (GemCis:GemCarbo) propensity-score-matched subset of participants in arm C, a significant improvement in OS was observed with GemCis in participants with PD-L1 IC2/3 versus IC0/1 tumors, whereas there was no significant difference in OS in participants treated with GemCarbo according to PD-L1 status (Figure S2).

RNA sequencing of pretreatment tumors from participants enrolled in arms A and C revealed that PD-L1 IC2/3 versus IC0/1 tumors demonstrated enrichment in gene signatures, inferring the infiltration of T lymphocytes, natural killer (NK) cells, and dendritic cells (DCs) (Figure 2A). Furthermore, PD-L1 IC2/3 versus IC0/1 tumors demonstrated upregulation of key genes implicated in antitumor immunity, such as *CXCL9*, *CXCL10*, and *IFNG* (Figure 2B). The enrichment in immune-related gene signatures in PD-L1 IC2/3 versus PD-L1 IC0/1 pretreatment tumors were seen regardless of whether participants were subsequently treated with cisplatin or carboplatin (Figure 2C), suggesting that characteristics associated with choice of subsequent chemotherapy were not associated with different baseline TMEs. Therefore, features in the pretreatment TME suggestive of preexisting adaptive immunity[17,18] were associated with improved outcomes with GemCis but not GemCarbo in patients with mUC. These findings are reminiscent of prior work linking pretreatment immune cell infiltration with improved outcomes with "immunogenic chemotherapy"[15] and suggest that immunomodulatory effects may, at least in part, underlie durable disease control achieved in a subset of patients treated with GemCis, as well as the possible further improvement in OS with the addition of atezolizumab to GemCis.

Single-cell RNA sequencing of peripheral blood immune cells from baseline and on-treatment specimens

We postulated that the peripheral blood might provide a window into the immunomodulatory effects of GemCis versus GemCarbo with or without atezolizumab in the TME, possibly as a result of mediators being released from cancer or immune cells into the circulation.[19] We performed cellular indexing of transcriptomes and epitopes by sequencing (CITE-seq) on peripheral blood mononuclear cells (PBMCs) from participants

CellPress
OPEN ACCESS

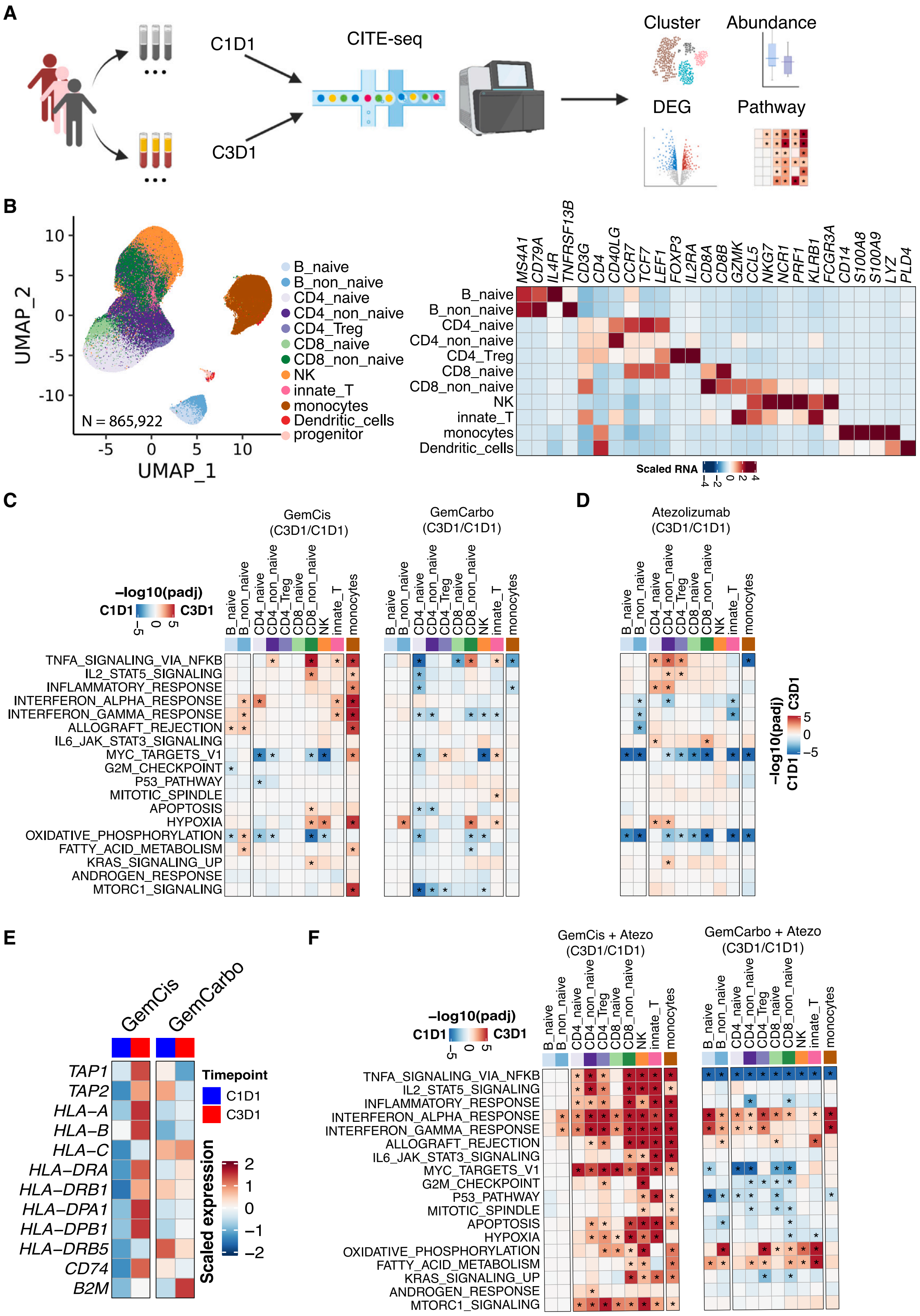

(legend on next page)

(n = 113) enrolled in IMvigor130 with available PBMCs obtained on cycle 1 day 1 (C1D1) and cycle 3 day 1 (C3D1) of treatment with GemCis or GemCarbo with or without atezolizumab, or atezolizumab alone, to profile RNA and surface proteins simultaneously at single-cell resolution (Figure 3A). The baseline characteristics of the CITE-seq cohort were well balanced across the study arms (Table S1) and similar to those of the broader IMvigor130 study population (Table S2). In total, 865,922 peripheral blood immune cells were profiled, and graph-based clustering revealed 12 major cell populations (Figures 3B and S3A–S3C). The quantity of most major cell populations did not change significantly while on treatment (from C1D1 to C3D1) or between responders and nonresponders across all study arms (Figure S3D).

Increased peripheral blood immune cell activation with cisplatin versus carboplatin

We initially focused on participants enrolled in arm C (platinum-based chemotherapy plus placebo). Gene set enrichment analysis compared differentially expressed genes in patients on treatment (C3D1 versus C1D1) with GemCis versus GemCarbo. GemCis but not GemCarbo led to a significant increase in the expression of several immune-related gene sets, including signatures related to nuclear factor-κB (NF-κB) signaling, interleukin-2 (IL-2) signaling, inflammatory response, and interferon (IFN) signaling, a finding that was most prominent in circulating monocytes (Figures 3C and S4). In contrast, significant on-treatment changes in the transcriptional program of circulating monocytes were not observed with atezolizumab alone (Figures 3D and S4).

Expression of antigen presentation machinery genes may be induced downstream of NF-κB or IFN signaling.[20] Indeed, antigen presentation machinery genes, including class I and II major histocompatibility complex (MHC) genes, were among the most upregulated in circulating monocytes on C3D1 versus C1D1 with GemCis treatment (Figure 3E). Together, these findings indicate that GemCis, but not GemCarbo, upregulates immune-related transcriptional programs in circulating immune cells (particularly in monocytes), including genes encoding proteins associated with antigen presentation and T cell priming.

Atezolizumab added to cisplatin leads to prominent modulation of T cell transcriptional programs

PD-L1 blockade may facilitate antitumor immunity by enhancing T cell priming and expansion or preventing exhaustion.[21] Therefore, we next sought to determine whether the addition of PD-L1 blockade to chemotherapy would increase the depth and breadth of transcriptional changes in circulating immune cells in participants enrolled in arm A. Atezolizumab combined with GemCis, versus with GemCarbo, led to a significant increase in the expression of a broader range of immune-related transcriptional programs, and across more diverse circulating immune cell subsets, compared with that observed with GemCis alone (Figures 3F and S4). Gene sets related to NF-κB, IL-2, and inflammatory responses remained among the most upregulated with GemCis plus atezolizumab versus GemCarbo plus atezolizumab. With the addition of atezolizumab to GemCis, these changes extended more conspicuously beyond monocytes to T and NK cells, and included increased expression of additional transcriptional programs associated with inflammation, metabolism, and the cell cycle (Figures 3F and S4).

Given the prominent effects of the addition of atezolizumab on the transcriptional program of circulating lymphocytes, we focused further attention on the 707,614 T and NK cells profiled in the study (Figure 4A). Specifically, we characterized the 171,729 CD8 T cells at high granularity given the central role of CD8 T cells in immune checkpoint blockade therapy. In total, nine CD8 T cell subsets were identified, seven of which included sufficient cells for downstream analysis (Figure 4B). We observed an increase in the expression of several gene signatures related to T cell activation among most CD8 T cell subsets, as well as CD4 T cell subsets, with GemCis plus atezolizumab but not with GemCarbo plus atezolizumab. This increase was also more pronounced compared with the effects of GemCis alone (Figure 4C). Intriguingly, while gene sets reflective of T cell activation on treatment were less prominent among participants with GemCis versus GemCis with atezolizumab, such changes in the former group appeared enriched in participants achieving an objective response to treatment with GemCis versus those with progressive disease as the best response (Figure 4D). Taken together, these results indicate that the addition of atezolizumab to GemCis, versus GemCarbo, leads to distinct transcriptional modulation of circulating immune cells including changes reflective of T cell activation states, possibly related to better T cell priming.

Cisplatin directly induces immune-related programs in cancer cells *in vitro*

While both cisplatin and carboplatin act by binding cellular DNA to form intrastrand, interstrand, and DNA-protein crosslinks,[22,23]

Figure 3. GemCis ± atezolizumab versus GemCarbo ± atezolizumab induces proinflammatory transcriptional programs across multiple immune cell subsets in PBMCs

(A) Single-cell CITE-seq experimental design and analysis workflow.

(B) Uniform Manifold Approximation and Projection (UMAP) visualization of single cells captured, colored by major cell types (n = 865,922) (left), and heatmap showing scaled expression of the canonical marker genes across cell types (right).

(C and D) Heatmaps showing pathway enrichment on-treatment (C3D1) versus at baseline (C1D1) across multiple immune cell types. Treatments received were GemCis (left, n = 17 pairs) or GemCarbo (right, n = 16 pairs) in arm C (C) and atezolizumab (n = 33 pairs) in arm B (D).

(E) Comparison of MHC class gene expression on-treatment (C3D1) versus at baseline (C1D1) in monocytes collected from patients receiving GemCis or GemCarbo in arm C.

(F) Heatmaps showing pathway enrichment on-treatment (C3D1) versus at baseline (C1D1) across multiple immune cell types. Treatments received were GemCis + atezolizumab (left, n = 14 pairs) or GemCarbo + atezolizumab (right, n = 24 pairs) in arm A. In (C), (D), and (F), red indicates enrichment in on-treatment samples and blue indicates enrichment at baseline. The hue represents the false discovery rate (FDR) significance derived from the fgsea package. Black asterisks represent FDR < 0.05. See also Figures S3 and S4.

CellPress
OPEN ACCESS

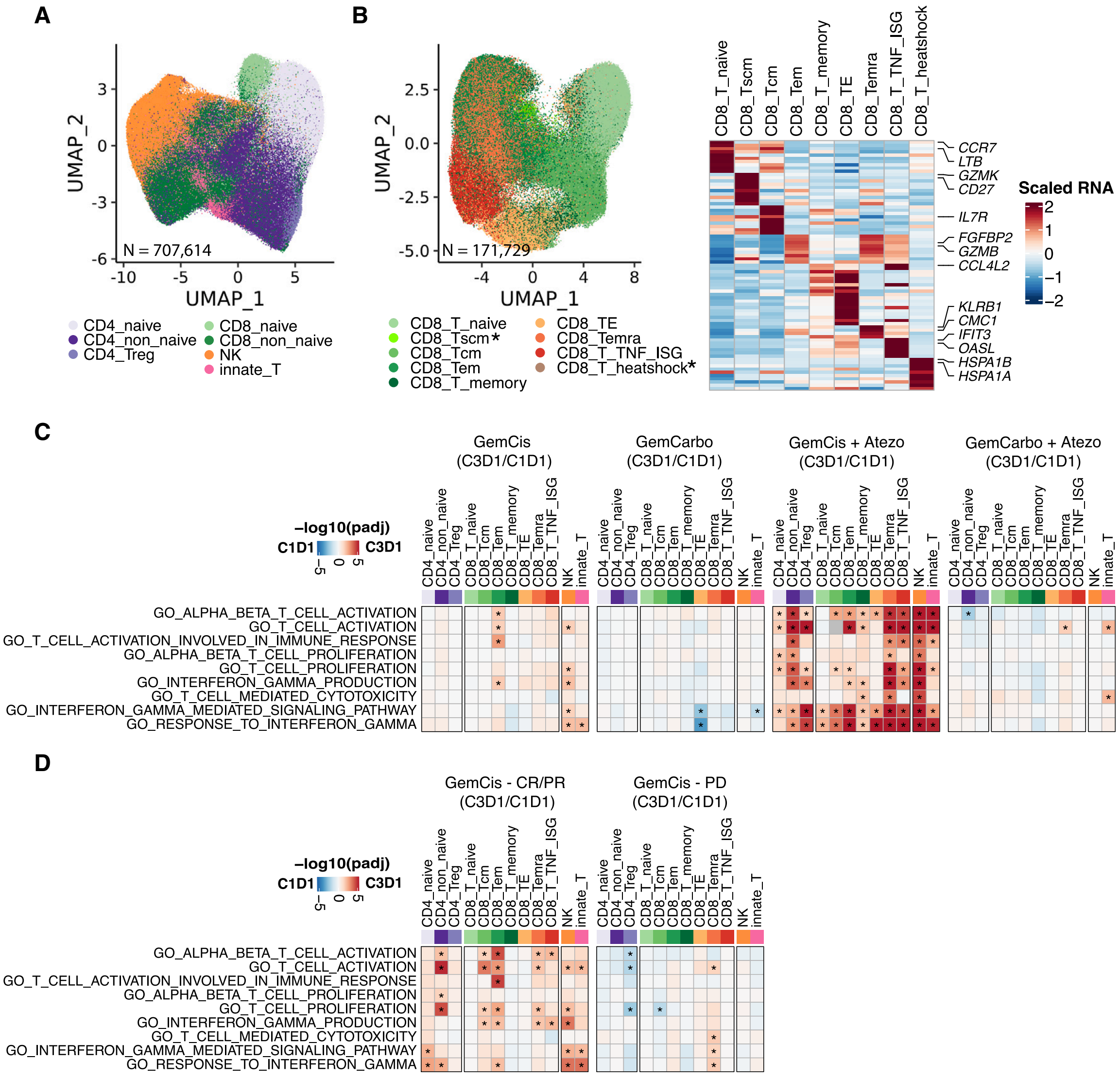

Figure 4. Atezolizumab added to GemCis versus GemCarbo leads to distinct transcriptional states of circulating immune cells including changes reflective of T cell activation states

(A) UMAP visualization of total T and NK cells captured, colored by major cell types (n = 707,614).

(B) UMAP visualization of total CD8 T cells captured, colored by different cell types (n = 171,729) (left), and heatmap showing scaled expression of the top 10 cell markers ranked by fold change in each cell type (right). Black asterisks indicate cell types not included in subsequent analysis due to low cell numbers.

(C) Heatmaps showing pathway enrichment on-treatment (C3D1) versus at baseline (C1D1) across T and NK cell types. Treatments received were GemCis (first panel, n = 17 pairs) or GemCarbo (second panel, n = 16 pairs) in arm C and GemCis + atezolizumab (third panel, n = 14 pairs) or GemCarbo + atezolizumab (fourth panel, n = 24 pairs) in arm A.

(D) Heatmaps showing pathway enrichment with GemCis on-treatment (C3D1) versus baseline (C1D1) across T and NK cell types in responders (left) and nonresponders (right). In (C) and (D), red indicates enrichment in on-treatment samples and blue indicates enrichment at baseline. The hue represents the false discovery rate (FDR) significance derived from the fgsea package. Black asterisks represent FDR < 0.05.

the two platinum compounds differ in their kinetics and potency in DNA adduct formation.[24] DNA damage interfaces tightly with innate and adaptive immunity through a complex network of DNA damage sensor, transducer, and effector proteins, leading to upregulation of several immune- and inflammation-related transcriptional programs.[13,14,25] Our observations from the IMvigor130 study suggested distinct immunomodulatory effects with GemCis versus GemCarbo, but several questions remained. (1) Were the transcriptional changes in circulating immune cells the direct result of chemotherapy on immune cells, or were they indirect effects secondary to the impact of chemotherapy on cancer cells in the tumor bed? (2) What events downstream of DNA damage induced by cisplatin versus carboplatin led to the observed systemic immune modulation? (3) Did the immunomodulatory effects associated with cisplatin versus carboplatin culminate in augmentation of antitumor immunity?

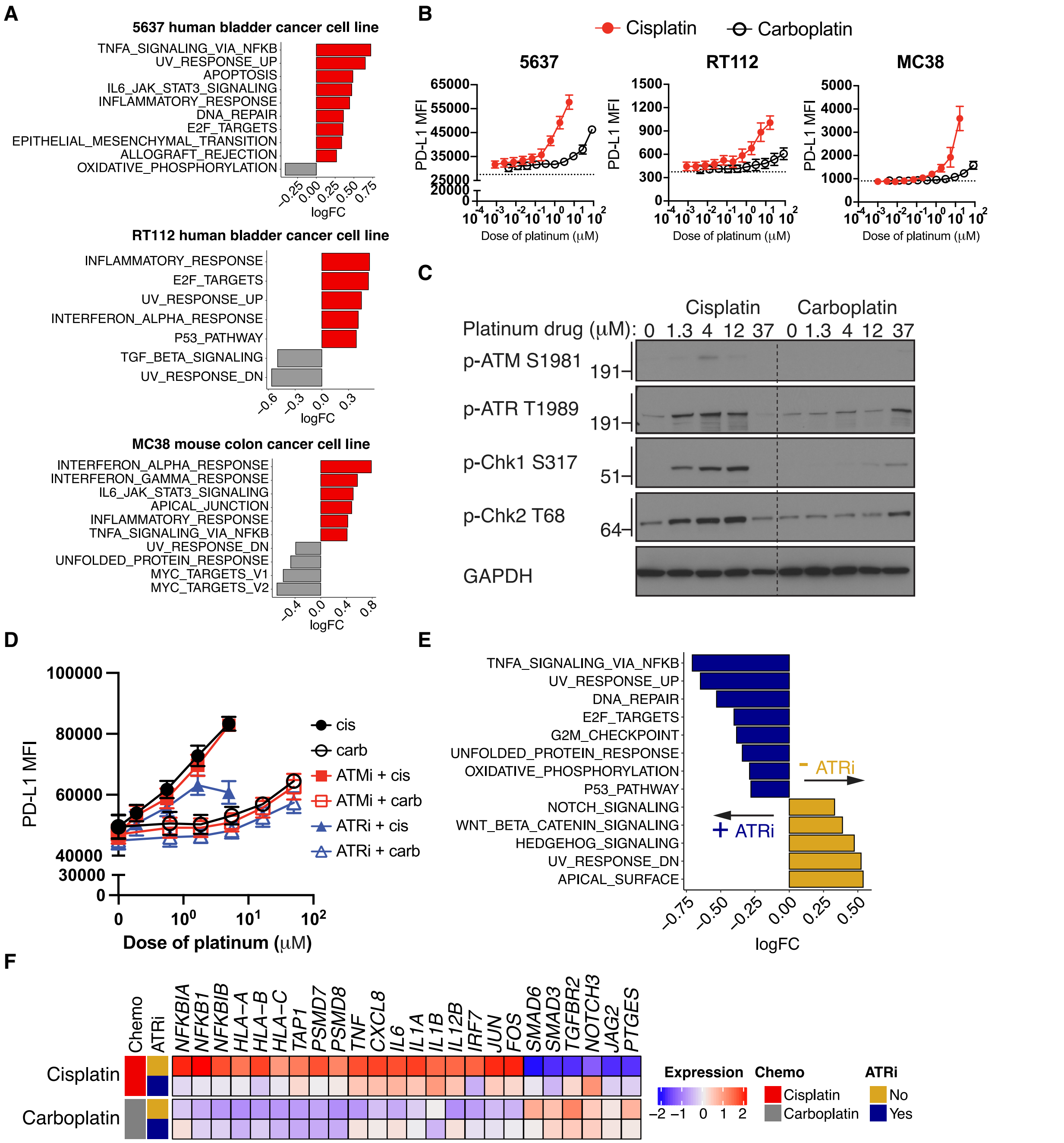

Figure 5. The direct effects of cisplatin versus carboplatin on downstream immune-related programs in cancer cells are mediated by the DNA damage transducer ATR

(A) Enrichment of Hallmark gene sets with cisplatin (red) versus carboplatin (gray) treatment in the 5637 human bladder cancer cell line (top), RT112 human bladder cancer cell line (middle), and MC38 murine colon cancer cell line (bottom). The cisplatin and carboplatin concentrations used were 5 and 35 μM, respectively, and cells were collected 24 h after treatment. Only pathways that were significantly changed with cisplatin versus carboplatin (false discovery rate [FDR] < 0.05) are shown. n = 3 per treatment group for the 5637 and RT112 cell lines; n = 2 per treatment group for the MC38 cell line.

(B) Protein expression of PD-L1 as determined by the median fluorescence intensity in the three cell lines treated with increasing concentrations of cisplatin (red) or carboplatin (black) for 24 h. Data depict the aggregate of three independent experiments (mean ± SEM).

(C) Representative immunoblots showing p-ATM S1981, p-ATR T1989, p-Chk1 S317, p-Chk2 T68, and GAPDH levels in lysates of the 5637 cell line treated with increasing concentrations of cisplatin or carboplatin for 6 h. One representative experiment out of four independent experiments is shown.

(D) Protein expression of PD-L1 as determined by median fluorescence intensity in the 5637 cell line treated with increasing concentrations of cisplatin or carboplatin for 24 h, in the presence or absence of an ATM inhibitor (KU-55933, 1 μM) or ATR inhibitor (VE-821, 1 μM). Data depict the aggregate of three independent experiments (mean ± SEM).

(legend continued on next page)

On-treatment tumor biopsies were not obtained in the Imvigor130 trial and therefore we turned our attention to cellular systems for further interrogation. Treatment of human PBMCs with increasing, but sub-cytotoxic, concentrations of cisplatin and carboplatin did not upregulate markers related to DC activation or T cell activation and proliferation, increase cytokine secretion, or potentiate mixed lymphocyte reaction responses (Figure S5), raising the possibility that the effects of cisplatin on immune cells may be mediated indirectly via effects on cancer cells. Indeed, treatment of 5637 and RT112 human bladder cancer cells and MC38 murine colon cancer cells—a commonly used cell line in cancer immunotherapy studies—with cisplatin or carboplatin at clinically relevant concentrations (i.e., 4 μM cisplatin and 37 μM carboplatin, corresponding approximately to the respective peak serum concentrations of cisplatin and carboplatin in humans achieved with conventional doses used to treat UC[26–28]) resulted in upregulation of immune- and inflammation-related gene sets with cisplatin versus carboplatin (Figures 5A and S6). Furthermore, a greater increase in the expression of PD-L1 protein was observed in tumor cells treated with cisplatin than with carboplatin (Figure 5B).

DNA damage transducer ATR mediates the immunomodulatory impact of cisplatin on cancer cells

To define the events proximal to DNA damage that might contribute to the immunomodulatory effects of cisplatin versus carboplatin, we treated 5637 bladder cancer cells with increasing concentrations of the platinum drugs and probed the impact on DNA damage transducers ATM and ATR. Cisplatin has previously been shown predominantly to induce an ATR-dependent DNA damage response.[29–31] We observed substantial activation of ATR and the downstream kinases Chk1 and Chk2 with increasing concentrations of cisplatin, but this was less apparent with carboplatin (Figure 5C). To reinforce the causal nature of these findings and link to downstream events, we treated 5637 and MC38 cells with increasing concentrations of cisplatin and carboplatin with or without small-molecule kinase inhibitors targeting ATR (VE-821) or ATM (KU-55933) and assessed the impact on surface PD-L1 protein expression (5637 and MC38) and CXCL10 secretion (MC38). ATR inhibition at doses that did not impact cell viability (Figure S7), but were consistent with doses previously shown to selectively inhibit ATR in cellular systems,[32] abrogated cisplatin-induced upregulation of surface PD-L1 expression or CXCL10 secretion (Figures 5D and S8). Similar results were not observed with ATM inhibition. We also treated 5637 cells with cisplatin and carboplatin, with or without ATR inhibition, and explored the resultant transcriptional changes. ATR inhibition mitigated cisplatin-induced modulation of key immune-related transcriptional programs observed at the level of gene signatures and individual genes including those encoding MHC class I molecules and inflammatory signals implicated in antigen-presenting cell activation (Figures 5E and 5F). These data support the hypothesis that direct effects of cisplatin versus carboplatin on cancer cells may, at least in part, underlie secondary transcriptional changes in immune cells.

Cisplatin-primed cancer cells enhance DC activation, T cell proliferation, and antigen-specific T cell-mediated tumor cell killing

Prior studies have shown that immunogenic chemotherapy "reboots" preexisting antigen-specific T cell responses within the TME through recruitment and activation of antigen-presenting cells derived from circulating monocytes.[33] Further research indicates that tumor-specific CD8$^+$ T cells only acquire the canonical effector program within the TME and that this process necessitates co-stimulation, which is most effectively provided by monocyte-derived DCs.[34] Together, these findings potentially reconcile clinical observations that pretreatment TMEs reflecting a preexisting adaptive immune response are associated with chemotherapy efficacy. To model the impact of cisplatin versus carboplatin on the interplay of cancer cells, antigen-presenting cells, and T cells, we employed co-culture systems involving MC38 cells, given that we had previously established that cisplatin versus carboplatin led to upregulation of immune-related transcriptional programs in MC38 cells (Figures 5A and S8) and could leverage MC38-OVA cells (i.e., MC38 cells engineered to express and present ovalbumin) to probe antigen-specific T cell immunity. Treatment of MC38 cells with increasing concentrations of cisplatin versus carboplatin resulted in increased release of the danger signal high-mobility group box 1 (HMGB1) (Figure 6A), which is known to bind TLR4 receptors and facilitate antigen-presenting cell activation.[35,36] Co-culture of cisplatin- versus carboplatin-pretreated MC38 cells with monocyte-derived DCs induced higher expression of MHC class I and PD-L1 as well as co-stimulatory proteins CD40 and CD86 on DCs (Figure 6B). We extended the co-culture system to include cisplatin- and carboplatin-pretreated MC38-OVA cells, DCs, and OT-I T cells (expressing a transgenic T cell receptor designed to recognize ovalbumin) and measured OT-I T cell proliferation. Cisplatin-pretreated MC38-OVA cells induced greater OT-I T cell proliferation than did carboplatin-pretreated MC38-OVA cells and T cell proliferation was further enhanced with the addition of DCs (Figure 6C). To determine whether cisplatin-pretreated MC38-OVA cells were more susceptible to OT-I T cell-mediated cellular cytotoxicity, we evaluated induction of early apoptosis and cell death in cisplatin- and carboplatin-pretreated cancer cells after co-culture with OT-I T cells. Again, compared with carboplatin pretreatment, cisplatin pretreatment led to increased OT-I CD8$^+$ T cell-specific killing of MC38-OVA cells (Figures 6D and 6E). The antigen-specific nature of such killing was confirmed using MC38 cells lacking

(E and F) Bulk RNA-seq analysis of 5637 cells treated with 5 μM cisplatin or 35 μM carboplatin, in the absence (gold) or presence of 1 μM ATR inhibitor (navy blue) for 24 h.

(E) Enrichment of Hallmark gene sets. n = 4 per treatment group.

(F) Heatmap displaying the scaled transcriptional expression of genes involved in immune-related transcriptional programs. Heatmap shows an average score of n = 4 individual samples per treatment group. See also Figures S5–S8.

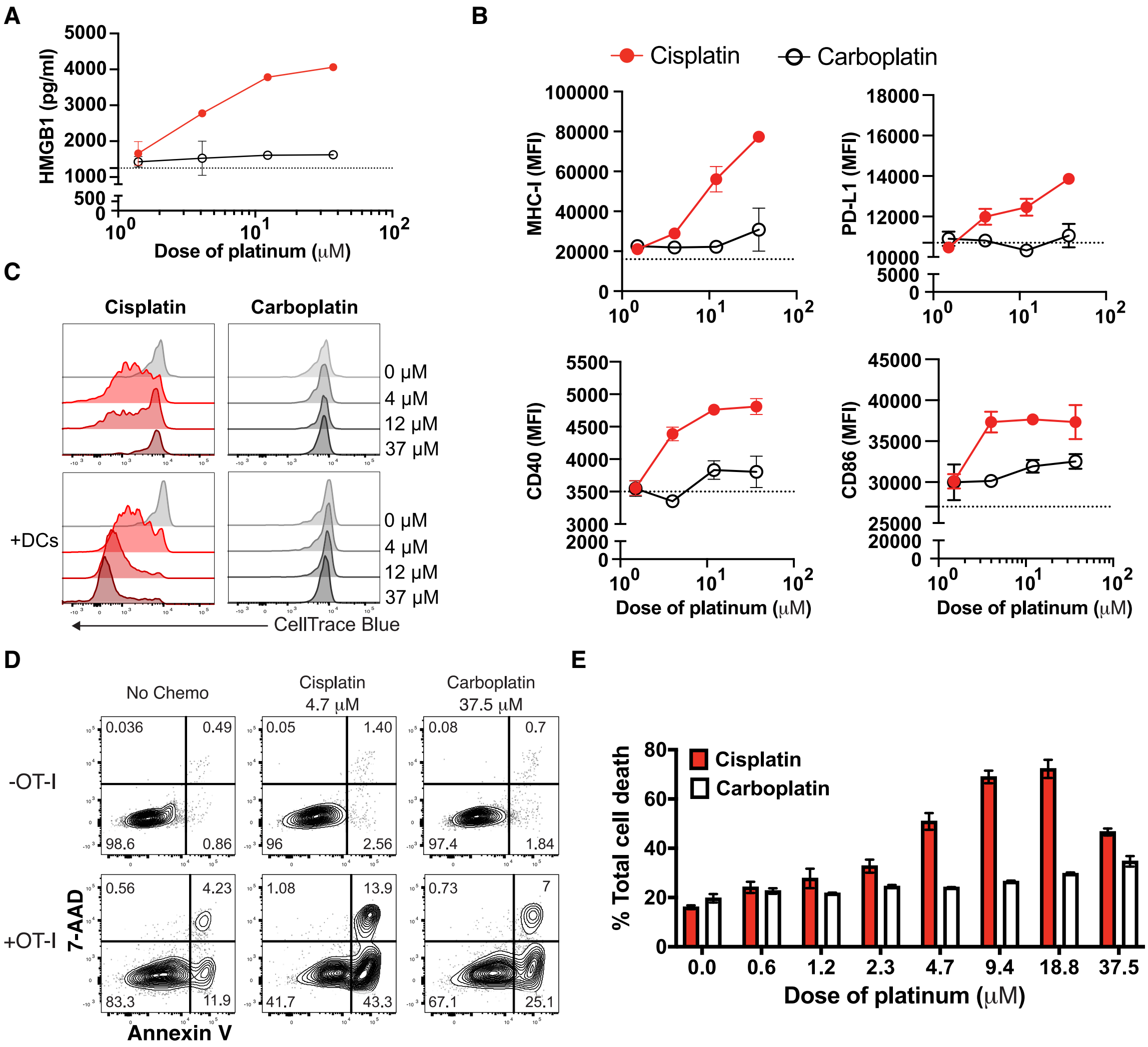

Figure 6. Co-culture of cisplatin- versus carboplatin-pretreated MC38 cancer cells with bone marrow-derived DCs and OT-I T cells induces DC activation, OT-I T cell proliferation, and antigen-specific T cell-mediated MC38 killing

(A) HMGB1 secretion as measured in the supernatant collected from MC38 cancer cells treated with increasing concentrations of cisplatin (red) or carboplatin (black). Supernatant was collected 3 days after treatment. Data depict one representative experiment of two independent experiments; duplicate conditions for each experiment. Data are mean ± SEM.

(B) Protein expression of MHC-I, PD-L1, CD40, and CD86 as measured by median fluorescence intensity in monocyte-derived DCs after 24 h of co-culture with MC38 cancer cells that were primed with dose titrations of cisplatin (red) or carboplatin (black) for 24 h. Data depict one representative experiment of three independent experiments; duplicate conditions for each experiment. Data are mean ± SEM.

(C) Flow cytometry histograms depicting OT-I T cell proliferation measured by CellTrace Blue dilution assay. MC38-OVA cells were pretreated with dose titrations of cisplatin or carboplatin for 24 h. OT-I T cells were co-cultured with pretreated MC38-OVA cells for 3 days in the presence or absence of monocyte-derived DCs before the proliferation assay. Data depict one representative experiment of two independent experiments.

(D and E) Representative flow cytogram showing OT-I T cell-mediated MC38-OVA cancer cell killing (D) and the summarized percentage of OT-I T cell-mediated tumor cell death (E) as measured by 7-AAD and annexin V staining. MC38-OVA tumor cells were pretreated with increasing concentrations of cisplatin or carboplatin for 24 h, followed by co-culture with OT-I T cells for 5 h before the assay. Data depict one representative experiment of three independent experiments; duplicate conditions for each experiment. Data are mean ± SEM. See also Figure S9.

OVA (Figure S9). Therefore, compared with carboplatin, cisplatin directly induced expression of immune-related transcriptional programs in cancer cells, and in co-culture, cisplatin- versus carboplatin-treated cancer cells enhanced activation of antigen-presenting cells, T cell proliferation, and antigen-specific tumor cell killing.

DISCUSSION

Cisplatin- versus carboplatin-based chemotherapy demonstrates higher objective response rates, and can induce durable disease control, in patients with mUC. Yet, the mechanisms underlying these observations remain poorly defined. Subset

CellPress
OPEN ACCESS

analysis of the IMvigor130 trial showed that adding atezolizumab to GemCis versus GemCarbo was associated with a more favorable impact on PFS and OS. Through a series of investigations involving biospecimens derived from participants in IMvigor130, as well as cellular systems, we demonstrated that: (1) favorable OS with GemCis versus GemCarbo with and without atezolizumab was driven by the subset of participants with pretreatment tumors exhibiting features reflecting preexisting adaptive immunity, (2) compared with GemCarbo, GemCis induced upregulation of immune and inflammatory transcriptional programs in circulating monocytes, including antigen presentation machinery genes, (3) the addition of atezolizumab to GemCis led to upregulation of T cell activation programs in circulating T cells, (4) cisplatin versus carboplatin treatment of cancer cells *in vitro* increased expression of genes encoding antigen presentation machinery and inflammatory signals (implicated in antigen-presenting cell activation), at least in part via the DNA damage transducer ATR, and (5) coculture of cisplatin- versus carboplatin-treated cancer cells enhanced DC activation and increased antigen-specific T cell killing. Our findings support a model (Figure S10) in which cisplatin triggers key events in the cancer-immunity cycle,[37] potentially reactivating preexisting T cell responses within the TME by recruiting and activating antigen-presenting cells and facilitating T cell co-stimulation while also possibly overcoming immune evasion related to cancer cell-intrinsic MHC class I downregulation.[38] By enhancing T cell priming and expansion, or preventing exhaustion,[21] atezolizumab may build upon these events culminating in more robust antitumor immunity (Figure S10).

Although cisplatin and carboplatin have similar mechanisms of action, they differ in chemical structure and resulting pharmacokinetic and pharmacodynamic properties. Both cisplatin and carboplatin act by binding cellular DNA to form DNA adducts.[22,23] However, the more rapid kinetics of DNA adduct formation with cisplatin versus carboplatin has been shown to impact antitumor activity.[24] Compared with cisplatin, the greater stability of carboplatin accounts for its lower reactivity with nucleophilic sites of DNA,[24] and carboplatin has been shown to induce approximately 20 times less DNA adduct formation per mole than cisplatin.[39] These distinct profiles of cisplatin and carboplatin may contribute to the observed differences in immunomodulatory effects.

There are several key strengths to our study. IMvigor130 is among the largest completed phase 3 studies exploring first-line treatment of mUC. This is among the largest analyses profiling the transcriptional modulation of PBMCs at single-cell resolution in the context of various anticancer therapies. There is a paucity of clinical trials randomizing participants to different chemotherapy backbones administered in combination with immune checkpoint blockade leaving the critically important question of whether specific chemotherapeutic agents combine differently with immune checkpoint blockade unanswered. Therefore, we leveraged a large clinical trial where, in the context of the same trial, both cisplatin- and carboplatin-based chemotherapies were administered.

Our data corroborate and extend the results of prior studies highlighting the potential immunomodulatory effects of cisplatin. The immunopotentiating effect of cisplatin was demonstrated in early trials of the combination of biochemotherapy (with IL-2 and IFN-α) for melanoma.[40–43] In studies predominantly employing model systems, cisplatin was previously shown to upregulate MHC class I expression in both cancer cells and antigen-presenting cells, release damage-associated molecular patterns, recruit effector cells, and enhance the lytic activity of cytotoxic effectors.[13,35,36,44,45] Cisplatin has been shown to increase expression of PD-L1 in patients with lung cancer treated with neoadjuvant chemotherapy, and the combination of cisplatin and PD-L1 blockade significantly decreased the growth of lung tumors in mice compared with cisplatin or PD-L1 blockade alone.[46]

Two large randomized trials, IMvigor130 and Keynote-361, did not demonstrate an improvement in OS with PD-1/PD-L1 blockade added to platinum-based chemotherapy (i.e., pooling patients receiving GemCis or GemCarbo). Therefore, the ultimate clinical validation of our data would be a clinical trial randomizing patients with mUC to GemCis versus GemCis plus PD-1/PD-L1 blockade. Indeed, after completing our analysis, the sole clinical trial addressing this specific question in mUC was reported. Checkmate-901 met its coprimary endpoints demonstrating an improvement in PFS and OS with GemCis plus nivolumab versus GemCis reinforcing the clinical implications of the potential immunomodulatory effects of cisplatin.[47]

Our findings may simultaneously advance an understanding of three distinct, but related, concepts: (1) how cisplatin- but not carboplatin-based chemotherapy achieves durable disease control in a subset of patients with mUC, (2) how patients with mUC deriving the most benefit from cisplatin-based chemotherapy could potentially be identified based on pretreatment features of the TME, and (3) how different cytotoxic chemotherapeutic backbones may combine differently with immune checkpoint blockade. Further investigations in each of these domains may help to advance immunotherapy for UC and other malignancies.

Limitations of the study

There are potential limitations to our study. Participants in IMvigor130 were not randomized to GemCis versus GemCarbo; however, several lines of evidence and prior research support a platinum-specific effect on clinical outcomes. Specifically, in our study, the impact of GemCis versus GemCarbo on OS was linked to a pretreatment TME reflecting a preexisting adaptive immune response and this finding was consistent across (1) two independent study arms (arms A and C), (2) an analysis restricted to “cisplatin-eligible” patients treated with GemCis versus GemCarbo in arm C, and (3) an analysis of a propensity-score-matched population of patients treated with GemCis versus GemCarbo in arm C. Propensity score matching may limit bias due to observed confounders but we cannot rule out an impact of unobserved confounders on outcomes. On-treatment tumor biopsies were not performed in participants enrolled in IMvigor130, precluding direct assessment of the effects of GemCis or GemCarbo with and without atezolizumab on the TME. While our data support a model in which the transcriptional programs induced in immune cells are

secondary to the direct effects of cisplatin on cancer cells, we cannot rule out that direct effects on immune cells may play a role, although we did not observe such effects in experiments exposing human PBMCs to cisplatin versus carboplatin. Cisplatin has been demonstrated to induce immunomodulatory effects via a variety of pathways[14] and, while our data suggest that these effects are downstream of ATR, elucidation of the full spectrum of pathways that culminate in the observed transcriptional modulation is beyond the scope of our analysis. Although gemcitabine has been associated with immunomodulatory effects in prior studies in patients and model systems,[48] it was administered at the same dose in all treatment arms of IMvigor130, leading to our focus on cisplatin versus carboplatin with and without atezolizumab. While carboplatin-based chemotherapy combined with immune checkpoint blockade has improved outcomes in other malignancies, there are well established differences in the activity of cisplatin- versus carboplatin-based chemotherapy across various malignancies, and our data highlight the complex interplay of tumor type, characteristics of pretreatment TME, and type of platinum drug.

STAR★METHODS

Detailed methods are provided in the online version of this paper and include the following:

- KEY RESOURCES TABLE
- RESOURCE AVAILABILITY
  - Lead contact
  - Materials availability
  - Data and code availability
- EXPERIMENTAL MODEL AND STUDY PARTICIPANT DETAILS
  - Clinical samples
  - Cell lines
  - Animal strains
- METHOD DETAILS
  - Analysis of IMvigor130 bulk RNA-seq
  - 10X genomics scRNA-seq library construction and sequencing
  - Preprocessing of scRNA-seq, CITE-seq, and VDJ data
  - Cluster analysis of peripheral blood immune cells
  - Pseudo-bulk differential gene expression analysis of PBMCs
  - *In vitro* drug treatment on tumor cell lines and flow cytometry characterization
  - Tumor cell line bulk RNA-seq processing
  - Analysis of tumor cell line bulk RNA-seq
  - Immunoblotting
  - *In vitro* human T cell activation and drug treatment
  - *In vitro* human DC differentiation and mixed lymphocyte reaction (MLR) assay
  - Co-culture of murine DCs and MC38 cells
  - OT-I T cell proliferation assay
  - T-cell–mediated cytotoxicity assays
  - ELISA
- QUANTIFICATION AND STATISTICAL ANALYSIS
- ADDITIONAL RESOURCES

SUPPLEMENTAL INFORMATION

Supplemental information can be found online at https://doi.org/10.1016/j.xcrm.2024.101393.

ACKNOWLEDGMENTS

This work was supported by CA196521 (to M.D.G). This study was sponsored by F. Hoffmann-La Roche Ltd. Medical writing assistance for this manuscript was provided by Qing Yun Chong, PhD, of MediTech Media Ltd, and funded by F. Hoffmann-La Roche Ltd.

AUTHOR CONTRIBUTIONS

M.D.G., D.R., A.S.R., H.M.S., D.V., A.B., M.D.S., I.D.D., P.W., C.L., I.M., S.S., R.J., R.B., J.A.A., and S.M. conceived and designed the study. M.D.G., X.G., D.R., A.S.R., H.M.S., H.L., K.Y., E.V., R.H., C.-J.H., H.Y., R.H.H., E.H., E. Kiner, E. Kikuchi, P.W., C.L., I.M., R.J., P.C.B., E.G., R.B., L.W., J.Z., and S.M. analyzed and interpreted the data. M.D.G., D.R., Y.L., I.D.D., P.C.B., and S.M. provided study materials or patients. M.D.G., X.G., D.R., A.S.R., H.M.S., E.V., R.H., Y.L., D.V., E. Kiner, A.B., S.B., C.L., and P.C.B. collected and assembled the data. All authors wrote or contributed to review and revisions of the manuscript. M.D.G., D.R., K.Y., C.-J.H., H.L., D.V., M.D.S., I.D.D., E. Kikuchi, S.B., P.W., C.L., S.S., P.C.B., E.G., R.B., L.W., J.Z., J.A.A., and S.M. approved the final manuscript.

DECLARATION OF INTERESTS

M.D.G. has received grants or contracts from Bristol Myers Squibb, Novartis, Dendreon, AstraZeneca, and Merck; and has received consulting fees from Bristol Myers Squibb, Merck, Genentech Inc., AstraZeneca, Pfizer, EMD Serono, Seagen, Janssen, Numab, Dragonfly, GlaxoSmithKline, Basilea, UroGen, RapptaTherapeutics, Alligator, Silverback, Fujifilm, and Curis.

X.G., D.R., A.S.R., H.M.S., R.B., K.Y., E.V., R.H., C.-J.H., Y.L., D.V., P.W., I.M., S.S., R.J., and S.M. are employees of Genentech Inc., and hold stocks or stock options in Roche/Genentech Inc. H.L. is an employee of Hoffmann-La Roche Ltd, Canada.

L.W., J.Z., and H.Y. are employees of Sema4.

E.H. was employed at Immunai during study conduct.

R.H.H. and E. Kiner are employees of Immunai.

A.B. has served as a consultant or advisor to Roche, Bristol Myers Squibb, MSD, and Pfizer; has received honoraria from Bristol Myers Squibb, MSD, and Pfizer; and has received educational and research grants from Pfizer, Pierre-Fabre, and Bristol Myers Squibb.

M.D.S. has received consulting fees from AAA, Accord, Amgen, Astellas, AstraZeneca, Basilea, Bayer, Bioclin, Bristol Myers Squibb, Eisai, Ferring, Immunomedics, Ipsen, Janssen, MSD, Merck, Novartis, Pfizer, Pierre Fabre Oncology, Roche, Sandoz, Sanofi, and Seagen; has received payment or honoraria for lectures, presentations, speakers bureaus, manuscript writing, or educational events from AAA, Accord, Amgen, Astellas, AstraZeneca, Basilea, Bayer, Bioclin, Bristol Myers Squibb, Eisai, Ferring, Immunomedics, Ipsen, Janssen, MSD, Merck, Novartis, Pfizer, Pierre Fabre Oncology, Roche, Sandoz, Sanofi, and Seagen; has received support for attending meetings and/or travel from AAA, Accord, Amgen, Astellas, AstraZeneca, Basilea, Bayer, Bioclin, Bristol Myers Squibb, Eisai, Ferring, Immunomedics, Ipsen, Janssen, MSD, Merck, Novartis, Pfizer, Pierre Fabre Oncology, Roche, Sandoz, Sanofi, and Seagen; has participated in a Data Safety Monitoring Board or Advisory Board for Roche, Orion, and CR-UK; and has other financial or non-financial interests by having worked on the ESMO guidelines on bladder cancer and the German S3 Leitlinie Blasenkarzinom.

I.D.D. has participated in a Data Safety Monitoring Board or Advisory Board for Roche/Genentech Inc. (WO30070 [IMvigor130] international steering committee; unpaid) and Merck/Pfizer (APAC GU Advisory Board; unpaid: honoraria are invoiced by and paid directly to ANZUP Cancer Trials Group); and has a leadership or fiduciary role in the ANZUP Cancer Trials Group (as a Director and Board Chair; unpaid).

J.A.A. has received third-party service as collaboration in the selection, and management of administrative requirements for the participation of SOGUG centers in the study from SOGUG during the conduct of the study; speaking or consulting fees from Bristol Myers Squibb, MSD, Roche, Astellas, Janssen Cilag, Pfizer, Novartis, and Bayer outside the submitted work; travel support from Bristol Myers Squibb, MSD, Roche, and Janssen Cilag outside the submitted work; research funding (from SOGUG) outside the submitted work from Bristol Myers Squibb, Novartis, and Pierre Fabre; and has participated in industry-sponsored clinical trials for Bristol Myers Squibb, MSD, Roche, Astellas, Janssen Cilag, Pfizer, and Novartis outside the submitted work.

E. Kikuchi has received grants or contracts (through his institution) from Takeda, Nippon Kayaku, and Taiho; has received consulting fees from Chugai, Nippon Kayaku, MSD, Takeda, Pfizer, Astellas, Bristol Myers Squibb, and Yansen; has received payment or honoraria for lectures, presentations, speakers bureaus, manuscript writing, or educational events from Takeda, Nippon Kayaku, and Taiho; and has received consulting fees from Chugai, Nippon Kayaku, MSD, Takeda, Taiho, AstraZeneca, Pfizer, Astellas, Bristol Myers Squibb, and Yansen.

S.B. is an employee of Roche Products Ltd, UK, holds stocks or stock options in Roche, and has other financial or non-financial interests in Roche.

C.L. is an employee of Roche Products Ltd, UK, holds stocks or stock options in Roche, and has other financial or non-financial interests in Roche.

P.C.B. has participated in advisory boards for Janssen, Merck, Roche/Genentech Inc., Bristol Myers Squibb, Urogen, EMD Serono, Bayer, Astellas, AbbVie, AstraZeneca, Ferring, H3-Biomedicine, Sanofi, Pfizer, Prokarium, Protara Therapeutics, Stimit, and Verity; has received payment or honoraria as a speaker from Janssen, Bayer, Ferring, H3-Biomedicine, and Pfizer; has participated in clinical trials for Roche/Genentech Inc., Bristol Myers Squibb, and AstraZeneca; and has received non-financial support (scientific collaboration on bladder cancer genomics/transcriptomics) from Decipher Biosciences/Veracyte.

E.G. has received grants or contracts from Astellas, AstraZeneca, Ipsen, Merck KGaA, and Pfizer; has received payment or honoraria for lectures, presentations, speakers bureaus, manuscript writing, or educational events from Astellas, AstraZeneca, Bayer, Bristol Myers Squibb, Eisai, Esteve, Ipsen, Janssen, Lilly, Merck KGaA, MSD, Pfizer, Raffo, and Roche; and has received support for attending meetings and/or travel from Roche, AstraZeneca, Bristol Myers Squibb, Pfizer, and Merck KGaA.

Received: December 8, 2022
Revised: March 9, 2023
Accepted: January 3, 2024
Published: January 26, 2024

REFERENCES

1. Galsky, M.D., Chen, G.J., Oh, W.K., Bellmunt, J., Roth, B.J., Petrioli, R., Dogliotti, L., Dreicer, R., and Sonpavde, G. (2012). Comparative effectiveness of cisplatin-based and carboplatin-based chemotherapy for treatment of advanced urothelial carcinoma. Ann. Oncol. *23*, 406–410.
2. De Santis, M., Bellmunt, J., Mead, G., Kerst, J.M., Leahy, M., Maroto, P., Gil, T., Marreaud, S., Daugaard, G., Skoneczna, I., et al. (2012). Randomized phase II/III trial assessing gemcitabine/carboplatin and methotrexate/carboplatin/vinblastine in patients with advanced urothelial cancer who are unfit for cisplatin-based chemotherapy: EORTC study 30986. J. Clin. Oncol. *30*, 191–199.
3. Sternberg, C.N., de Mulder, P., Schornagel, J.H., Theodore, C., Fossa, S.D., van Oosterom, A.T., Witjes, J.A., Spina, M., van Groeningen, C.J., Duclos, B., et al. (2006). Seven year update of an EORTC phase III trial of high-dose intensity M-VAC chemotherapy and G-CSF versus classic M-VAC in advanced urothelial tract tumours. Eur. J. Cancer *42*, 50–54.
4. Dash, A., Galsky, M.D., Vickers, A.J., Serio, A.M., Koppie, T.M., Dalbagni, G., and Bochner, B.H. (2006). Impact of renal impairment on eligibility for adjuvant cisplatin-based chemotherapy in patients with urothelial carcinoma of the bladder. Cancer *107*, 506–513.
5. Galsky, M.D., Hahn, N.M., Rosenberg, J., Sonpavde, G., Hutson, T., Oh, W.K., Dreicer, R., Vogelzang, N., Sternberg, C.N., Bajorin, D.F., and Bellmunt, J. (2011). Treatment of patients with metastatic urothelial cancer "unfit" for Cisplatin-based chemotherapy. J. Clin. Oncol. *29*, 2432–2438.
6. Bajorin, D.F., Dodd, P.M., Mazumdar, M., Fazzari, M., McCaffrey, J.A., Scher, H.I., Herr, H., Higgins, G., and Boyle, M.G. (1999). Long-term survival in metastatic transitional-cell carcinoma and prognostic factors predicting outcome of therapy. J. Clin. Oncol. *17*, 3173–3181.
7. Rhea, L.P., Mendez-Marti, S., Kim, D., and Aragon-Ching, J.B. (2021). Role of immunotherapy in bladder cancer. Cancer Treat. Res. Commun. *26*, 100296.
8. Addeo, A., Banna, G.L., Metro, G., and Di Maio, M. (2019). Chemotherapy in combination with immune checkpoint inhibitors for the first-line treatment of patients with advanced non-small cell lung cancer: a systematic review and literature-based meta-analysis. Front. Oncol. *9*, 264.
9. Cubas, R., Moskalenko, M., Cheung, J., Yang, M., McNamara, E., Xiong, H., Hoves, S., Ries, C.H., Kim, J., and Gould, S. (2018). Chemotherapy combines effectively with anti-PD-L1 treatment and can augment antitumor responses. J. Immunol. *201*, 2273–2286.
10. Galsky, M.D., Arija, J.Á.A., Bamias, A., Davis, I.D., De Santis, M., Kikuchi, E., Garcia-Del-Muro, X., De Giorgi, U., Mencinger, M., Izumi, K., et al. (2020). Atezolizumab with or without chemotherapy in metastatic urothelial cancer (IMvigor130): a multicentre, randomised, placebo-controlled phase 3 trial. Lancet *395*, 1547–1557.
11. Powles, T., Csőszi, T., Özgüroğlu, M., Matsubara, N., Géczi, L., Cheng, S.Y.S., Fradet, Y., Oudard, S., Vulsteke, C., Morales Barrera, R., et al. (2021). Pembrolizumab alone or combined with chemotherapy versus chemotherapy as first-line therapy for advanced urothelial carcinoma (KEYNOTE-361): a randomised, open-label, phase 3 trial. Lancet Oncol. *22*, 931–945.
12. Powles, T., Park, S.H., Voog, E., Caserta, C., Valderrama, B.P., Gurney, H., Kalofonos, H., Radulović, S., Demey, W., Ullén, A., et al. (2020). Avelumab maintenance therapy for advanced or metastatic urothelial carcinoma. N. Engl. J. Med. *383*, 1218–1230.
13. de Biasi, A.R., Villena-Vargas, J., and Adusumilli, P.S. (2014). Cisplatin-induced antitumor immunomodulation: a review of preclinical and clinical evidence. Clin. Cancer Res. *20*, 5384–5391.
14. Hato, S.V., Khong, A., de Vries, I.J.M., and Lesterhuis, W.J. (2014). Molecular pathways: the immunogenic effects of platinum-based chemotherapeutics. Clin. Cancer Res. *20*, 2831–2837.
15. Denkert, C., Loibl, S., Noske, A., Roller, M., Müller, B.M., Komor, M., Budczies, J., Darb-Esfahani, S., Kronenwett, R., Hanusch, C., et al. (2010). Tumor-associated lymphocytes as an independent predictor of response to neoadjuvant chemotherapy in breast cancer. J. Clin. Oncol. *28*, 105–113.
16. Galsky, M.D., Hahn, N.M., Rosenberg, J., Sonpavde, G., Hutson, T., Oh, W.K., Dreicer, R., Vogelzang, N., Sternberg, C., Bajorin, D.F., and Bellmunt, J. (2011). A consensus definition of patients with metastatic urothelial carcinoma who are unfit for cisplatin-based chemotherapy. Lancet Oncol. *12*, 211–214.
17. Herbst, R.S., Soria, J.C., Kowanetz, M., Fine, G.D., Hamid, O., Gordon, M.S., Sosman, J.A., McDermott, D.F., Powderly, J.D., Gettinger, S.N., et al. (2014). Predictive correlates of response to the anti-PD-L1 antibody MPDL3280A in cancer patients. Nature *515*, 563–567.
18. Tumeh, P.C., Harview, C.L., Yearley, J.H., Shintaku, I.P., Taylor, E.J.M., Robert, L., Chmielowski, B., Spasic, M., Henry, G., Ciobanu, V., et al. (2014). PD-1 blockade induces responses by inhibiting adaptive immune resistance. Nature *515*, 568–571.
19. Hiam-Galvez, K.J., Allen, B.M., and Spitzer, M.H. (2021). Systemic immunity in cancer. Nat. Rev. Cancer *21*, 345–359.

20. van den Elsen, P.J., Holling, T.M., Kuipers, H.F., and van der Stoep, N. (2004). Transcriptional regulation of antigen presentation. Curr. Opin. Immunol. *16*, 67–75.

21. Oh, S.A., Wu, D.C., Cheung, J., Navarro, A., Xiong, H., Cubas, R., Totpal, K., Chiu, H., Wu, Y., Comps-Agrar, L., et al. (2020). PD-L1 expression by dendritic cells is a key regulator of T-cell immunity in cancer. Nat. Can. (Ott.) *1*, 681–691.

22. Lokich, J., and Anderson, N. (1998). Carboplatin versus cisplatin in solid tumors: an analysis of the literature. Ann. Oncol. *9*, 13–21.

23. Vermorken, J.B., ten Bokkel Huinink, W.W., Eisenhauer, E.A., Favalli, G., Belpomme, D., Conte, P.F., and Kaye, S.B. (1993). Advanced ovarian cancer. Carboplatin versus cisplatin. Ann. Oncol. *4*, 41–48.

24. van der Vijgh, W.J. (1991). Clinical pharmacokinetics of carboplatin. Clin. Pharmacokinet. *21*, 242–261.

25. Emens, L.A., and Middleton, G. (2015). The interplay of immunotherapy and chemotherapy: harnessing potential synergies. Cancer Immunol. Res. *3*, 436–443.

26. Glorieux, C., Cui, L., Zeng, P., Xia, X., and Huang, P. (2021). Diverse effects of chemotherapeutic agents on immune cell function and implications in immunochemotherapy. Cancer Commun. *41*, 432–435.

27. Kern, W., Braess, J., Friedrichsen, S., Kaufmann, C.C., Schleyer, E., and Hiddemann, W. (2001). Carboplatin pharmacokinetics in patients receiving carboplatin and paclitaxel/docetaxel for advanced lung cancers: impact of age and renal function on area under the curve. J. Cancer Res. Clin. Oncol. *127*, 64–68.

28. Rajkumar, P., Mathew, B.S., Das, S., Isaiah, R., John, S., Prabha, R., and Fleming, D.H. (2016). Cisplatin concentrations in long and short duration infusion: implications for the optimal time of radiation delivery. J. Clin. Diagn. Res. *10*, Xc01-xc04.

29. Gomes, L.R., Rocha, C.R.R., Martins, D.J., Fiore, A.P.Z.P., Kinker, G.S., Bruni-Cardoso, A., and Menck, C.F.M. (2019). ATR mediates cisplatin resistance in 3D-cultured breast cancer cells via translesion DNA synthesis modulation. Cell Death Dis. *10*, 459.

30. Pabla, N., Huang, S., Mi, Q.S., Daniel, R., and Dong, Z. (2008). ATR-Chk2 signaling in p53 activation and DNA damage response during cisplatin-induced apoptosis. J. Biol. Chem. *283*, 6572–6583.

31. Yazlovitskaya, E.M., and Persons, D.L. (2003). Inhibition of cisplatin-induced ATR activity and enhanced sensitivity to cisplatin. Anticancer Res. *23*, 2275–2279.

32. Reaper, P.M., Griffiths, M.R., Long, J.M., Charrier, J.D., Maccormick, S., Charlton, P.A., Golec, J.M.C., and Pollard, J.R. (2011). Selective killing of ATM- or p53-deficient cancer cells through inhibition of ATR. Nat. Chem. Biol. *7*, 428–430.

33. Ma, Y., Adjemian, S., Mattarollo, S.R., Yamazaki, T., Aymeric, L., Yang, H., Portela Catani, J.P., Hannani, D., Duret, H., Steegh, K., et al. (2013). Anticancer chemotherapy-induced intratumoral recruitment and differentiation of antigen-presenting cells. Immunity *38*, 729–741.

34. Prokhnevska, N., Cardenas, M.A., Valanparambil, R.M., Sobierajska, E., Barwick, B.G., Jansen, C., Reyes Moon, A., Gregorova, P., delBalzo, L., Greenwald, R., et al. (2023). CD8(+) T cell activation in cancer comprises an initial activation phase in lymph nodes followed by effector differentiation within the tumor. Immunity *56*, 107–124.e5.

35. Apetoh, L., Ghiringhelli, F., Tesniere, A., Obeid, M., Ortiz, C., Criollo, A., Mignot, G., Maiuri, M.C., Ullrich, E., Saulnier, P., et al. (2007). Toll-like receptor 4-dependent contribution of the immune system to anticancer chemotherapy and radiotherapy. Nat. Med. *13*, 1050–1059.

36. Han, J., Zhong, J., Wei, W., Wang, Y., Huang, Y., Yang, P., Purohit, S., Dong, Z., Wang, M.H., She, J.X., et al. (2008). Extracellular high-mobility group box 1 acts as an innate immune mediator to enhance autoimmune progression and diabetes onset in NOD mice. Diabetes *57*, 2118–2127.

37. Chen, D.S., and Mellman, I. (2013). Oncology meets immunology: the cancer-immunity cycle. Immunity *39*, 1–10.

38. Sadagopan, A., Michelakos, T., Boyiadzis, G., Ferrone, C., and Ferrone, S. (2022). Human leukocyte antigen class I antigen-processing machinery upregulation by anticancer therapies in the era of checkpoint inhibitors: a review. JAMA Oncol. *8*, 462–473.

39. Blommaert, F.A., Michael, C., Terheggen, P.M., Muggia, F.M., Kortes, V., Schornagel, J.H., Hart, A.A., and den Engelse, L. (1993). Drug-induced DNA modification in buccal cells of cancer patients receiving carboplatin and cisplatin combination chemotherapy, as determined by an immunocytochemical method: interindividual variation and correlation with disease response. Cancer Res. *53*, 5669–5675.

40. Atkins, M.B., Hsu, J., Lee, S., Cohen, G.I., Flaherty, L.E., Sosman, J.A., Sondak, V.K., and Kirkwood, J.M.; Eastern Cooperative Oncology Group (2008). Phase III trial comparing concurrent biochemotherapy with cisplatin, vinblastine, dacarbazine, interleukin-2, and interferon alfa-2b with cisplatin, vinblastine, and dacarbazine alone in patients with metastatic malignant melanoma (E3695): a trial coordinated by the Eastern Cooperative Oncology Group. J. Clin. Oncol. *26*, 5748–5754.

41. Buzaid, A.C. (2000). Strategies for combining chemotherapy and biotherapy in melanoma. Cancer Control *7*, 185–197.

42. Legha, S.S., Ring, S., Eton, O., Bedikian, A., Buzaid, A.C., Plager, C., and Papadopoulos, N. (1998). Development of a biochemotherapy regimen with concurrent administration of cisplatin, vinblastine, dacarbazine, interferon alfa, and interleukin-2 for patients with metastatic melanoma. J. Clin. Oncol. *16*, 1752–1759.

43. Rosenberg, S.A., Yang, J.C., Schwartzentruber, D.J., Hwu, P., Marincola, F.M., Topalian, S.L., Seipp, C.A., Einhorn, J.H., White, D.E., and Steinberg, S.M. (1999). Prospective randomized trial of the treatment of patients with metastatic melanoma using chemotherapy with cisplatin, dacarbazine, and tamoxifen alone or in combination with interleukin-2 and interferon alfa-2b. J. Clin. Oncol. *17*, 968–975.

44. Grabosch, S., Bulatovic, M., Zeng, F., Ma, T., Zhang, L., Ross, M., Brozick, J., Fang, Y., Tseng, G., Kim, E., et al. (2019). Cisplatin-induced immune modulation in ovarian cancer mouse models with distinct inflammation profiles. Oncogene *38*, 2380–2393.

45. Rébé, C., Demontoux, L., Pilot, T., and Ghiringhelli, F. (2019). Platinum derivatives effects on anticancer immune response. Biomolecules *10*, 13.

46. Fournel, L., Wu, Z., Stadler, N., Damotte, D., Lococo, F., Boulle, G., Ségal-Bendirdjian, E., Bobbio, A., Icard, P., Trédaniel, J., et al. (2019). Cisplatin increases PD-L1 expression and optimizes immune check-point blockade in non-small cell lung cancer. Cancer Lett. *464*, 5–14.

47. Bristol Myers Squibb. Opdivo (nivolumab) in Combination with Cisplatin-Based Chemotherapy Shows Overall Survival and Progression-Free Survival Benefit for Cisplatin-Eligible Patients with Unresectable or Metastatic Urothelial Carcinoma in the Phase 3 CheckMate -901 Trial. https://news.bms.com/news/details/2023/Opdivo-nivolumab-in-Combination-with-Cisplatin-Based-Chemotherapy-Shows-Overall-Survival-and-Progression-Free-Survival-Benefit-for-Cisplatin-Eligible-Patients-with-Unresectable-or-Metastatic-Urothelial-Carcinoma-in-the-Phase-3-CheckMate–901-Trial/default.aspx.

48. Principe, D.R., Narbutis, M., Kumar, S., Park, A., Viswakarma, N., Dorman, M.J., Kamath, S.D., Grippo, P.J., Fishel, M.L., Hwang, R.F., et al. (2020). Long-term gemcitabine treatment reshapes the pancreatic tumor microenvironment and sensitizes murine carcinoma to combination immunotherapy. Cancer Res. *80*, 3101–3115.

49. Wu, T.D., Reeder, J., Lawrence, M., Becker, G., and Brauer, M.J. (2016). GMAP and GSNAP for genomic sequence alignment: Enhancements to speed, accuracy, and functionality. Methods Mol. Biol. *1418*, 283–334.

50. Wu, T.D., and Nacu, S. (2010). Fast and SNP-tolerant detection of complex variants and splicing in short reads. Bioinformatics *26*, 873–881.

51. Stuart, T., Butler, A., Hoffman, P., Hafemeister, C., Papalexi, E., Mauck, W.M., 3rd, Hao, Y., Stoeckius, M., Smibert, P., and Satija, R. (2019). Comprehensive integration of single-cell data. Cell *177*, 1888–1902.e21.

52. Korsunsky, I., Millard, N., Fan, J., Slowikowski, K., Zhang, F., Wei, K., Baglaenko, Y., Brenner, M., Loh, P.R., and Raychaudhuri, S. (2019). Fast,

sensitive and accurate integration of single-cell data with Harmony. Nat. Methods *16*, 1289–1296.

53. Lawrence, M., Huber, W., Pagès, H., Aboyoun, P., Carlson, M., Gentleman, R., Morgan, M.T., and Carey, V.J. (2013). Software for computing and annotating genomic ranges. PLoS Comput. Biol. *9*, e1003118.

54. Stoeckius, M., Hafemeister, C., Stephenson, W., Houck-Loomis, B., Chattopadhyay, P.K., Swerdlow, H., Satija, R., and Smibert, P. (2017). Simultaneous epitope and transcriptome measurement in single cells. Nat. Methods *14*, 865–868.

55. Ritchie, M.E., Phipson, B., Wu, D., Hu, Y., Law, C.W., Shi, W., and Smyth, G.K. (2015). limma powers differential expression analyses for RNA-sequencing and microarray studies. Nucleic Acids Res. *43*, e47.

56. Korotkevich, G., Sukhov, V., Budin, N., Shpak, B., Artyomov, M.N., and Sergushichev, A. (2021). Fast gene set enrichment analysis. Preprint at bioRxiv. https://doi.org/10.1101/060012.

57. Hänzelmann, S., Castelo, R., and Guinney, J. (2013). GSVA: gene set variation analysis for microarray and RNA-seq data. BMC Bioinf. *14*, 7.

STAR★METHODS

KEY RESOURCES TABLE

REAGENT or RESOURCE	SOURCE	IDENTIFIER
Antibodies		
Mouse BD Fc block	BD Biosciences	Cat # 553142; RRID: AB_394656
PE anti-mouse PDL1	Biolegend	Cat # 155404; RRID: AB_2728222
AF700 anti-mouse CD8	BD Biosciences	Cat # 557959; RRID: AB_396959
APC anti-mouse CD45	BD Biosciences	Cat # 559864; RRID: AB_398672
Human TruStain FcX	Biolegend	Cat # 422302; RRID: AB_2818986
PE anti-human PD-L1	Biolegend	Cat # 329706; RRID: AB_940368
Pacific Blue anti-mouse CD40	BioLegend	Cat # 124626; RRID: AB_2561476
BUV395 Rat Anti-Mouse H-2 Class I	BD Biosciences	Cat # 749705; RRID: AB_2873959
PE rat anti-mouse CD86	BD Biosciences	Cat # 553692; RRID:AB_394994
APC rat anti-mouse CD209a	BD Biosciences	Cat # 564928; RRID:AB_2739011
BV421 anti-human CD4	Biolegend	Cat # 317434; RRID: AB_2562134
BUV805 mouse anti-human CD8	BD Biosciences	Cat # 612889; RRID: AB_2833078
FITC Ki-67 monoclonal antibody	eBiosciences	Cat # 11-5698-82; RRID: AB_11151330
BV786 mouse anti-human CD69	BD Biosciences	Cat # 563834; RRID: AB_2738441
PE-Cy7 mouse anti-human IFN-γ	BD Biosciences	Cat # 557643; RRID: AB_396760
Annexin V- PE	BD Biosciences	Cat # 556421; RRID: AB_2869071
7-AAD	BD Biosciences	Cat # 559925; RRID: AB_2869266
p-ATM S1981	Cell Signaling Technology	Cat #5883
p-ATR T1989	Cell Signaling Technology	Cat #30632; RRID: AB_2798992
p-Chk1 S317	Cell Signaling Technology	Cat #12302; RRID: AB_2893473
p-Chk2 T68	Cell Signaling Technology	Cat #2197; RRID: AB_331479
GAPDH	Cell Signaling Technology	Cat #2118; RRID: AB_561053
Peroxidase AffiniPure goat anti-rabbit IgG (H + L)	Jackson Immunoresearch	Cat #111-035-144; RRID: AB_2307391
Biological samples		
IMvigor130 mUC samples	Genentech	N/A
IMvigor130 PBMC collection for CITE-seq (n = 113)	This study	N/A
Chemicals, peptides, and recombinant proteins		
Cisplatin	Sigma Aldrich	CAS#: 15663-27-1
Carboplatin	Sigma Aldrich	CAS#: 41575-94-4
KU-55933 (ATM Kinase Inhibitor)	Selleckchem	Cat#: S1092
VE-821(ATR inhibitor IV)	Selleckchem	Cat#: S8007
Halt protease and phosphatase inhibitor cocktail, EDTA-free	Thermo Fisher	Cat#: 78441
Critical commercial assays		
10x Chromium Next GEM Chip G Kit	10X Genomics	
Pan T cell Isolation Kit, human	Miltenyi Biotec	Cat# 130-096-535
Dynabeads Human T-Activator CD3/CD28 for T cell expansion and activation	Thermo Fisher	Cat# 11161D
CD14 MicroBeads, human	Miltenyi Biotec	Cat# 130-050-201
CellXVivo human monocyte-derived DC differentiation kit	R&D	Cat# CDK004
Cytiva Ficoll-Paque PLUS Media	Fisher Scientific	Cat# 45-001-749
Human IFNg ELISA kit	BD Biosciences	Cat.# 555142

(*Continued on next page*)

Continued

REAGENT or RESOURCE	SOURCE	IDENTIFIER
Mouse CXCL10 ELISA kit	Thermo Fisher Scientific	Cat# BMS6018
Mouse HMGB1 ELISA kit	Tecan	Cat# 30164033
CellTrace Blue Cell Proliferation kit	Thermo Fisher Scientific	Cat# C34568
RNeasy kits	Qiagen	
FITC Annexin V Apoptosis Detection Kit with 7-AAD	BD Biosciences	Cat#: 556547
RIPA buffer	Sigma	
LIVE/DEAD Fixable Aqua Dead Cell Stain Kit	Invitrogen	L34957
**Deposited data**		
Raw and analyzed bulk RNAseq (cell lines)	This paper	GEO: GSE235066
PBMC scRNA-seq raw counts and metadata	This paper	EGAS50000000104
IMvigor130 bulk RNAseq processed data (patient tumors)	This paper	EGAS50000000104
**Experimental models: Cell lines**		
Human cell line: 5637	Genentech	N/A
Human cell line: RT112	Genentech	N/A
Mouse cell line: MC38	Genentech	N/A
Mouse cell line: MC38-OVA	Genentech	N/A
**Experimental models: Organisms/strains**		
C57BL/6		
C57BL/6J.OT-I.Thy1.1		
**Software and algorithms**		
R statistical software, version 4.0.2	The R Foundation	https://www.r-project.org/
GSNAP	Wu et al., 2016[49]; Wu et al., 2010[50]	
survminer package, version 0.4.8	CRAN Repository	https://cran.r-project.org/web/packages/survminer/index.html
survival package, version 3.2–7	CRAN Repository	https://cran.r-project.org/web/packages/survival/index.html
limma package, 3.44.3	Bioconductor	https://bioconductor.org/packages/release/bioc/html/limma.html
fgsea package, 1.14.0	Bioconductor	http://bioconductor.org/packages/fgsea
CellRanger, 3.1.0	10X Genomics	http://10xgenomics.com/
Seurat, v3.2.2	Stuart et al., 2019[51]	https://satijalab.org/seurat
Harmony, v1.0	Korsunsky et al., 2019[52]	https://github.com/immunogenomics/harmony
FlowJo	Becton Dickinson	https://flowjo.com/
Graphpad Prism 7.0 software	GraphPad Software, Inc.	http://www.graphpad.com/scientific software/prism/
GSVA	Bioconductor	https://bioconductor.org/packages/release/bioc/html/GSVA.html

RESOURCE AVAILABILITY

Lead contact

Further information and requests for resources and reagents should be directed to and will be fulfilled by the lead contact, Sanjeev Mariathasan (sanj@gene.com).

Materials availability

This study did not generate new unique reagents.

Data and code availability

- As this study is ongoing, access to patient-level data from this trial will not be available until at least 18 months after the last patient visit and completion of a clinical study report. After that time, requests for data should be submitted to lead contact,

Sanjeev Mariathasan (sanj@gene.com). For up-to-date details on Roche's Global Policy on the Sharing of Clinical Information and how to request access to related clinical study documents, see: https://go.roche.com/data_sharing. Anonymized records for individual patients across more than one data source external to Roche cannot be linked due to a potential increase in risk of patient reidentification.

- The accession number for the scRNA-seq reported in this paper is European Genome-Phenome Archive: EGAS50000000104.
- The IMvigor130 bulk RNAseq dataset is available with accession number European Genome-Phenome Archive: EGAS50000000104.
- The accession number for the bulk RNAseq of the cisplatin- and carboplatin-treated tumor cell lines *in vitro* is GEO: GSE235066.
- This paper does not report original code.
- Any additional information required to reanalyze the data reported in this paper is available from the lead contact upon request.

EXPERIMENTAL MODEL AND STUDY PARTICIPANT DETAILS

Clinical samples

Patients enrolled in the phase 3 IMvigor130 trial (NCT02807636) were randomized to receive atezolizumab with or without platinum-based chemotherapy, or placebo plus platinum-based chemotherapy, as first-line treatment of mUC. All patients gave informed consent and studies were approved by their respective ethical review committees. For specific details of ethical review and study designs, see original publications.[10]

Tumor tissues were evaluated for PD-L1 expression using the SP142 immunohistochemistry assay (Ventana Medical Systems, Inc.). PD-L1 IC2/3 and IC0/1 indicated PD-L1–expressing tumor-infiltrating ICs covering $\geq$5% and <5%, of the tumor area, respectively. Bulk RNA sequencing (RNA-seq) was performed on pretreatment tumor specimens for gene set enrichment analysis according to PD-L1 status (IC2/3 vs. IC0/1), treatment arm and the specific platinum drug received. A total of 113 patients had available baseline (C1D1) and on-treatment (C3D1) PBMC samples that were processed for single-cell RNA sequencing (scRNA-seq), coupled with single-cell T cell receptor sequencing (scTCR-seq), single-cell B cell receptor sequencing (scBCR-seq), and CITE-seq.

Blood samples from healthy donors were supplied through the Samples for Science program, an institutional review board–approved research program operated through the Genentech Campus Health Center.

Cell lines

Human bladder carcinoma cell lines (5637 and RT112) and murine colorectal carcinoma cell lines (MC38 and MC38-OVA) were obtained from Genentech's common cell bank. All cell lines were cultured in Roswell Park Memorial Institute medium (RPMI) 1640 containing 10% heat-inactivated fetal bovine serum (FBS), 100 U/mL penicillin, 100 mg/mL streptomycin sulfate, and 1% L-glutamine. All cell lines were validated as mycoplasma free by polymerase chain reaction tests.

Animal strains

C57BL/6 mice and C57BL/6J.OT-I.Thy1.1 TCR transgenic mice were bred and housed at Genentech in standard rodent microisolator cages. Female mice were used for all studies and were 6–8 weeks old at the start of experiments. Experimental animals were housed at Genentech in special rodent isolator cages. All animal activities in this research study were conducted under protocols approved by the Genentech Institutional Animal Care and Use Committee.

METHOD DETAILS

Analysis of IMvigor130 bulk RNA-seq

Whole-transcriptome profiles were generated using TruSeq RNA Access technology (Illumina). RNA-seq reads were first aligned to ribosomal RNA sequences to remove ribosomal reads. Remaining reads were aligned to the human reference genome (NCBI Build 38) using GSNAP v.2013-10-10,[50,49] allowing a maximum of two mismatches per 75-base sequence (parameters, '-M 2 -n 10 -B 2 -i 1 -N 1 -w 200,000 -E 1-pairmax-rna = 200,000 –clip-overlap'). To quantify gene expression levels, the number of reads mapped to the exons of each RefSeq gene was calculated using the functionality provided by the R/Bioconductor package GenomicAlignments.[53]

10X genomics scRNA-seq library construction and sequencing

Previously frozen PBMCs were thawed, washed twice in RPMI 2% fetal calf serum, treated with the ammonium-chloride-potassium (ACK) lysis buffer (Lonza) to remove red blood cells (RBCs), and briefly incubated with 4′,6-diamidino-2-phenylindole (DAPI). For each sample, 300,000 live cells were sorted on a DAPI-negative gate, stained for 30 min at room temperature with a custom panel of 59 Total-Seq-C antibodies (BioLegend[54]) and corresponding hashtag Total-Seq-C (BioLegend), and washed three times using the HT1000 laminar wash system (Curiox). Cells were then counted using the Cellaca MX High-Throughput Automated Cell Counter (Nexcelom), pooled from five samples, and loaded on the 10x Chromium Next GEM Chip G Kit using a superloading strategy. B cell receptor (BCR) and T cell receptor (TCR) CDR3 sequences were enriched using the human V(D)J B/T cell enrichment. Libraries

were prepared according to the manufacturer's protocol (10x Genomics) and sequenced on a NovaSeq 6000 System using the S4 2x 150 kit (Illumina).

Preprocessing of scRNA-seq, CITE-seq, and VDJ data

The scRNA-seq raw reads were aligned to the human transcriptome (GRCh38), and unique molecular identifier (UMI) counts were quantified to generate a gene-barcode matrix using the Cell Ranger pipeline (10X Genomics, Cell Ranger v3.1.0). CITE-seq antibody expression matrices were generated using the Cell Ranger pipeline (10X Genomics, Cell Ranger v3.1.0). BCR and TCR reads were aligned to the GRCh38 reference genome, and consensus BCR/TCR annotation was performed using the Cell Ranger vdj pipeline (10X Genomics, Cell Ranger v3.1.0). To assign cells to their respective samples of origin, cells were demultiplexed with a modified HTOdemux function from the Seurat package, whereby the negative cluster was defined by minimal nonzero expression.

Cluster analysis of peripheral blood immune cells

The preprocessed gene expression matrix generated by the Cell Ranger pipeline was imported into Seurat (version 3.2.2) for downstream analysis.[51] As a quality control step, genes that were expressed in <10 cells were removed, and cells were kept based on conservative predefined hard cutoffs—including the number of detected genes (range, 200–5000), number of detected UMIs (range, 1000–20,000), house-keeping gene expression ($\geq$10 genes), and percentage of mitochondrial gene expression ($\leq$10%)—and a dataset-specific cutoff computed using interquartile ranges. In addition, RBC and platelet contaminants were removed via automated filtering algorithms. The filtered gene expression matrix (16,315 genes × 865,922 cells) was normalized using the NormalizeData function (normalization.method = "LogNormalize" and scale.factor = 10000). The surface protein expression matrix was normalized using the centered-log ratio method. Variable genes were identified using the FindVariableFeatures function with default parameters. Prior to dimension reduction, the data were scaled, and the effects of variation in UMI counts and percentage mitochondrial contents were regressed out (the ScaleData function). Principal component analysis was then performed on the scaled data cut to the variable genes. Batch effects were mitigated using the Harmony (version 1.0) package.[52] Shared nearest neighbors were computed, and cells were then clustered using graph community clustering methods. A uniform manifold approximation and projection (UMAP) was generated using the RunUMAP function. Cells were annotated using a cell type classifier, taking into account RNA, surface proteins, and TCR sequences, and was further validated and refined using Immunai's curated in-house signatures. Multiomic data were further used to remove low-quality cells and previously undetected doublets (e.g., cells that express both CD8 and CD4 protein tags, and cells that express a high B-cell signature and have a detected TCR).

Pseudo-bulk differential gene expression analysis of PBMCs

Differential gene expression (DEG) tests were performed by pseudo-bulk analysis, in which gene counts were aggregated (summed) for each sample and cell type. Samples per cell type that had <10 cells were removed. Differential expression analysis was performed with the limma-voom R package (version 3.44.3) for each cell type independently.[55] In DEG analysis comparing on-treatment (C3D1) versus pretreatment (C1D1) samples, patient ID was added as a covariate to the design formula to consider the paired design. Patients without matching pretreatment and on-treatment samples were removed. The moderated *t*-statistics from limma DEG tests were used as a pre-ranked gene list input for pathway enrichment analysis, which was performed using the *fgsea* R package (version 1.14.0).[56] In this analysis, we used the Hallmark gene set, as well as gene signatures related to T- and NK-cell activation and functions, collected from MSigDB (version 7.2).

In vitro drug treatment on tumor cell lines and flow cytometry characterization

Human bladder cancer cells (5637 and RT112) and murine colon cancer cells (MC38) were treated with various concentrations of cisplatin or carboplatin for 24 h. For the ATM and ATR inhibitor assays, the tumor cells were also treated with an ATM inhibitor (KU-55933, 1 μM) or ATR inhibitor (VE-821, 1 μM) together with cisplatin or carboplatin. Cells were collected into single-cell suspensions, resuspended in the staining buffer, and then labeled with indicated antibodies for 20 min at 4°C. Fc receptors were blocked prior to surface antibody staining using Mouse BD Fc Block (BD Biosciences, clone 2.4G2) or Human TruStain FcX. The antibodies used were PD-L1 (clone 29E.2.A3) for human tumor cells and PD-L1 (clone MIH7) for mouse tumor cells. The FITC Annexin V Apoptosis Detection Kit with 7-aminoactinomycin D (7-AAD) (BD Biosciences) was used to stain treated cells to evaluate potential cell death induced by cisplatin or carboplatin.

Tumor cell line bulk RNA-seq processing

To profile the impact of cisplatin and carboplatin on the whole transcriptomes of tumor cells, human bladder cancer cells (5637 and RT112) and murine colon cancer cells (MC38) were treated with cisplatin (5 μM), carboplatin (35 μM), or control for 24 h. In addition, we collected 5637 cells that had been treated for 24 h with cisplatin (5 μM) or carboplatin (35 μM) in the presence or absence of an ATR inhibitor (1 μM) to evaluate the role of DNA damage sensor ATR in cisplatin- or carboplatin-mediated transcriptome changes.

Total RNA was enriched from treated cells using RNeasy Mini kits according to the manufacturer's instructions (Qiagen). Total RNA was quantified with the Qubit RNA HS Assay Kit (Thermo Fisher Scientific), and quality was assessed using RNA ScreenTape on 4200 TapeStation (Agilent Technologies). For sequencing-library generation, the Truseq Stranded mRNA kit (Illumina) was used with an input of 100 ng of total RNA. Libraries were quantified with the Qubit dsDNA HS Assay Kit (Thermo Fisher Scientific), and the average

library size was determined using D1000 ScreenTape on 4200 TapeStation (Agilent Technologies). Libraries were pooled and sequenced on NovaSeq 6000 (Illumina) to generate 30 million single-end 50-base pair reads for each sample.

Analysis of tumor cell line bulk RNA-seq

Raw FASTQ file alignment and gene expression quantification were performed as described above. The raw counts were imported in the edgeR-voom-limma pipeline, and the normalized, log-transformed values were used to calculate gene set signature scores for the Hallmark pathways from the MSigDB database at the individual sample level using the GSVA package (v1.38.2) with the default method.[57] Gene sets that were significantly altered between contrast groups were called based on limma analysis. Pathways with a false-discovery rate of <0.05 were considered significant.

Immunoblotting

Human bladder cancer cells (5637) were treated with various concentrations of cisplatin or carboplatin for 6 h. Cells were collected and lysed in RIPA buffer (Sigma) supplemented with Halt Protease and Phosphatase Inhibitor Cocktail (Thermo Fisher Scientific). Cell lysates were run on a 4%–12% sodium dodecyl sulfate-polyacrylamide gel, transferred, and immunoblotted with Cell Signaling Technology antibodies for *p*-ATM S1981 (#5883), *p*-ATR T1989 (#30632), *p*-Chk1 S317 (#12302), *p*-Chk2 T68 (#2197), and GAPDH (#2118). Goat anti-rabbit horseradish peroxidase–conjugated secondary antibodies (#111-035-144) were from Jackson ImmunoResearch Laboratories.

In vitro human T cell activation and drug treatment

Human PBMCs were isolated from whole blood of healthy donors by Ficoll density gradient centrifugation. Human primary T cells were isolated from healthy PBMCs by using a Pan T cell Isolation Kit (Miltenyi Biotec, Cat# 130-096-535) per the manufacturer's instructions. The pan-T cells were cultured in RPMI 1640 medium supplemented with CD3/CD28 Dynabeads (Thermo Fisher Scientific, Cat# 11161D) for 48 h. Cisplatin and carboplatin were added at different concentrations during activation. Cells were harvested for intracellular staining followed by fluorescence-activated cell sorting (FACS) analyses.

In vitro human DC differentiation and mixed lymphocyte reaction (MLR) assay

Human $CD14^+$ monocytes were isolated from healthy PBMCs by using CD14 MicroBeads (Miltenyi Biotec, Cat# 130-050-201) per the manufacturer's instructions. The monocytes were differentiated into DCs by using a DC differentiation kit (R&D, Cat# CDK004) per the manufacturer's instructions. Briefly, isolated monocytes were cultured in the cell medium with granulocyte-macrophage colony-stimulating factor (GM-CSF) and IL-4 for 5 days. On day 5 of the differentiation, tumor necrosis factor α was supplemented and cisplatin and carboplatin were added at different concentrations. DCs were harvested on day 7 for FACS staining of DC activation markers. To set up the MLR assay, donor-mismatched DCs and pan-T cells were mixed in a 1:5 ratio and cultured for 5 days in the presence of different concentrations of cisplatin and carboplatin. Cell supernatants were collected for enzyme-linked immunosorbent assay (ELISA) analyses.

Co-culture of murine DCs and MC38 cells

Mouse DCs were derived from C57BL/6 bone marrow progenitor cells that were incubated in 100 mL of DC medium supplemented with 20 ng/mL each of mouse GM-CSF (R&D Systems, Cat# 415-ML-010/CF) and mouse IL-4 (R&D Systems, Cat# 404-ML-010/CF) and incubated for 3 days at 37°C in a 25-mm cell culture Petri dish. On the third day, 50 mL of additional medium supplemented with human GM-CSF and human IL-4 was added to the cells, and cells were allowed to incubate for 3 more days before use. Murine colon tumor cells (MC38) were plated, 10,000 cells/well, in a 96-well round-bottom plate and incubated at 37°C overnight. The following day, the medium was exchanged, and cells were pretreated with increasing concentrations of cisplatin or carboplatin for 24 h. DCs were added to the tumor cells at 15,000 cells/well, and the co-cultured MC38 cells and DCs were incubated for 24 h. Cells were then washed twice using FACS stain buffer (BD Biosciences) and resuspended in a live/dead 7-AAD dye (BD Biosciences, Catalog# 559925) before flow cytometry profiling of surface marker expression on DCs, including CD45 (BioLegend, Cat# 103151), CD11c (BioLegend, Cat# 117320), MHC-I (BD Biosciences, Cat# 749705), PD-L1 (BioLegend, Cat# 124308), CD40 (BioLegend, Cat# 124626), and CD86 (BioLegend, Cat# 105040). The flow cytometry data were collected with a BD LSRFortessa (BD Biosciences) and analyzed using FlowJo software (version 10.6.2, FlowJo LLC).

OT-I T cell proliferation assay

MC38 colon adenocarcinoma cells that express EGFP along with an equal copy number of full-length wild-type chicken ovalbumin (OVA) protein (MC38-OVA) were obtained from the common cell repository at Genentech Inc. The ovalbumin contains the wild-type SIINFEKL peptide. MC38-OVA cells were plated in tumor culture medium in 96-well U-bottom cell culture plates at a density of 10,000 cells/well. Cells were allowed to adhere overnight at 37°C. The following day, cells were spun at 1550 RPM for 5 min at room temperature. The supernatant was removed from the wells, and the 24-h treatment was started. Cisplatin or carboplatin was diluted in T cell medium (RPMI 1640 + 10% FBS +1% penicillin/streptomycin, 1% HEPES, 1% sodium pyruvate, and 1% glutamax) using a 3-fold dose titration. Medium containing chemotherapy drugs was added to the MC38-OVA tumor cells, and plates were incubated overnight at 37°C. The following day, OT-I $CD8^+$ T cells labeled with the CellTrace Blue Cell proliferation kit (Thermo Fisher Scientific,

Cat# C34568) were added at a density of 50,000 cells/well in the presence or absence of DCs. The co-cultures were incubated for an additional 3 days at 37°C before cell proliferation measurement by flow cytometry. Briefly, cells were first incubated with mouse FcR blocking reagent (Miltenyi Biotec, Cat# 130-092-575), followed by a cocktail of antibodies (CD8, BioLegend, Cat# 100730; CD45 BioLegend, Cat# 103140) and staining for 30 min at room temperature. The cells were then washed twice using FACS stain buffer (BD Biosciences, Catalog# 554656) and resuspended in a live/dead7-AAD dye (BD Biosciences, Catalog# 559925). The flow cytometry data were collected with a BD LSRFortessa (BD Biosciences) and analyzed using FlowJo software (version 10.6.2, FlowJo LLC).

OT-I T cells in the above assay were isolated in the following way from C57BL/6J.OT-I.Thy1.1 TCR transgenic mice. Naive OT-I T cells were isolated from the spleens of C57BL/6J.OT-I.Thy1.1 transgenic mice by first mashing the spleen in 5 mL of T cell medium using the gentleMACS dissociator (Miltenyi Biotec) and then filtering through 70-μm pore filters and washing once with 1 mL of phosphate buffered saline (PBS). The cell suspension was spun at 1550 RPM for 5 min at room temperature. The supernatant was discarded, and the cell pellet was treated with 1 mL of ACK lysis buffer for 2 min in the dark to remove RBC contamination. The cells were washed with 5 mL of PBS and spun at 1550 RPM for 5 min at room temperature. The supernatant was discarded, and the cell pellet was used to isolate naive $CD8^+$ T cells using the Pan T cell Isolation Kit (Miltenyi Biotec) according to the manufacturer's protocol.

T-cell–mediated cytotoxicity assays

Bulk splenocytes were obtained by homogenizing spleens from OT-I–expressing mice in 5 mL of T cell medium using a gentleMACS dissociator (Miltenyi Biotec). Following homogenization, splenocytes were filtered through a 70-μm pore filter and washed once with 1X PBS. RBCs were then lysed with ACK lysis buffer (0.15 M NH4Cl, 10 mM KHCO3, 0.5 mM EDTA). Splenocytes were enumerated and plated in flat-bottom 96-well plates (Corning Life Sciences) at a density of 0.2×10^6 cells/well in T cell medium containing 10 ng/mL SIINFEKL peptide (AnaSpec), followed by incubation at 37°C and 5% CO_2 for 3 days. Subsequently, these primed OT-I cells were washed to remove SIINFEKL peptide and cultured with fresh T cell medium supplemented with 10 ng/mL recombinant human IL-2 for an additional 2 days before use in the T cell cytotoxicity assays described below.

MC38-OVA or MC38 tumor cells were plated at a density of 5000 cells/well in 96-well U-bottom plates in a total volume of 200 μL of complete RPMI 1640. After overnight incubation to allow cells to adhere, cells were treated with cisplatin or carboplatin, with doses in 2-fold dilution starting from 75 μM. Treated cells were incubated for an additional 24 h at 37°C and 5% CO_2. Following this incubation, without removing the medium, 20 μL of preactivated OT-I T cells were added at a density of 25,000 cells/well, with a final effector-to-target cell ratio of 5:1. To monitor cell death contributed by the chemotherapy only, control plates were set up by adding 20 μL of T cell medium to the pretreated tumor cells. T-cell–mediated tumor cell killing was measured 5 h after incubation. Briefly, cells were stained with an antibody cocktail containing CD45 and CD8 for 30 min at room temperature, washed twice with FACS stain buffer (BD Biosciences), and then resuspended in 1X annexin V binding buffer containing a cocktail of annexin V PE (1:100) and 7-AAD (1:100). Tumor cell death was monitored by flow cytometry, and the data were collected with a BD LSRFortessa (BD Biosciences) and analyzed using FlowJo software (version 10.6.2, FlowJo LLC). Total cell death was calculated using the following formula: percentage T-cell–induced death with chemotherapy = (percentage tumor death in the condition with tumor cells, T cells, and chemotherapy) − (percentage tumor death in the condition of tumor cells and chemotherapy).

ELISA

ELISA was performed according to the manufacturer's instructions and included the following: human IFNγ (BD Biosciences, Cat.# 555142), mouse HMGB1 (Tecan, Cat# 30164033), and mouse IP-10 (CXCL10) (Invitrogen).

QUANTIFICATION AND STATISTICAL ANALYSIS

OS and PFS outcomes were analyzed by the Kaplan-Meier method with a log rank test. Univariate Cox regressions were implemented to estimate HRs and 95% CIs.

Statistical details of experiments, number of repeats performed, and statistical tests used are stated in the figure legends or detailed in materials and methods. Unless otherwise specified, all data are presented as mean ± SEM.

ADDITIONAL RESOURCES

Clinical trial information for IMvigor130 (NCT02807636): https://clinicaltrials.gov/study/NCT02807636.

Cell Reports Medicine, Volume *5*

Supplemental information

Immunomodulatory effects and improved outcomes with cisplatin- versus carboplatin-based chemotherapy plus atezolizumab in urothelial cancer

Matthew D. Galsky, Xiangnan Guan, Deepali Rishipathak, Aaron S. Rapaport, Hesham M. Shehata, Romain Banchereau, Kobe Yuen, Eugene Varfolomeev, Ruozhen Hu, Chia-Jung Han, Haocheng Li, Yuxin Liang, Domagoj Vucic, Li Wang, Jun Zhu, Haocheng Yu, Rebecca H. Herbst, Emma Hajaj, Evgeny Kiner, Aristotelis Bamias, Maria De Santis, Ian D. Davis, José Ángel Arranz, Eiji Kikuchi, Sandrine Bernhard, Patrick Williams, Chooi Lee, Ira Mellman, Shomyseh Sanjabi, Robert Johnston, Peter C. Black, Enrique Grande, and Sanjeev Mariathasan

Table S1. Baseline patient characteristics across different treatment arms in the CITE-Seq cohort. Related to Figure 1.

	GemCis (n=19)	GemCarbo (n=19)	Atezolizumab (n=33)	GemCis + Atezolizumab (n=15)	GemCarbo + Atezolizumab (n=27)
Age,years	62(50-74)	66(45-87)	66(51-81)	65(53.5-76.5)	67(58-76)
Age group,years					
<65	10(53%)	8(42%)	16(48%)	7(47%)	10(37%)
>=65	9(47%)	11(58%)	17(52%)	8(53%)	17(63%)
Gender					
Female	1(5%)	11(58%)	4(12%)	5(33%)	5(19%)
Male	18(95%)	8(42%)	29(88%)	10(67%)	22(81%)
Race					
White	17(89%)	15(79%)	22(67%)	13(87%)	23(85%)
Black or African American	0(<1%)	0(<1%)	0(<1%)	0(<1%)	0(<1%)
Asian	2(11%)	4(21%)	11(33%)	2(13%)	3(11%)
American Indian or Alaska Native	0(<1%)	0(<1%)	0(<1%)	0(<1%)	1(4%)
Native Hawaiian or other Pacific Islander	0(<1%)	0(<1%)	0(<1%)	0(<1%)	0(<1%)
Multiple races	0(<1%)	0(<1%)	0(<1%)	0(<1%)	0(<1%)
Unknown	0(<1%)	0(<1%)	0(<1%)	0(<1%)	0(<1%)
Tobacco use history					
Never	9(47%)	8(42%)	7(21%)	6(40%)	8(30%)
Current	4(21%)	1(5%)	7(21%)	4(27%)	6(22%)
Former	6(32%)	10(53%)	19(58%)	5(33%)	13(48%)
Primary tumor site					
Bladder	16(84%)	13(68%)	26(79%)	11(73%)	19(70%)
Urethra	0(<1%)	0(<1%)	1(3%)	1(7%)	0(<1%)
Renal pelvis	1(5%)	3(16%)	5(15%)	1(7%)	6(22%)
Ureter	2(11%)	1(5%)	1(3%)	2(13%)	2(7%)
Other	0(<1%)	2(11%)	0(<1%)	0(<1%)	0(<1%)
Disease status					
Locally advanced	0(<1%)	1(5%)	2(6%)	1(7%)	3(11%)
Metastatic	19(100%)	18(95%)	31(94%)	14(93%)	24(89%)
Site of metastatic disease					
Lymph node only	5(26%)	0(<1%)	7(21%)	4(27%)	3(11%)
Visceral	12(63%)	13(68%)	22(67%)	8(53%)	18(67%)
Liver	7(37%)	7(37%)	9(27%)	1(7%)	11(41%)
ECOG performance status					
0	11(58%)	4(21%)	15(45%)	5(33%)	8(30%)
1	7(37%)	13(68%)	16(48%)	10(67%)	13(48%)
2	1(5%)	2(11%)	2(6%)	0(<1%)	6(22%)
Programmed death-ligand 1 status on immune cells					
IC2/3	5(26%)	3(16%)	11(33%)	4(27%)	5(19%)
IC0/1	14(74%)	16(84%)	22(67%)	11(73%)	22(81%)

Data are n (%) unless indicated otherwise.

Table S2. Comparison of baseline characteristics in the CITE-Seq (n = 113) versus non–CITE-Seq cohort (N = 1100). Related to Figure 1.

	Non-scSeq patients (n=1100)	scSeq patients (n=113)
Age,years	68(56-80)	66(52-80)
Age group,years		
<65	396(36%)	51(45%)
>=65	704(64%)	62(55%)
Gender		
Female	270(25%)	26(23%)
Male	830(75%)	87(77%)
Race		
White	821(75%)	90(80%)
Black or African American	7(1%)	0(<1%)
Asian	247(22%)	22(19%)
American Indian or Alaska Native	8(1%)	1(1%)
Native Hawaiian or other Pacific Islander	1(<1%)	0(<1%)
Multiple races	1(<1%)	0(<1%)
Unknown	15(1%)	0(<1%)
Tobacco use history		
Never	403(37%)	38(34%)
Current	174(16%)	22(19%)
Former	523(48%)	53(47%)
Primary tumor site		
Bladder	782(71%)	85(75%)
Urethra	19(2%)	2(2%)
Renal pelvis	161(15%)	16(14%)
Ureter	129(12%)	8(7%)
Other	9(1%)	2(2%)
Disease status		
Locally advanced	121(11%)	7(6%)
Metastatic	979(89%)	106(94%)
Site of metastatic disease		
Lymph node only	199(18%)	19(17%)
Visceral	627(57%)	73(65%)
Liver	237(22%)	35(31%)
ECOG performance status		
0	469(43%)	43(38%)
1	511(46%)	59(52%)
2	120(11%)	11(10%)
Programmed death-ligand 1 status on immune cells		
IC2/3	256(23%)	28(25%)
IC0/1	844(77%)	85(75%)

Data are n (%) unless indicated otherwise.

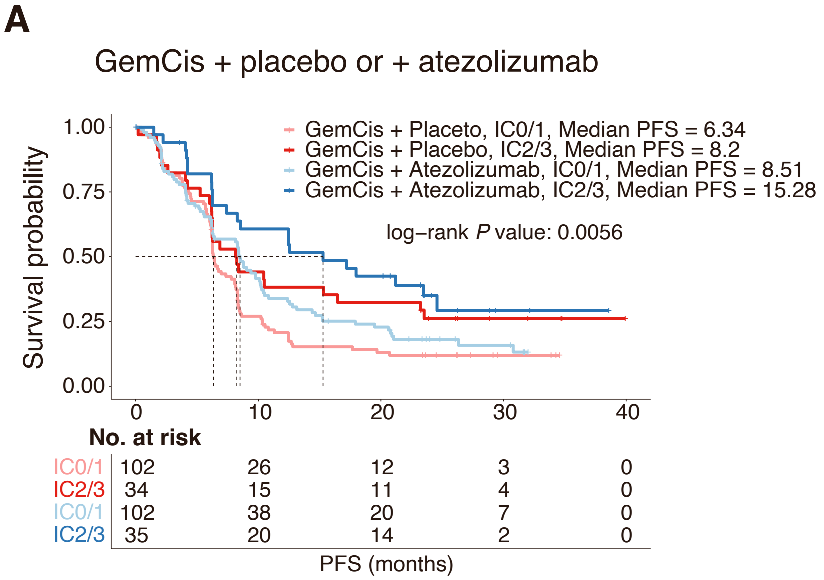

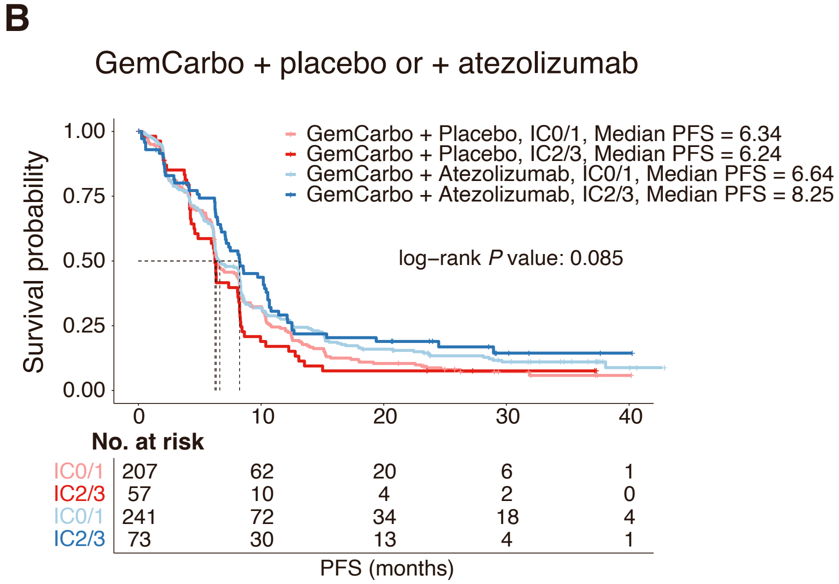

Figure S1. Progression-free survival outcomes with GemCis, but not with GemCarbo, are dependent on pre-treatment tumor PD-L1 expression. Related to Figure 1.

(A-B) Kaplan-Meier curves showing progression-free survival in patients receiving GemCis plus atezolizumab or placebo (A) or GemCarbo plus atezolizumab or placebo (B), stratified by tumor PD-L1 expression as measured by SP142 staining. *P* values were estimated using the log-rank test.

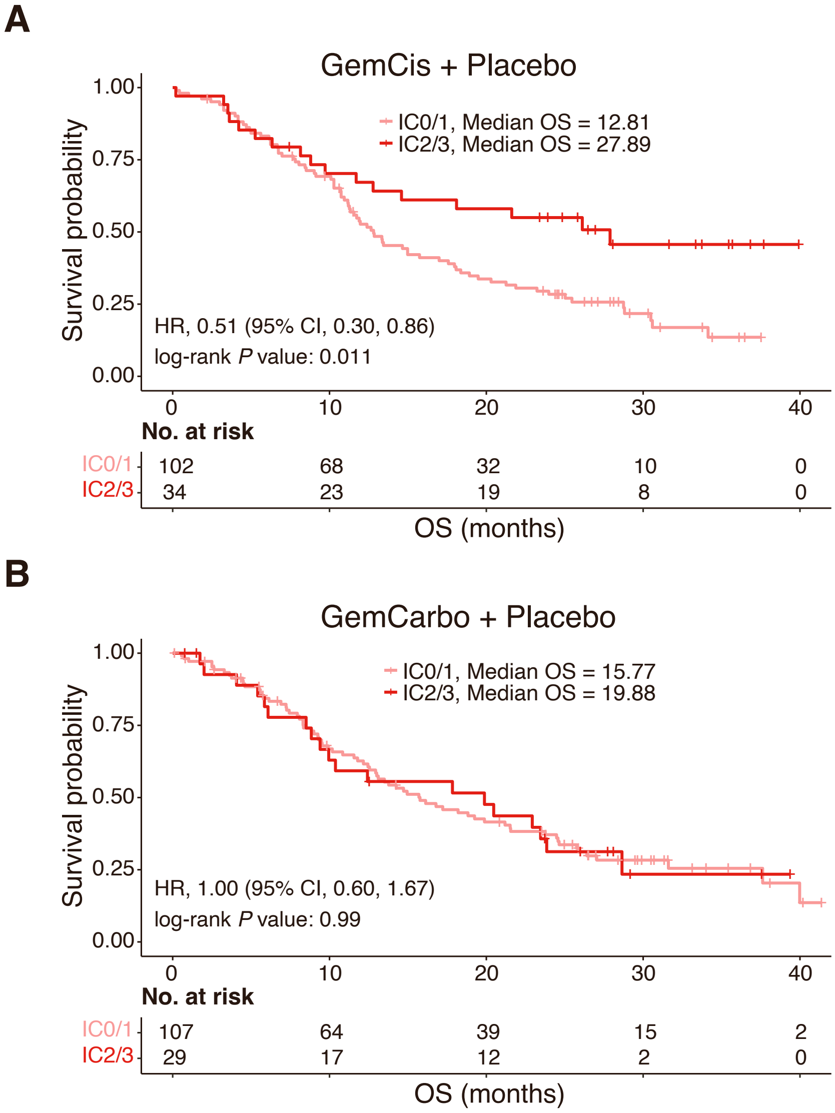

Figure S2. Survival outcomes with GemCis, but not with GemCarbo, are dependent on pre-treatment tumor PD-L1 expression in matched patients. Related to Figure 1.

(A-B) Kaplan-Meier curves showing overall survival in matched patients in arm C stratified by PD-L1 status (IC2/3 versus IC0/1) and use of GemCis (A) or GemCarbo (B). Cisplatin-eligible patients receiving GemCis or GemCarbo were matched 1:1 by propensity scores calculated using age, ECOG, Bajorin risk factors, and the site of metastasis variables. Patients whose propensity scores cannot be matched were discarded. *P* values were calculated using the log-rank test. Hazard ratio and 95% confidence interval were estimated using a univariate Cox model.

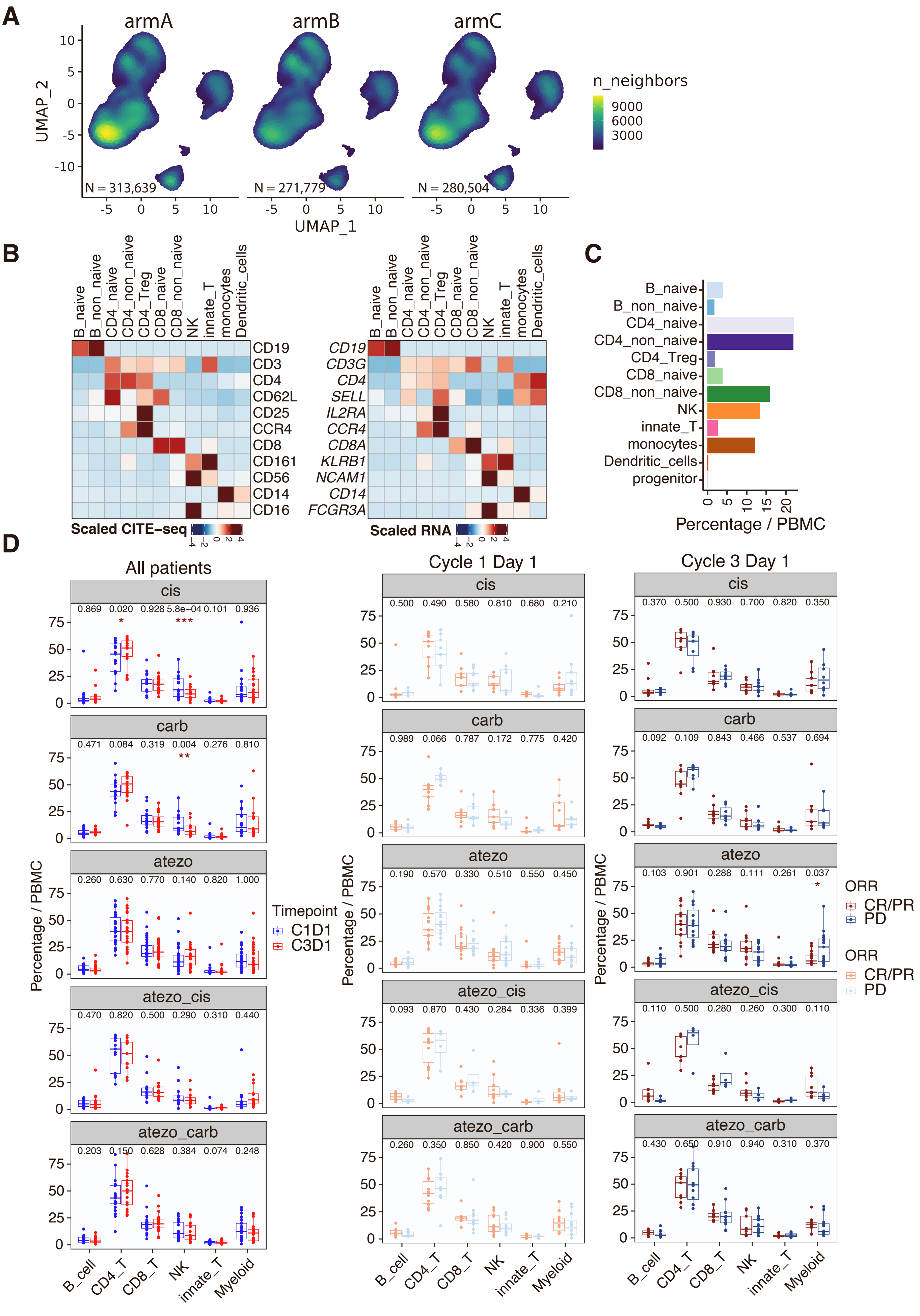
A
armA
armB
armC
UMAP_2
UMAP_1
N = 313,639
N = 271,779
N = 280,504
n_neighbors
9000
6000
3000
B
B_naive
B_non_naive
CD4_naive
CD4_non_naive
CD4_Treg
CD8_naive
CD8_non_naive
NK
innate_T
monocytes
Dendritic_cells
CD19
CD3
CD4
CD62L
CD25
CCR4
CD8
CD161
CD56
CD14
CD16
Scaled CITE–seq
CD19
CD3G
CD4
SELL
IL2RA
CCR4
CD8A
KLRB1
NCAM1
CD14
FCGR3A
Scaled RNA
C
B_naive
B_non_naive
CD4_naive
CD4_non_naive
CD4_Treg
CD8_naive
CD8_non_naive
NK
innate_T
monocytes
Dendritic_cells
progenitor
Percentage / PBMC
D
All patients
Cycle 1 Day 1
Cycle 3 Day 1
cis
0.869 0.020 0.928 5.8e–04 0.101 0.936
0.500 0.490 0.580 0.810 0.680 0.210
0.370 0.500 0.930 0.700 0.820 0.350
carb
0.471 0.084 0.319 0.004 0.276 0.810
0.989 0.066 0.787 0.172 0.775 0.420
0.092 0.109 0.843 0.466 0.537 0.694
atezo
0.260 0.630 0.770 0.140 0.820 1.000
0.190 0.570 0.330 0.510 0.550 0.450
0.103 0.901 0.288 0.111 0.261 0.037
atezo_cis
0.470 0.820 0.500 0.290 0.310 0.440
0.093 0.870 0.430 0.284 0.336 0.399
0.110 0.500 0.280 0.260 0.300 0.110
atezo_carb
0.203 0.150 0.628 0.384 0.074 0.248
0.260 0.350 0.850 0.420 0.900 0.550
0.430 0.650 0.910 0.940 0.310 0.370
Percentage / PBMC
Timepoint
C1D1
C3D1
ORR
CR/PR
PD
ORR
CR/PR
PD
B_cell
CD4_T
CD8_T
NK
innate_T
Myeloid

Figure S3. The proportions of major cell types in PBMCs remain unchanged after treatment. Related to Figure 3.
(A) UMAP embedding as shown in Figure 3B as a point-density plot split by cells from am A (N = 313,639), arm B (N = 271,779) and arm C (N = 280,504).
(B) Heatmaps showing scaled expression of the surface proteins as measured by CITE-seq (left) and the corresponding genes (right) as measured by single cell RNA-seq. The data indicated consistent expression between CITE-seq and single cell RNA-seq.
(C) Bar plot showing the abundance of different cell types in PBMCs.
(D) Box plot comparing the proportions of each cell type at baseline (C1D1) versus on-treatment (C3D1) in different treatment groups (left). Nominal P values derived from a two-tailed paired Student t-test are shown, and red asterisks represent significance (* $P < 0.05$; ** $P < 0.01$; *** $P < 0.001$). Box plots comparing the proportions of each cell type between responders (i.e., patients with complete response or partial response [CR/PR]) and nonresponders (i.e., patients with progressive disease [PD]) at baseline (C1D1, middle) and on-treatment (C3D1, right) in different treatment groups. Nominal P values derived from the two-tailed unpaired Student t-test are shown.

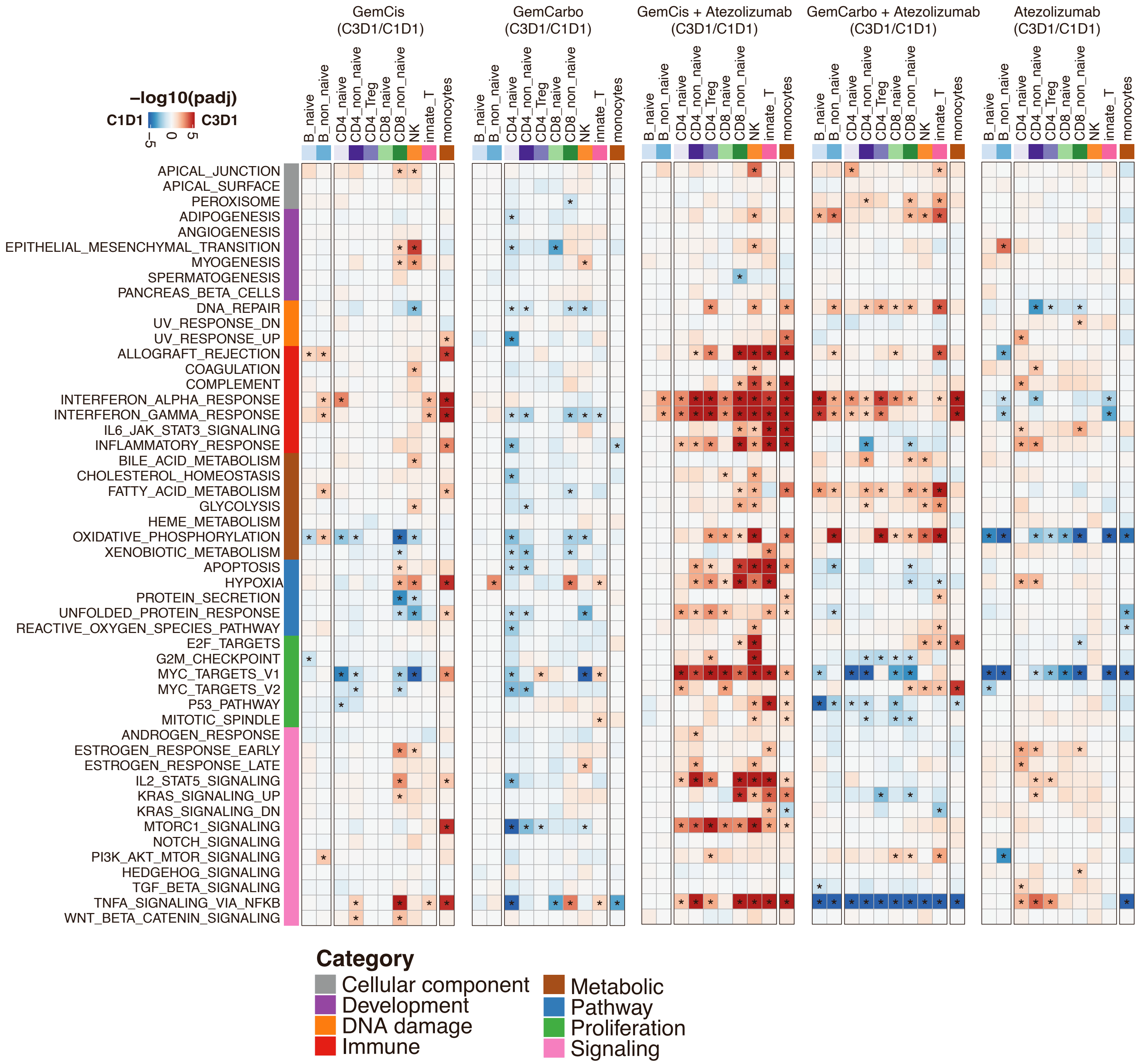

Figure S4. GemCis with and without atezolizumab versus GemCarbo with and without atezolizumab induces proinflammatory transcriptional programs across multiple immune cell subsets in PBMCs. Related to Figure 3.
Heatmaps comparing the pathway enrichment of all 50 Hallmark gene sets on-treatment (C3D1) versus baseline (C1D1) across multiple immune cell types in different treatment groups. Red indicates enrichment in on-treatment samples, and blue indicates enrichment at baseline. The hue represents the false-discovery rate (FDR) significance, derived from the fgsea package. Black asterisks represent FDR <0.05.

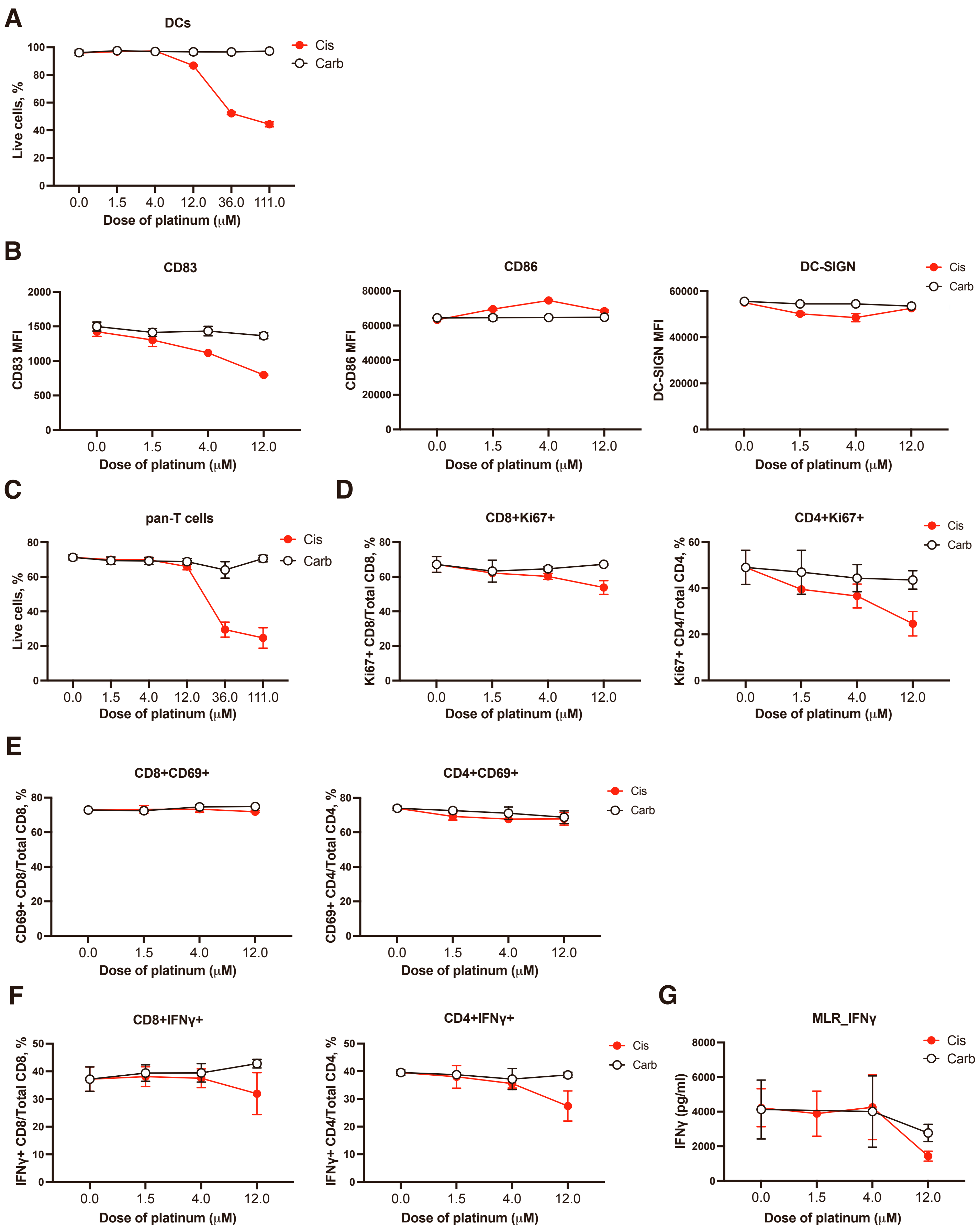
A
DCs
Live cells, %
100
80
60
40
20
0
0.0
1.5
4.0
12.0
36.0
111.0
Dose of platinum (μM)
Cis
Carb
B
CD83
CD83 MFI
2000
1500
1000
500
0
CD86
CD86 MFI
80000
60000
40000
20000
0
DC-SIGN
DC-SIGN MFI
60000
40000
20000
0
0.0
1.5
4.0
12.0
Dose of platinum (μM)
Cis
Carb
C
pan-T cells
Live cells, %
80
60
40
20
0
0.0
1.5
4.0
12.0
36.0
111.0
Dose of platinum (μM)
Cis
Carb
D
CD8+Ki67+
Ki67+ CD8/Total CD8, %
CD4+Ki67+
Ki67+ CD4/Total CD4, %
Cis
Carb
E
CD8+CD69+
CD69+ CD8/Total CD8, %
CD4+CD69+
CD69+ CD4/Total CD4, %
Cis
Carb
F
CD8+IFNγ+
IFNγ+ CD8/Total CD8, %
CD4+IFNγ+
IFNγ+ CD4/Total CD4, %
50
40
30
20
10
0
Cis
Carb
G
MLR_IFNγ
IFNγ (pg/ml)
8000
6000
4000
2000
0
Cis
Carb
Dose of platinum (μM)

Figure S5. Cisplatin and carboplatin at sub-cytotoxic levels do not have direct effects on the activation and function of DCs and T cells. Related to Figure 5.

(A) Average percentages of live DCs treated with increasing concentrations of cisplatin or carboplatin (n = 3).

(B) Representative plots of CD83, CD86, and DC-SIGN surface protein expression in DCs treated with increasing concentrations of cisplatin or carboplatin (n = 3).

(C) Average percentages of live pan-T cells with increasing concentrations of cisplatin or carboplatin (n = 3).

(D) Representative plots of the percentages of proliferating CD8 (left) and CD4 (right) T cells, as measured by Ki67, after treatment with increasing concentrations of cisplatin or carboplatin (n = 3).

(E) Representative plots of the percentages of activated CD8 (left) and CD4 (right) T cells, as measured by CD69, after treatment with increasing concentrations of cisplatin or carboplatin (n = 3).

(F) Representative plots of the percentages of IFNγ+ CD8 (left) and CD4 (right) T cells after treatment with increasing concentrations of cisplatin or carboplatin (n = 3).

(G) Representative plots of IFNγ secretion as measured in the supernatant from the co-culture of DC cells with pan-T cells treated with increasing concentrations of cisplatin or carboplatin (n = 4). In (A-G), error bars represent standard deviation.

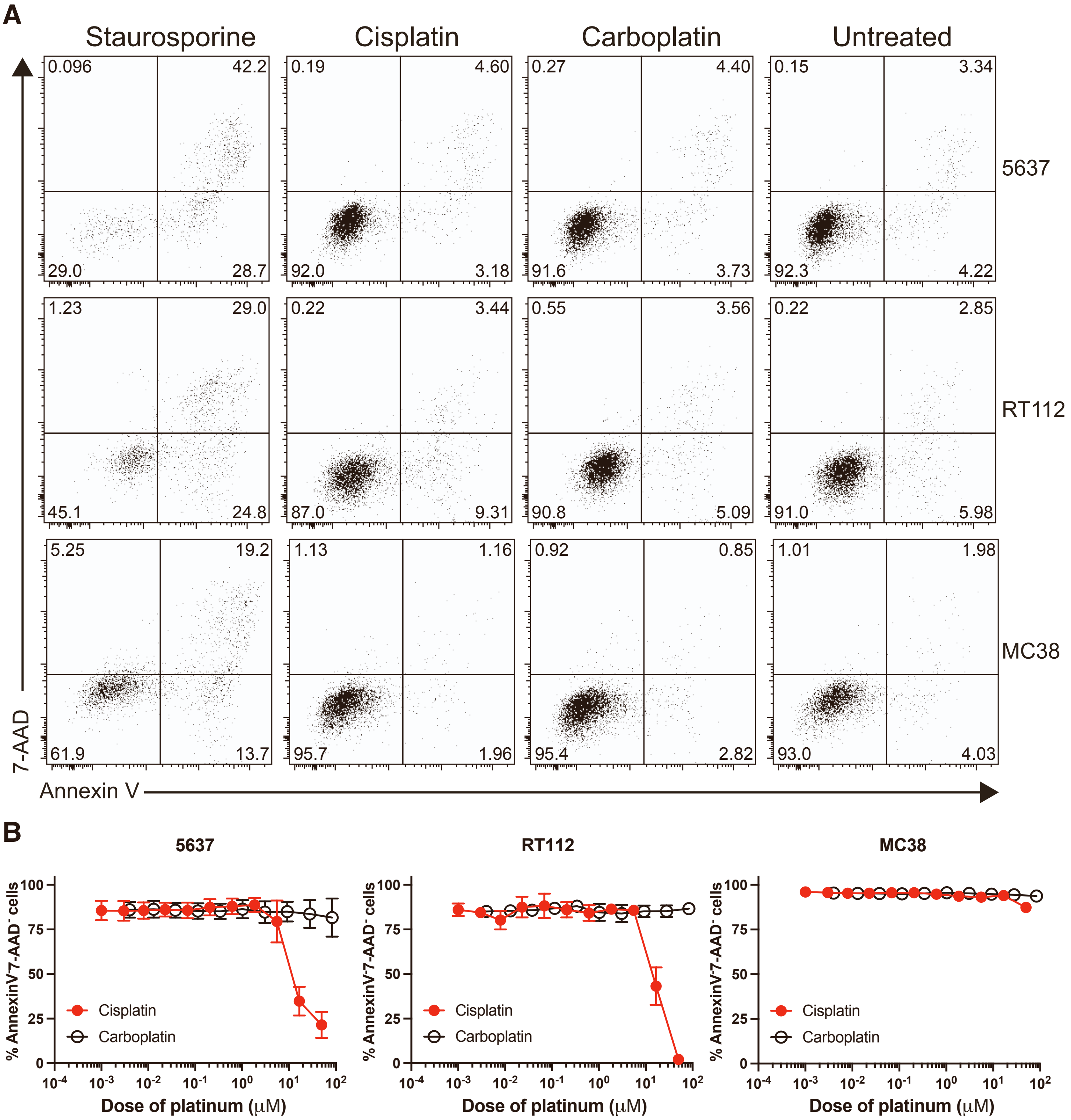

Figure S6. Cisplatin and carboplatin do not induce apoptosis within the range of clinically relevant concentrations (i.e., approximating peak serum concentrations achieved in humans at doses used to treat UC) in human urothelial bladder cancer (5637 and RT112) and murine colon adenocarcinoma (MC38) cell lines. Related to Figure 5.

(A) Representative flow cytogram showing tumor cell death as measured by 7-AAD and annexin V in 5637 (top), RT112 (middle), and MC38 (bottom) cell lines treated for 24 hours with staurosporine (125 nM, positive control for apoptosis induction), cisplatin (5 μM), or carboplatin (30 μM). Data depict one representative experiment of two independent experiments.

(B) Cells were treated with dose titrations of cisplatin or carboplatin. The proportions of non-apoptotic (annexin V–negative and 7-AAD–negative) cells were quantified at 24 hours posttreatment (n = 2). Data depict the aggregate of three independent experiments (mean ± SEM).

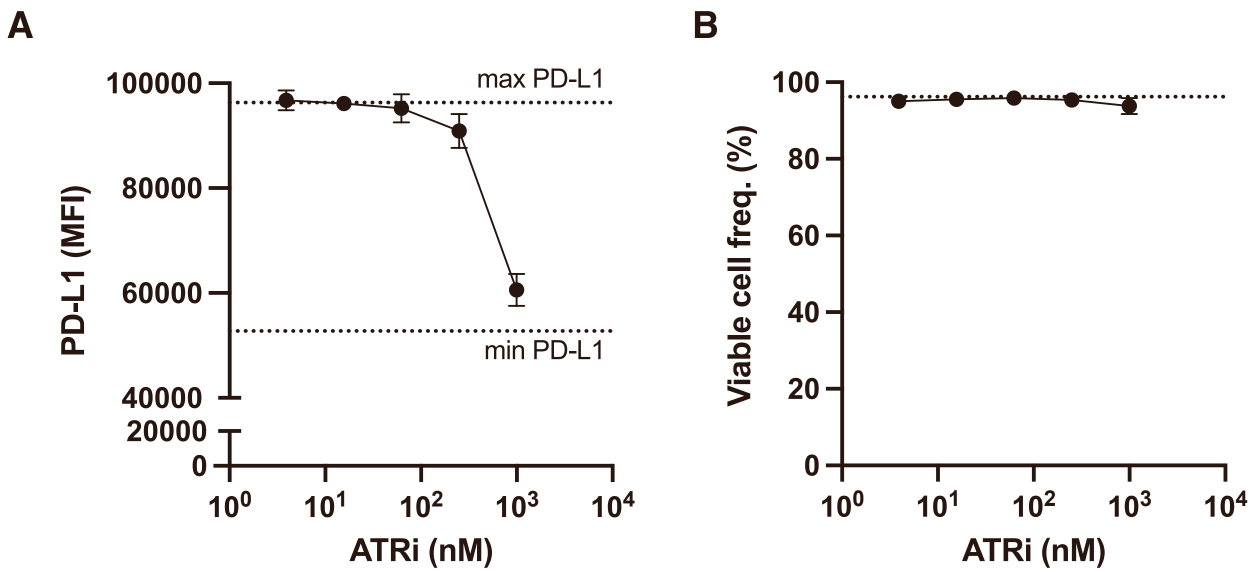

Figure S7. ATR inhibition by VE-821 is dose dependent and does not induce cell death. Related to Figure 5.
(A) Protein expression of PD-L1 as determined by median fluorescence intensity in the 5637 cells that were pre-treated with a dose titration of VE-821 followed by the addition of 5μM of cisplatin. Maximum and minimum dotted lines refer to PDL1 MFI in cisplatin-alone stimulated cells and untreated cells, respectively.
(B) Viability of the same cells as in (A), measured by 7-AAD dye incorporation. Dotted line refers to viability in untreated cells. Data depict the aggregate of two independent experiments (mean ± SEM).

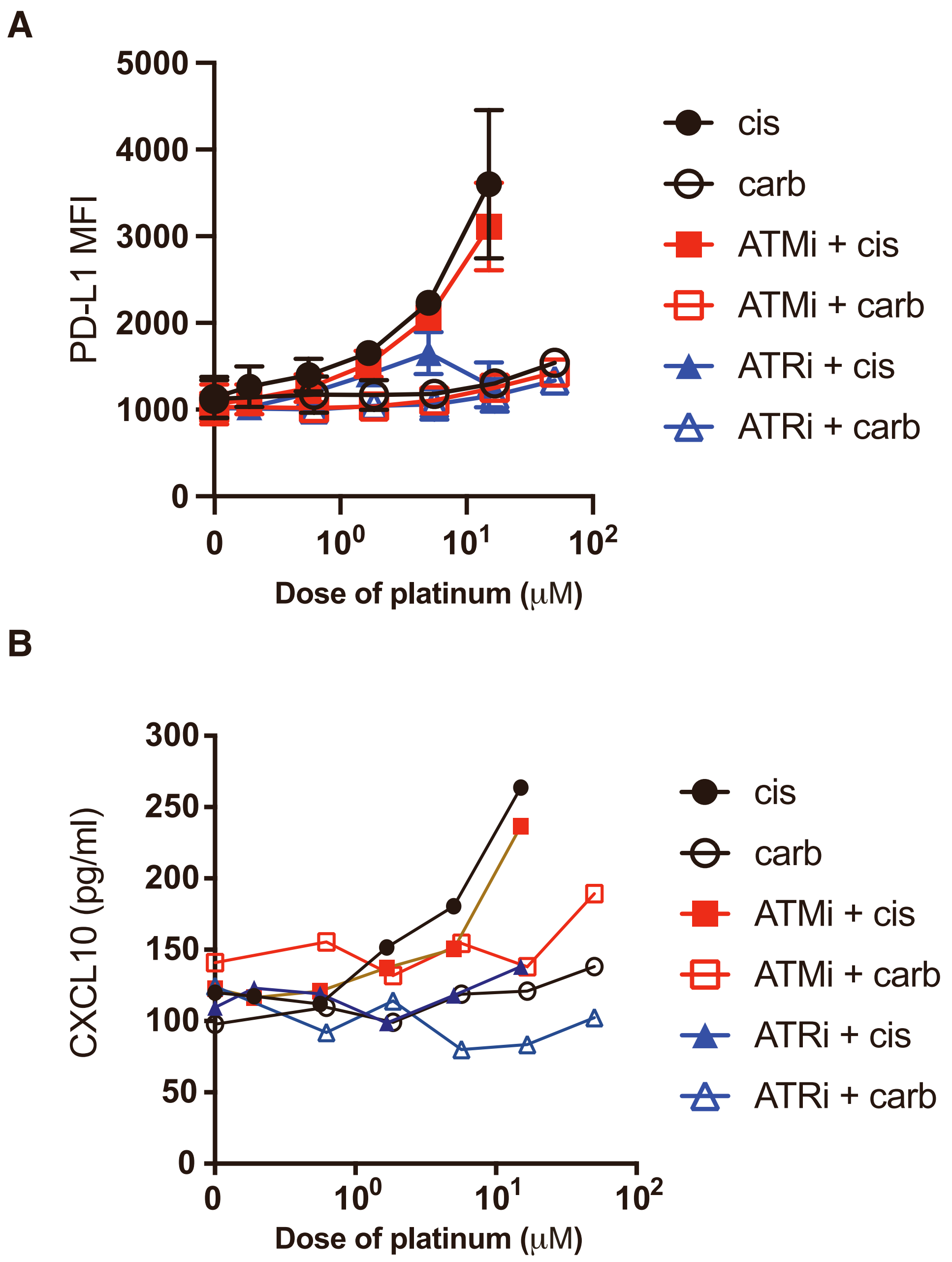

Figure S8. The direct induction of PD-L1 and CXCL10 in MC38 cells is mediated via the DNA damage transducer ATR. Related to Figure 5.

(A) Protein expression of PD-L1 as determined by median fluorescence intensity in the MC38 cell line treated with increasing concentrations of cisplatin or carboplatin for 24 hours, in the presence or absence of an ATM inhibitor (1 μM) and ATR inhibitor (1 μM) ($n = 3$). Data depict the aggregate of two independent experiments (mean ± SEM).

(B) CXCL10 (IP-10) secretion as measured by Mouse IP-10 ELISA in the supernatant collected from MC38 cells treated with increasing concentrations of cisplatin or carboplatin in the presence or absence of an ATM inhibitor (1 μM) and ATR inhibitor (1 μM). Supernatant was collected 24 hours after treatment. Data depict one experiment with duplicate conditions.

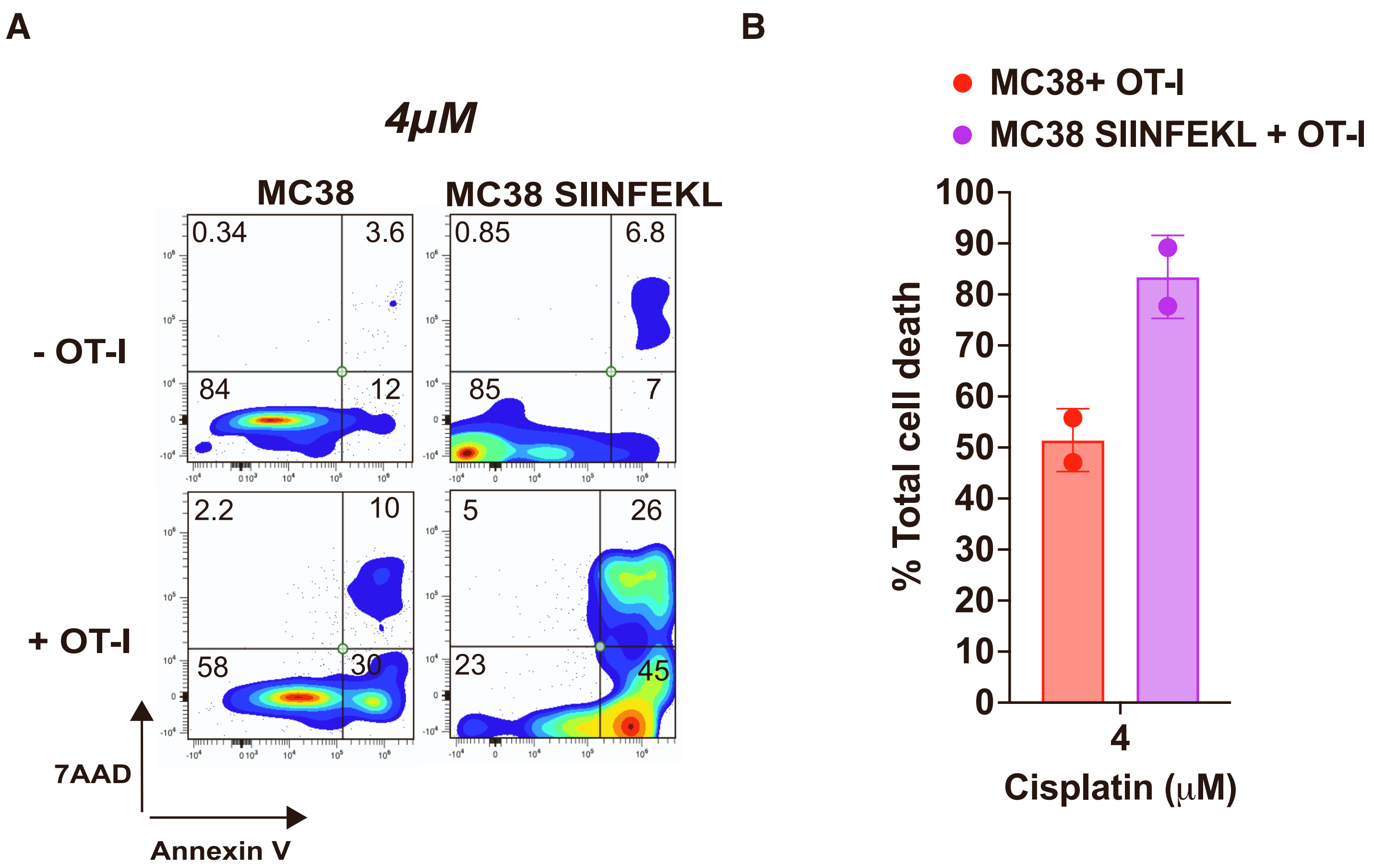

Figure S9. OT-I T-cell–mediated killing of MC38 tumor cells that express or do not express SIINFEKL OVA peptide. Related to Figure 6.

(A) Representative flow cytometry plots showing OT-I mediated cell killing of MC38 and MC38-SIINFEKL as measured by 7-AAD and annexin V staining within the tumor cells.

(B) The summarized percentage of OT-I T-cell–mediated tumor cell death. MC38 tumor cells were pretreated with or without 4uM of cisplatin for 24 hours, followed by co-culture with OT-I T cells for 5 hours before the assay. Data are mean ± SD.

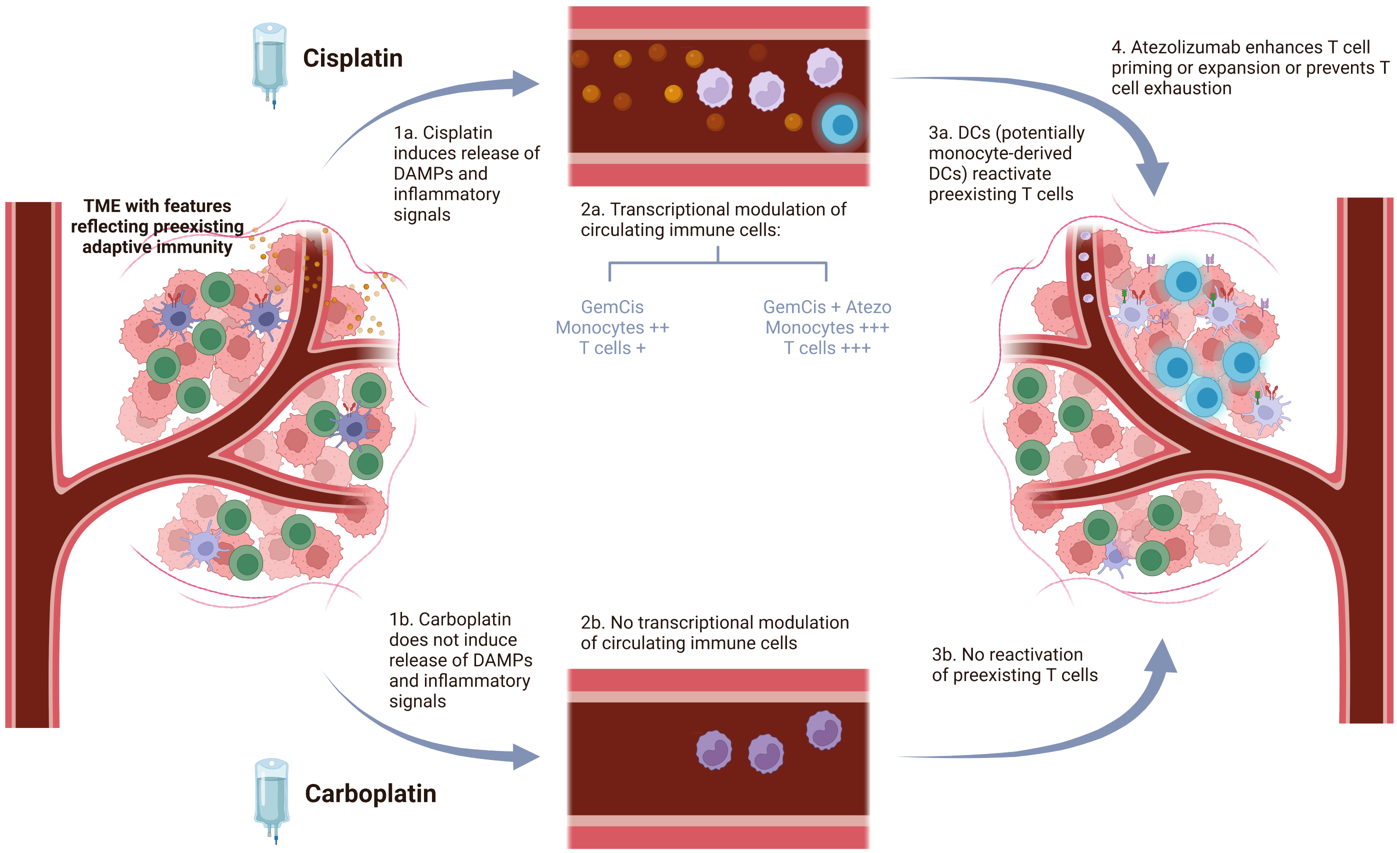

Figure S10. Proposed model for effects of cisplatin with and without atezolizumab on the cancer-immunity cycle. Related to Figures 1-6.
The model proposed involves direct modulation of cisplatin vs carboplatin on cancer cells (1), and secondary modulation on monocytes / DCs and T cells in the peripheral blood (2), ultimately leading to an enhanced immune niche favoring cancer cell killing (3), which is further enhanced by atezolizumab preventing T cell exhaustion (4). DAMP, danger-associated molecular pattern; DC, dendritic cell; GemCarbo, gemcitabine plus carboplatin; GemCis, gemcitabine plus cisplatin; TME, tumor microenvironment.
